# Identification and comparative expression analysis of odorant-binding proteins in the reproductive system and antennae of *Athetis dissimilis*

**DOI:** 10.1038/s41598-021-93423-1

**Published:** 2021-07-06

**Authors:** Yue-Qin Song, Zhi-Yu Song, Jun-Feng Dong, Qi-Hui Lv, Qing-Xiao Chen, Hui-Zhong Sun

**Affiliations:** College of Horticulture and Plant Protection, Henan University of Science and Technology, Luoyang, 471000 China

**Keywords:** Genetics, Molecular biology, Zoology

## Abstract

Odorant-binding proteins (OBPs) are prevalent in the antennal transcriptomes of different orders of insects. Studies on OBPs have focused on their role in the insect chemosensory system, but knowledge of their functions in the insect testis is limited. We sequenced the transcriptomes of the *Athetis dissimilis* reproductive organs and analyzed the expression of AdisOBP genes in different tissues. We identified 23 OBPs in the testis and ovaries and 31 OBPs in antennal transcriptomes. The results of real-time quantitative PCR revealed that 23 of the 54 OBP genes were highly expressed in both female and male antennae, including three that exhibited male-biased expression and 15 that exhibited female-biased expression. A total of 24 *OBPs* were highly expressed in the testis of *A. dissimilis*, while expression of *OBPs* in the ovaries was very low. These findings highlight the functional diversity of OBPs in insects and can facilitate further studies on the OBPs in *A. dissimilis* and lepidopteran species.

## Introduction

The olfactory system in insects regulates their intersex communication, host-plant interactions, oviposition, foraging, escape from predators and reproduction^1–5^. Insects have a complex chemosensory system in which pheromones and plant odors are initially recognized by odorant-binding proteins (OBPs) expressed in the antennal sensilla lymph that transfer the odorants to membrane-bound olfactory receptors (ORs) to activate olfactory receptor neurons (ORNs) and stimulate behavioral responses^6–11^.

Understanding the molecular mechanisms of olfaction is essential for better using olfactory-based pest management strategies and the development of novel strategies. OBPs are more accessible targets for research, considering they are small, soluble, stable and easier to manipulate and modify. OBPs are small water soluble proteins that have six positionally conserved cysteines to form three interlocking disulphide bridges that stabilize the protein’s three-dimensional structure^12–19^. OBPs were first discovered in the antenna of *Antheraea polyphemus*, where they distinguish and bind to lipophilic odorant compounds^20–25^. However, emerging data suggests that OBPs are not restricted to the sensory organs of insect and show expression in non-sensory organs including reproductive organs^26,27^. Li et al. showed that *AaegOBP22* was highly expressed in the male reproductive organs of *Aedes aegypti* and transfers to females during mating. This suggests an additional function for this protein as pheromone carrier, analogously to vertebrates’ urinary and salivary proteins as well as some insect chemosensory proteins^26^. Sun et al. also found that *HarmOBP10* and *HassOBP10* is highly abundant in seminal fluid of *Helicoverpa armigera* and *H. assulta* and transfers to female during mating. HarmOBP10 and HassOBP10 also bind 1-dodecene, a known insect repellent^27^.

*Athetis dissimilis* Hampson (Lepidoptera: Noctuidae) is an important agricultural pest and mainly distributed in Asian countries including China, Japan, Philippines, Korea, Indonesia and India, causing serious damages to maize, wheat, peanut, soybean and sweet potato^28–30^. Because of the fact that larvae of *A. dissimilis* live under plant residues, it is difficult to control the spread of the pest with chemical pesticides. Therefore, novel control managements are urgently needed to mitigate crop damage. We first sequenced the antennal transcriptomes of *A. dissimilis*^31^ and characterized 5 OBPs that showed tissue-specific expression patterns^32^. Of note, *AdisOBP6* was highly expressed in the testes of *A. dissimilis*^32^. We reasoned that the testis of insects possess a defined set of OBPs in a manner comparable to the antenna. In this study, we reanalyzed the previous antennal transcriptome data and identified 31 OBP genes. We also sequenced the transcriptomes of the *A. dissimilis* reproductive organs, and studied the expression of the OBPs in the antennae, testis and ovaries. Our study provides a new reference for studying the function of OBP genes.

## Results

### Illumina sequencing and assembly

A total of 34,565,866, 32,154,799, and 26,952,526 clean reads containing 10.35, 9.63, and 8.07 giga base (Gb) pairs of clean nucleotides respectively, were obtained from the three replicates of the *A. dissimilis* ovaries. A total of 27,752,168, 28,900,040, and 30,838,686 clean reads containing 8.29, 8.65 and 9.23 giga base (Gb) pairs of clean nucleotides respectively, were obtained from the three replicates of *A. dissimilis* testes. The quality of the transcriptome sequences was high, with Q30 percentages of 94.03%, 94.36%, 94.21%, 94.42%, 94.27% and 94.01% for the three replicates of *A. dissimilis* ovaries and testes, with a GC content of ~ 50% (Table 1). Then 221,074 transcripts and 82,016 unigenes with N50 length of 1350 and 1243 were obtained from assembled using Trinity (Table 2).

**Table 1:** Summary of the sequence assemblies according to the RNA-seq data of the *A. dissimilis*.

Sample name	Clean reads	Clean bases	GC content (%)	Q30 (%)
**Ovaries**				
Repeat 1	34,565,866	10.35 G	48.00	94.03
Repeat 2	32,154,799	9.63 G	48.35	94.36
Repeat 3	26,952,526	8.07 G	48.27	94.21
**Testis**				
Repeat 1	27,752,168	8.29 G	48.85	94.42
Repeat 2	28,900,040	8.65 G	47.20	94.27
Repeat 3	30,838,686	9.23 G	46.65	94.01

**Table 2:** Summary of de novo assembly of the *A. dissimilis* transcriptomes.

Length range	Transcript	Rate%	Unigene	Rate%
< 300	0	0	0	0
300–500	83,670	37.85	37,104	45.24
500–1000	70,088	31.70	24,792	30.23
1000–2000	44,935	20.33	12,864	15.68
> 2000	22,381	10.12	7256	8.85
Total number	221,074		82,016	
Total length	216,261,287		73,549,396	
N50 length	1350		1243	
Mean length	978.23		896.77	

### Functional annotation

**Figure 1:**
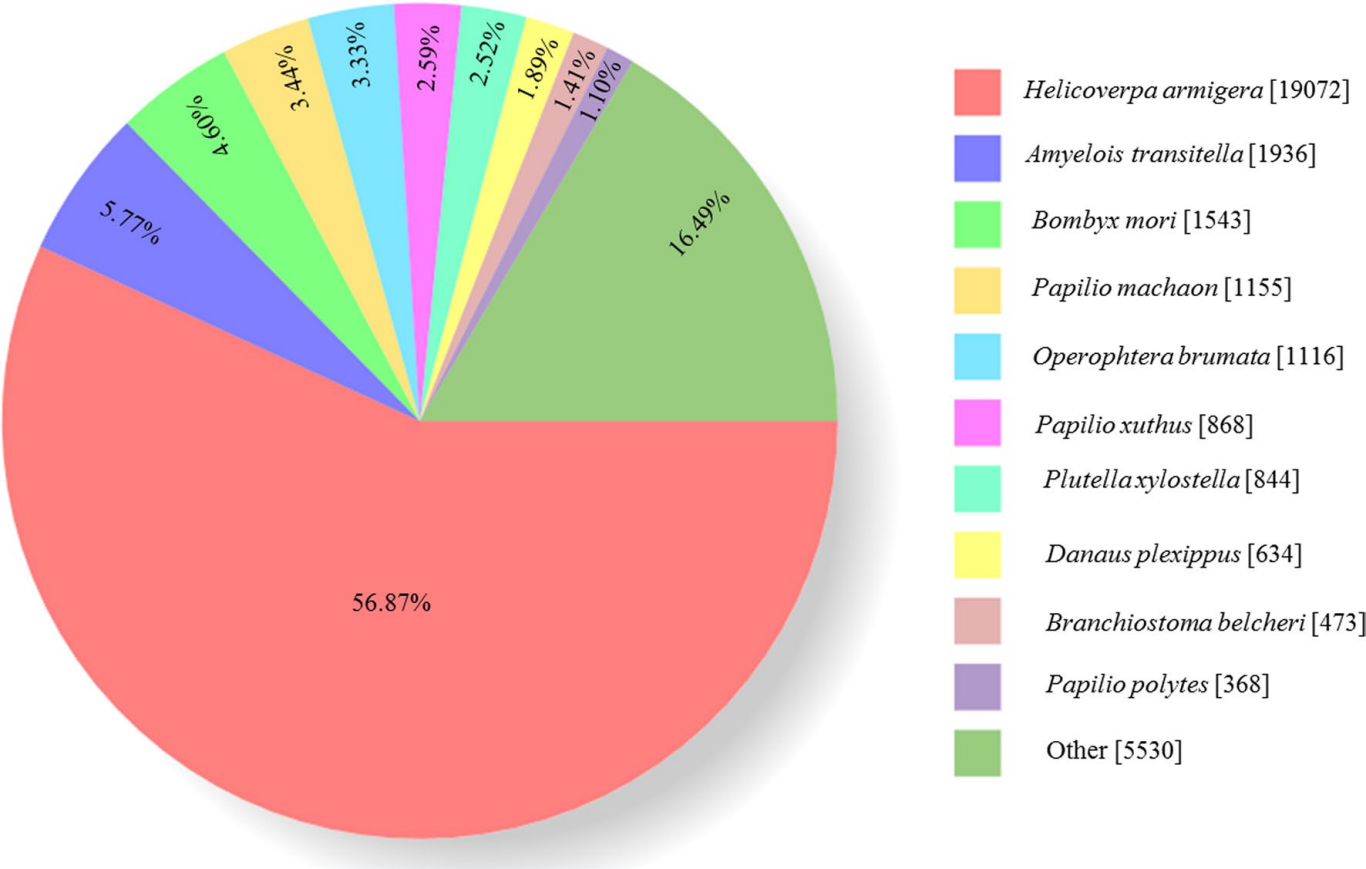
The Blastx results of *Athetis dissimilis* reproductive organs unigenes in NR database.

Significant matches of 33,587 unigenes (96.91%) in the NR; 29,936 (86.38%) in the eggnog; 20,134 (58.09%) in the Pfam; 15,174 (43.78%) in the Swissprot database; 14,775 (42.63%) in the KEGG; 7797 (22.50%) in the GO; and 6712 (19.37%) in the COG were observed. As a result, up to 34,658 putative coding sequences were identified (Table 3). NR database queries revealed a high percentage of *A. dissimilis* sequences that closely matched to sequences of *H. armigera* (19,072, 56.87%), *Amyelois transitella* (1936, 5.77%), *Bombyx mori* (1543, 4.60%), *Papilio machaon* (1155, 3.44%), *Papilio xuthus* (868, 2.59%), *Plutella xylostella* (844, 2.52%), *Danaus plexippus* (634, 1.89%), *Branchiostoma belcheri* (473, 1.41%), and *Papilio polytes* (368, 1.10%) (Fig. 1).

**Table 3:** Functional annotation of the *A. dissimilis* transcriptomes.

Database	Number	Rate (%)	300 ≦ length < 1000	Length ≧ 1000
COG	6712	19.37	2638	4074
GO	7797	22.50	4453	3344
KEGG	14,775	42.63	8205	6570
Pfam	20,134	58.09	8577	11,557
Swissprot	15,174	43.78	6987	8187
eggNOG	29,936	86.38	16,283	13,653
NR	33,587	96.91	18,939	14,648
All	34,658		19,914	14,744

**Figure 2:**
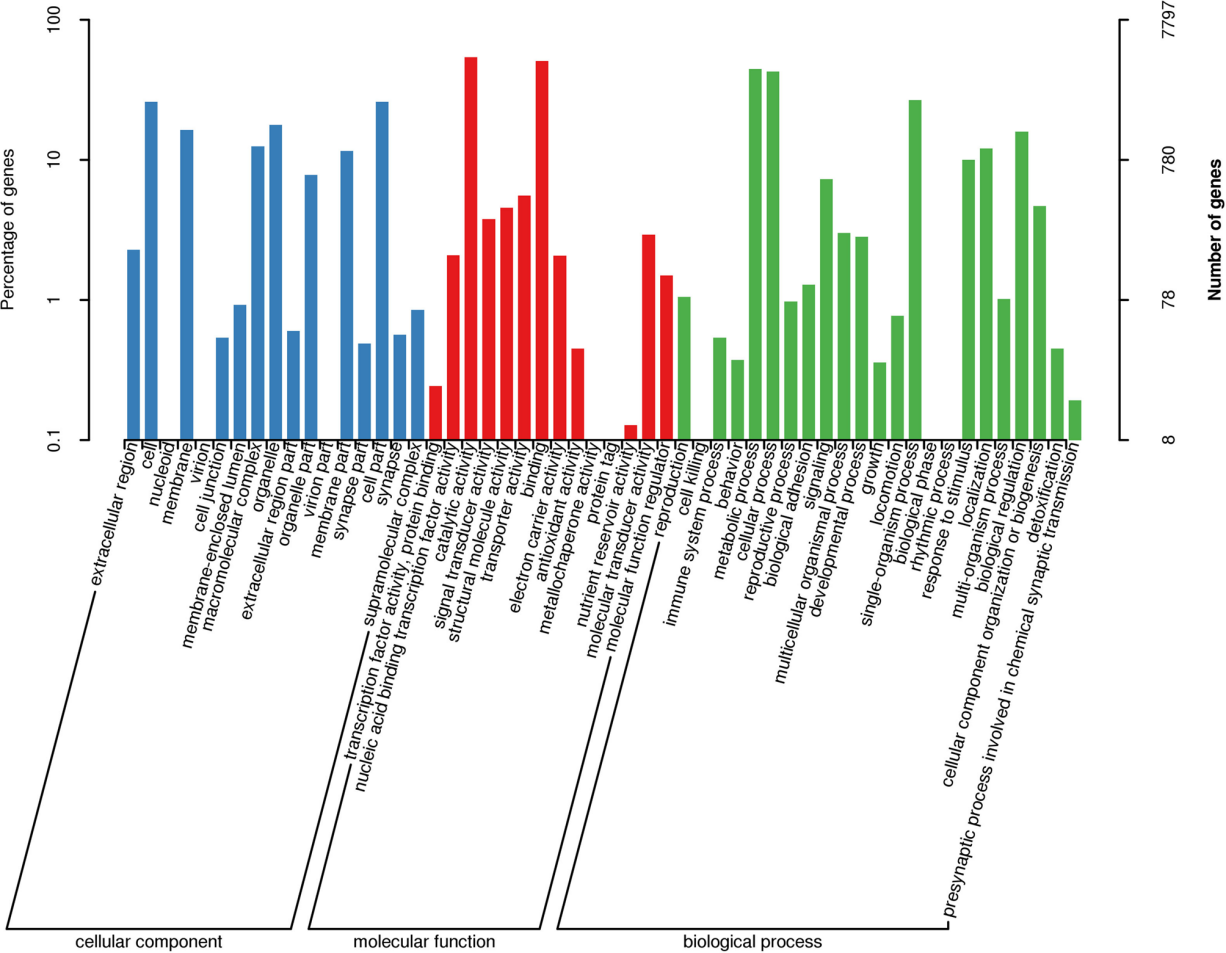
Gene Ontology (GO) classifications of *Athetis dissimilis* reproductive organs unigenes according to their involvement in biological processes, cellular component and molecular function.

For GO analysis, 7797 unigenes (22.50%) could be assigned to three GO terms including: cellular components, molecular functions and biological process (Fig. 2). For the “molecular functions” ontology, catalytic activity (4227, 42.19%) and binding (3972, 39.64%) were most prevalent.

### Identification of putative odorant-binding proteins

In the *A. dissimilis* antennal and reproductive organ transcriptome, we identified 54 candidate OBPs (Genbank accession number: KR780027–KR780030, MH900289–MH900338), 31 of which were from the antennae (through the analysis of previous *A. dissimilis* antennal transcriptomes) and 23 from the testis and ovaries transcriptomes of *A. dissimilis* (Table 4). A total of 44 *Adis*OBP sequences had full-length ORFs. Their cDNAs encoded protein of 131–293 amino acids with molecular weights of 11.6–33.2 kDa and isoelectric points of 4.44–9.74. Excluding 7 *Adis*OBPs (*Adis*OBP28, 30, 31, 35, 36, 41, 42, 52, 53 and 54) signal peptides were predicted at the N-terminus. *Adis*OBPs had 39–99% sequence homology with previously identified OBPs from other insect species, displaying a high level of sequence similarity. For example, *Adis*OBP13 had a 95% identity with *Spodoptera exigua* OBP9 (Table 4). There was 11.87% the lowest identity level in a pairwise comparison of *Adis*OBPs.

**Table 4:** The characteristic of candidate OBP genes in the antennae and reproductive organs of *A. dissimilis*.

Order	Gene name	GenBank accession no.	ORF (aa)	Molecular weight (kD)	Isoelectric point	Signal peptide	Full length	Homology search with the known proteins					
								Gene annotation	Species	Protein ID	Score	E-value	Identity (%)
c69042	AdisPBP1	KR780029	166	17.32	5.19	Yes	Yes	PBP1	*Mamestra brassicae*	AAC05702	266	3e−89	79
c65047	AdisPBP2	KR780030	162	18.08	5.30	Yes	Yes	PBP2	*Mamestra brassicae*	AAC05701	281	4e−95	81
c65143	AdisPBP3	MH900289	164	18.71	5.25	Yes	Yes	PBP3	*Agrotis ipsilon*	AFM36758	292	1e−99	82
c47645	AdisGOBP1	KR780027	163	18.89	5.19	Yes	Yes	GOBP1	*Sesamia inferens*	AGS36742	289	3e−98	99
c60029	AdisGOBP2	KR780028	161	18.09	5.09	Yes	Yes	GOBP2	*Agrotis ipsilon*	AFM36760	297	2e−101	88
c68783	AdisOBP1	MH900290	293	33.20	5.76	Yes	Yes	OBP	*Bombyx mori*	NP_001153663	264	1e−84	51
c69959	AdisOBP2	MH900291	246	27.36	5.40	Yes	Yes	OBP10	*Ostrinia furnacalis*	BAV56797	310	4e−104	66
c60098	AdisOBP3	MH900292	145	16.22	8.37	Yes	Yes	OBP	*Spodoptera exigua*	ADY17886	251	5e−84	79
c65852	AdisOBP5	MH900293	242	26.78	6.33	Yes	Yes	OBP35	*Dendrolimus punctatus*	ARO70194	215	2e−66	46
c72710	AdisOBP8	MH900294	240	27.01	6.53	Yes	Yes	OBP25	*Spodoptera exigua*	AKT26502	305	3e−102	63
c61153	AdisOBP9	MH900295	167	18.50	4.51	Yes	Yes	OBP10	*Sesamia inferens*	AGS36751	233	3e−76	79
c60049	AdisOBP11	MH900296	141	16.38	4.47	Yes	Yes	OBP8	*Spodoptera exigua*	AGH70104	232	2e−76	86
c65401	AdisOBP13	MH900297	133	15.14	9.01	Yes	Yes	OBP9	*Spodoptera exigua*	AGH70105	261	6e−88	95
c58306	AdisOBP14	MH900298	185	20.13	6.04	Yes	Yes	OBP1	*Agrotis ipsilon*	AGR39564	279	1e−93	74
c64058	AdisOBP15	MH900299	146	16.43	6.29	Yes	Yes	OBP6	*Agrotis ipsilon*	AGR39569	238	4e−79	88
c53621	AdisOBP16	MH900300	118	–	–	–	Internal	OBP18	*Spodoptera exigua*	AKT26496	124	2e−33	48
c68160	AdisOBP17	MH900301	252	28.95	6.19	Yes	Yes	OBP23	*Spodoptera exigua*	AKT26500	442	7e−156	81
c67912	AdisOBP18	MH900302	203	22.50	5.69	Yes	Yes	OBP19	*Helicoverpa assulta*	AGC92793	245	9e−80	62
c60881	AdisOBP19	MH900303	139	14.55	8.58	Yes	Yes	OBP5	*Agrotis ipsilon*	AGR39568	168	4e−51	62
c71719	AdisOBP20	MH900304	139	15.69	7.52	Yes	Yes	OBP8	*Spodoptera litura*	AKI87969	257	2e−86	87
c65033	AdisOBP21	MH900305	147	15.65	4.90	Yes	Yes	OBP5	*Helicoverpa armigera*	AEB54581	221	6e−72	75
c63129	AdisOBP22	MH900306	146	15.92	7.53	Yes	Yes	OBP23	*Spodoptera litura*	XP_022826767	238	2e−78	77
c57331	AdisOBP23	MH900307	149	15.96	5.03	Yes	Yes	OBP26	*Spodoptera exigua*	AKT26503	233	1e−76	76
c64709	AdisOBP24	MH900308	148	16.77	5.45	Yes	Yes	OBP7	*Helicoverpa armigera*	AEB54591	187	5e−57	57
c81048	AdisOBP25	MH900309	71	–	–	–	Internal	OBP22	*Spodoptera exigua*	AKT26499	130	3e−37	87
c53707	AdisOBP26	MH900310	134	14.28	4.51	Yes	Yes	OBP34	*Helicoverpa assulta*	ASA40070	225	9e−74	86
c28876	AdisOBP27	MH900311	124	–	–	–	Internal	OBP11	*Spodoptera exigua*	AGP03457.1	219	3e−71	81
c67118	AdisOBP28	MH900312	236	27.80	4.90	No	Yes	OBP9	*Spodoptera litura*	ALD65883	383	1e−131	82
c57589	AdisOBP29	MH900313	129	–	–	–	5ʹ lose	OBP33	*Helicoverpa assulta*	ASA40072	208	5e−67	76
c62521	AdisOBP30	MH900314	180	20.26	4.84	No	Yes	OBP9	*Helicoverpa armigera*	AEB54592	167	3e−50	54
c63839	AdisOBP31	MH900315	116	12.77	6.12	No	Yes	OBP14	*Spodoptera exigua*	AGP03460	199	7e−64	83
Gene.53346	AdisOBP32	MH900316	184	20.65	6.32	Yes	Yes	GOBP70	*Helicoverpa armigera*	XP_021188671	375	1e−131	98
Gene.77161	AdisOBP33	MH900317	207	23.94	9.19	Yes	Yes	OBP19	*Helicoverpa assulta*	AGC92793	151	1e−42	39
Gene.60926	AdisOBP34	MH900318	193	22.42	5.48	Yes	Yes	OBP9	*Cnaphalocrocis medinalis*	ALT31639	289	5e−97	70
Gene.32069	AdisOBP35	MH900319	137	15.34	8.85	No	Yes	OBP	*Helicoverpa armigera*	AEX07279	238	7e−79	88
Gene.44893	AdisOBP36	MH900320	143	15.92	5.57	No	Yes	OBP19	*Helicoverpa assulta*	AGC92793	187	1e−57	66
Gene.35132	AdisOBP37	MH900321	102	–	–	–	5ʹ lose	OBP24	*Cnaphalocrocis medinalis*	ALT31654	182	7e−58	86
Gene.54044	AdisOBP38	MH900322	141	15.05	8.77	Yes	Yes	OBP5	*Agrotis ipsilon*	AGR39568	155	6e−46	57
Gene.7082	AdisOBP39	MH900323	156	17.94	4.86	Yes	Yes	PBP1	*Helicoverpa armigera*	XP_021192649	129	1e−34	39
Gene.113597	AdisOBP40	MH900324	166	19.09	8.61	Yes	Yes	OBP38	*Dendrolimus punctatus*	ARO70197	157	7e−46	63
Gene.77158	AdisOBP41	MH900325	141	16.29	9.12	No	Yes	OBP19	*Helicoverpa assulta*	AGC92793	115	2e−29	44
Gene.14505	AdisOBP42	MH900326	102	11.15	5.44	No	Yes	OBP23	*Spodoptera litura*	ALD65897	98.6	3e−24	49
Gene.54039	AdisOBP43	MH900327	76	–	–	–	5ʹ lose	OBP	*Helicoverpa armigera*	AEX07280	87.8	1e−20	59
Gene.58201	AdisOBP44	MH900328	76	–	–	–	5ʹ lose	OBP23	*Spodoptera litura*	ALD65897	71.6	6e−14	48
Gene.32531	AdisOBP45	MH900329	150	16.43	4.77	Yes	Yes	OBP2	*Agrotis ipsilon*	AGR39565	119	1e−31	42
Gene.5319	AdisOBP46	MH900330	70	–	–	–	5ʹ lose	OBP14	*Spodoptera exigua*	AGP03460	117	2e−32	81
Gene.86678	AdisOBP47	MH900331	120	–	–	–	5ʹ lose	OBP13	*Sesamia inferens*	AGS36753	137	8e−39	53
Gene.141496	AdisOBP48	MH900332	106	12.10	6.95	No	Yes	OBP39	*Dendrolimus punctatus*	ARO70198	183	4e−57	82
Gene.142856	AdisOBP49	MH900333	157	17.96	9.74	Yes	Yes	OBP18	*Dendrolimus punctatus*	ARO70177	119	3e−31	51
Gene.17592	AdisOBP50	MH900334	144	16.21	4.44	Yes	Yes	OBP9	*Helicoverpa armigera*	AEB54592	163	5e−49	54
Gene.54647	AdisOBP51	MH900335	84	–	–	–	5ʹ lose	OBP39	*Dendrolimus punctatus*	ARO70198	140	1e−40	86
Gene.76032	AdisOBP52	MH900336	105	11.60	4.71	No	Yes	OBP	*Spodoptera litura*	ALD65897	111	4e−29	52
Gene.111996	AdisOBP53	MH900337	105	12.28	8.21	No	Yes	OBP	*Operophtera brumata*	KOB73304	194	1e−61	88
Gene.158529	AdisOBP54	MH900338	131	14.34	4.86	No	Yes	OBP11	*Spodoptera exigua*	AGP03457	226	3e−74	79

Genes beginning with the lowercase letter “c” came from the identification of antenna transcriptome, and genes beginning with “Gene” came from testis and ovary transcriptome identification.

**Figure 3:**
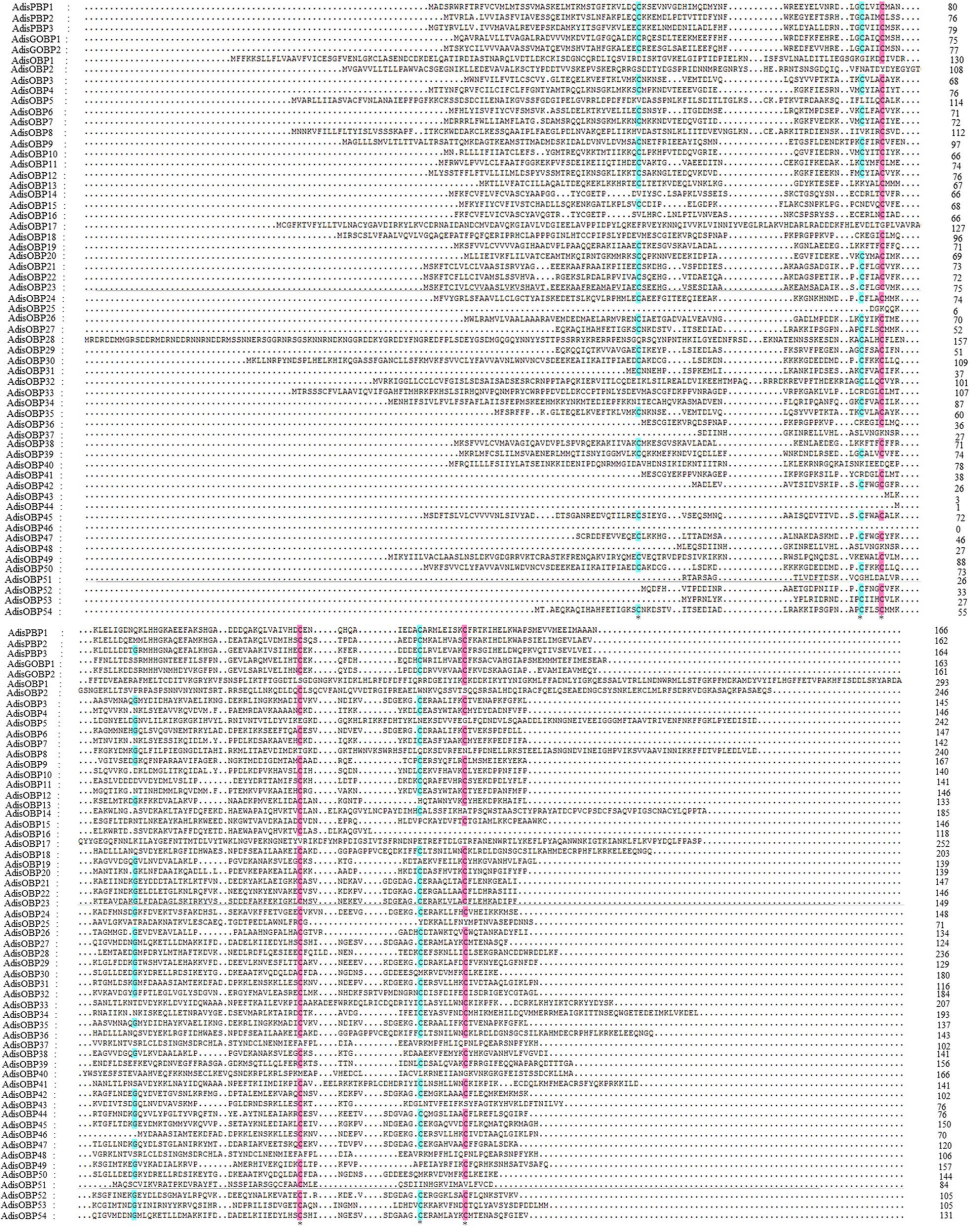
Sequence alignments of *Athetis dissimilis* OBPs. The six conserved cysteine residues are indicated with the asterisks under the sequence.

**Figure 4:**
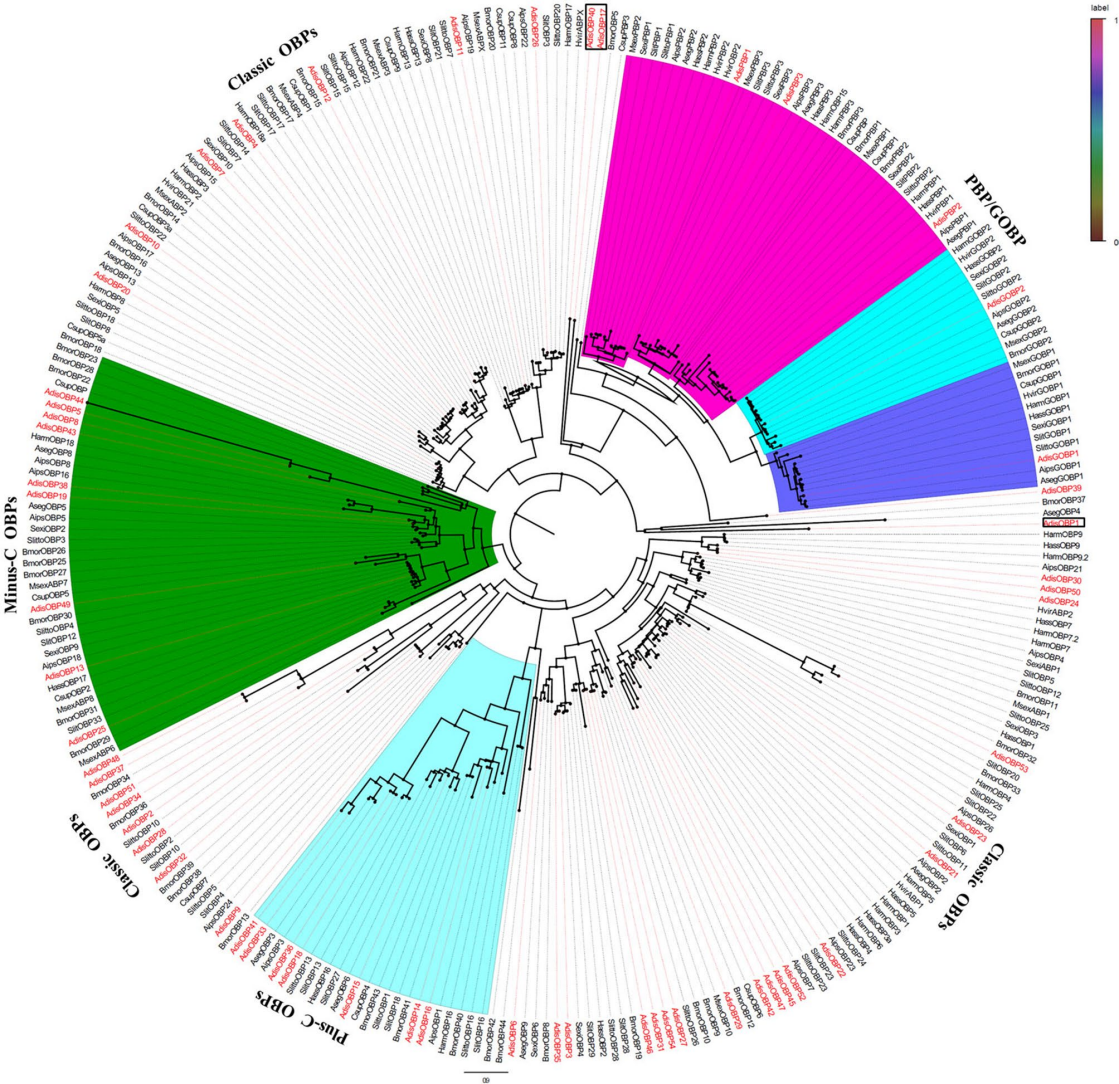
Phylogenetic relationships of candidate OBP proteins (including 5 OBPs identified in a previous study) from *Athetis dissimilis* and 33 Lepidoptera species.

**Figure 5:**
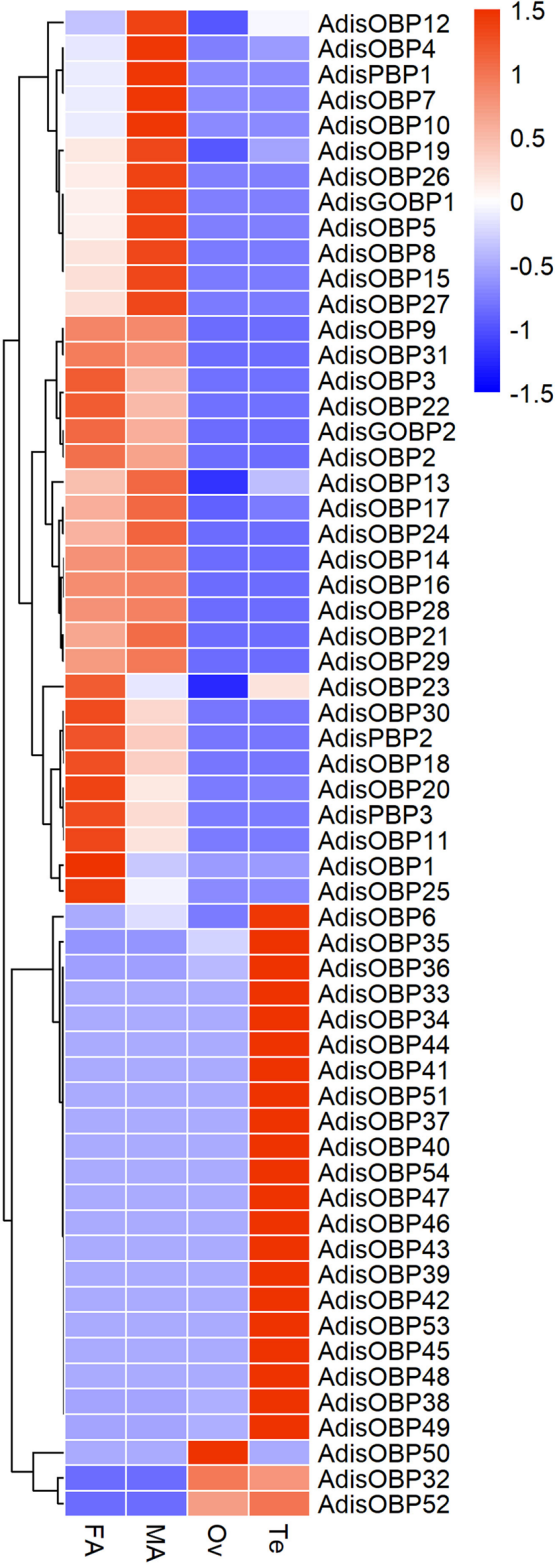
Heat map showing the abundance of unigenes encoding OBPs (including 5 OBPs identified in a previous study) in the *Athetis dissimilis* different tissues transcriptomes presented as normalized reads in reads per kilobase per million mapped reads (RPKM). In the figure each column represents 1 samples; each line represents 1 OBP gene. The color depth represents the number of reads contained in OBPs; red means more; blue means less. *FA* female antennae, *MA* male antennae, *Ov* ovaries, *Te* testis.

Multiple sequence alignments of the *A. dissimilis* OBPs revealed the presence of expected conserved cysteines (Fig. 3). The phylogenetic tree of *A. dissimilis* and other lepidopteran OBPs constructed using the neighbor-joining method, indicated five clades that contained four possible subclass OBPs (Fig. 4). In addition, the tree showed low levels of clustering highlighting the diversity of the lepidopteran OBPs. Five *Adis*OBPs (*Adis*PBP1-3, GOBP1-2) belonged to PBP/GOBP. A total of 30 OBPs (*Adis*OBP2-3, 9, 11, 20–24, 26–32, 34–35, 37, 39, 42, 45–48, 50–54) were ‘Classic’ OBPs that contained six positionally-conserved cysteine residues. Seven OBPs (*Adis*OBP14-16, 18, 33, 36 and 41) belonged to ‘Plus-C’ subclass OBP genes with more cysteines in addition to those of the conserved motif. Nine OBPs belonged to ‘Minus-C’ subclass OBP genes with only four cysteines. Interestingly, *Adis*OBP1, *Adis*OBP17 and *Adis*OBP40 did not belong to any of the four subclass OBPs (Fig. 4). However, according to BLAST results these three genes were homologous with OBP genes of *Bombyx mori*, *Spodoptera exigua* and *Dendrolimus punctatus* (Table 4). The transcription abundance of *A. dissimilis* OBPs in antennae of female and males, ovary and testis are profiled in Fig. 5.

### Expression of the OBPs in the antennae, ovaries and testis of *A. dissimilis*

**Figure 6:**
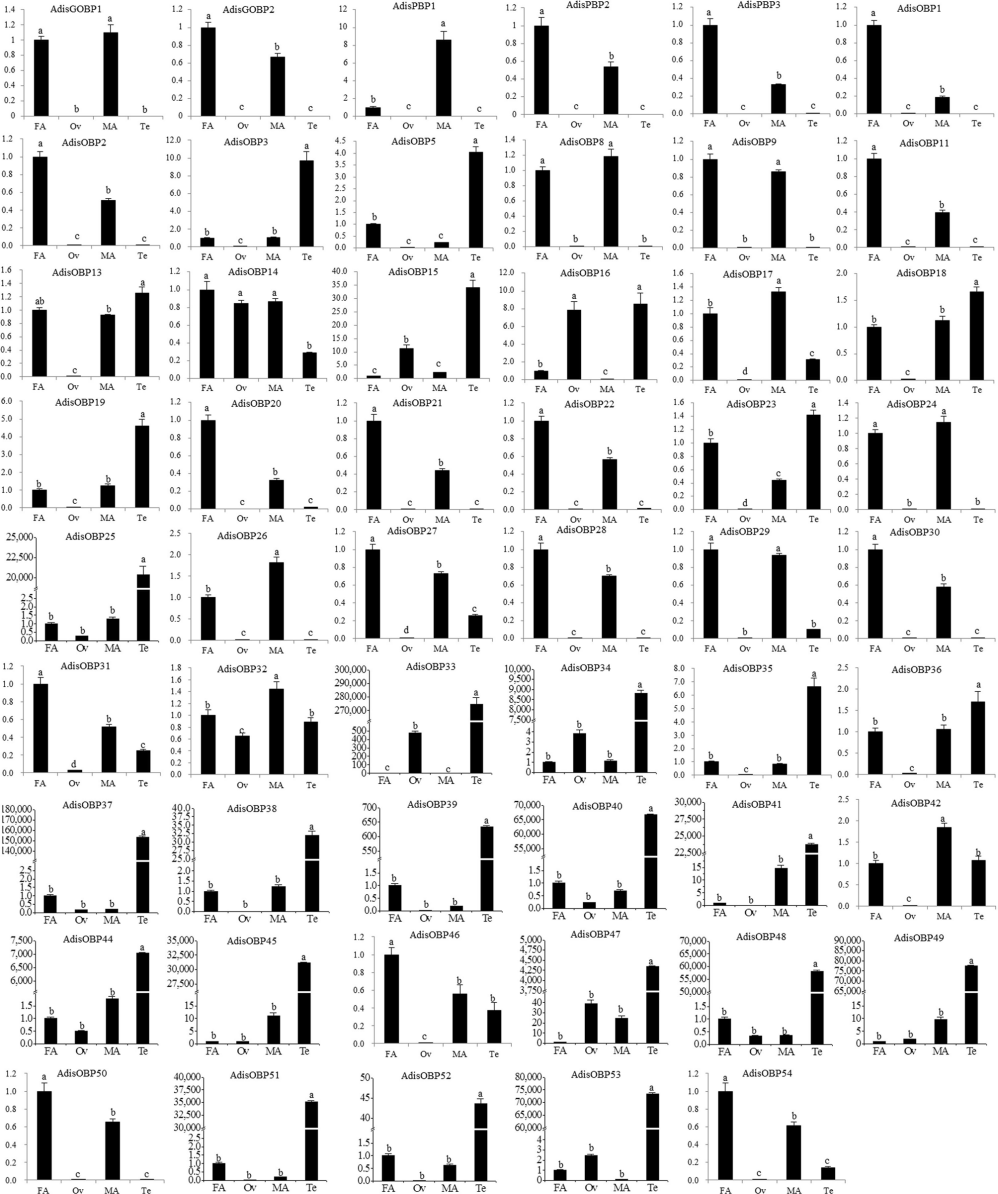
Expression profiles of the candidate OBPs in different tissues of *Athetis dissimilis. FA* female antennae, *MA* male antennae, *Ov* ovaries, *Te* testis. The standard errors are represented by the error bars; different lowercase letters (**a–c**) above the bars denote significant differences at *p* ˂ 0.05.

Next, we measured the relative expression levels of the identified OBPs in different tissues of *A. dissimilis* via fluorescence qRT-PCR (Fig. 6). A total of 23 OBPs (*AdisGOBP1-2*, *PBP1-3*, *OBP1-2*, *8–9*, *11*, *17*, *20–22*, *24*, *26–31*, *50* and *54*) were highly expressed in the antennae compared to the reproductive organs, including three OBPs (*AdisPBP1*, *OBP17* and *OBP26*) that exhibited male-biased expression, 15 OBPs (*AdisGOBP2*, *PBP2-3*, *OBP1-2*, *11*, *20–22*, *27–28*, *30–31*, *50* and *54*) that exhibited female-biased expression, and five OBPs (*Adis GOBP1*, *OBP8-9*, *24* and *29*) that showed comparable expression in the male and female antennae of *A. dissimilis*.

A total of 24 OBPs (*AdisOBP3*, *5*, *15*, *18–19*, *23*, *25*, *33–41*, *44–45*, *47–49* and *51–53*) were highly expressed in the testis of *A. dissimilis* compared to other tissues. The expression of the OBPs was low in the ovaries of *A. dissimilis.*

## Discussion

In this study, we identified 31 novel OBPs through the analysis of *A. dissimilis* antennal transcriptomes, except for 5 *AdisOBP* genes identified in a previous study^32^. The number of OBPs in *A. dissimilis* antennae were similar to those in the antennal transcriptomes of *S. litura* (33)^17^ and *S. littoralis* (36)^33^ but more abundant than *S. exigua* (11)^34^, *M. sexta* (18)^35^ and *H. armigera* (26)^36^. We additionally sequenced the transcriptomes of *A. dissimilis* ovaries and testis. The alignments against the Nr database showed that 56.87% of the *A. dissimilis* unigenes were comparable to *H. armigera* sequences. A total of 23 OBPs were identified in the transcriptomes of *A. dissimilis* reproduction organs.

Based on sequence alignments and the cluster analysis of the phylogenetic trees, five PBP/GOBP genes, 35 Classic genes, 7 Plus-C genes and 9 Minus-C genes were obtained from the *A. dissimilis* antennal library. These results were similar to the classifications of most insect OBPs^17,27,37^. Interestingly, *Adis*OBP1, *Adis*OBP17 and *Adis*OBP40 could not be clustered into any subfamilies, and multiple sequence alignments of all AdisOBP genes revealed that the three OBPs contain no conserved cysteines. The phylogenetic tree supports a highly dynamic evolutionary process for the lepidopteran OBP family and a high degree of OBP sequence divergence. The diversification of OBPs might be the result of multiple and late independent gene duplications. In addition, they might be derived from a common ancestor and later diverged into different subfamilies by different selection pressures, which has been evidenced by evolutionary selection analysis in several insect species^38–40^.

OBPs are expressed specifically in the antennae and other parts associated with olfactory organs^15,19,31,41–43^. Our comprehensive expression analysis revealed that 23 *AdisOBPs* were found to be restricted to the antenna. It is worth noting that only 3 *AdisOBPs* had male-biased expression pattern in the antennae, suggesting that females require more abundant OBPs for spawning. It is interesting to note 24 *AdisOBPs* showed significant expression in the testis of *A. dissimilis* compared to other tissues, but the expression of *AdisOBPs* in the ovaries was low. The expression of OBPs in reproduction has also been reported in some literature^44–46^. It was previously speculated that OBPs expressed in the testis deliver compounds to the females during mating^26,27^. Hence, it is understandable to presume that such stable proteins could be used in the testis of insect where there is need for transportation of hydrophobic molecules in aqueous media or protection of chemicals from degradation, as well as to assure a gradual release of semiochemicals in the environment. So these proteins have been named for ‘‘encapsulins”, to imply the common role of encapsulating small ligands^47^. qRT-PCR was conducted on 53 candidate genes, and the expression level of most genes were consistent with the variation of RPKM values.

Like the OBP families of insect antennae, insect testes contain a large number of OBP genes. The functions of these genes is unclear, and they need us to further study. Our results provide a reference for the study of these genes.

## Materials and methods

### Insect rearing and sample preparation

The *A. dissimilis* strain was collected from Luoyang (province of Henan, China) corn fields (112° 26′ E, 34° 43′ N) in 2014 and maintained at the Henan Science and Technology University. Colonies were reared on an artificial diet at 25 ± 1 °C, 80 ± 5% relative humidity and a 16-h/8-h light/dark cycle.

Based on preliminary data, we found that the *A. dissimilis* sperm and eggs began to mature 3 days after emergence. We respectively collected the ovaries and testes of 3-day old virgin females and male adults (n = 40 per treatment) from three biological replications. Dissections were performed in sterile PBS-DEPC and immediately frozen in liquid nitrogen until RNA isolation.

### cDNA library preparation and sequencing

Total RNA from the *A. dissimilis* ovaries and testis tissues were extracted using RNAiso Plus kit (TaKaRa, Dalian, China) and treated with DNase I (TaKaRa, Dalian, China) as per the manufacturer’s protocols. RNA was assessed through 1% agarose gel electrophoresis and Nanodrop 2000 (Thermo Scientific, Waltham, MA, USA), Qubit 2.0 (Life Technologies, Carlsbad, CA, USA) and Agilent 2100 (Agilent, Santa Clara, CA, USA) analysis.

Following the TruSeq RNA Sample Preparation Guide v2 (Illumina, San Diego, CA, USA), mRNA was enriched using magnetic beads crosslinked with Oligo (dT). Enriched RNA was then fragmented using fragmentation buffer and first-strand cDNA synthesis was used to produce small mRNA fragments, random primers, reverse transcriptase, and second-strand cDNA synthesis through the addition of dNTPs, DNA polymerase I, and RNase H. Double-stranded cDNA was purified with AMPure XP beads (Beckman Coulter, Brea, CA, USA) and treated to repair ends, remove poly(-A) tails, and link sequencing adapters. Fragment sizes were selected using AMPure XP beads and cDNA libraries were constructed through PCR amplification (Veriti 96-Well Thermal Cycle, Applied Biosystems, Foster City, USA). The concentration and insert size of the cDNA libraries were detected using Qubit 2.0 and Agilent 2100 and quantified via q-PCR (CFX-96, Bio-Rad, Hercules, CA, USA).

Finally, sequencing was performed using the Illumina HiSeq 4000 platform to generate 150-bp paired-end reads. Sequencing analyses were performed by the Genomics Services of the Beijing Biomarker Technologies Co., Ltd. (Beijing, China). Raw data processing and base calling were performed using Illumina software.

### Assembly and functional annotation

Raw data (raw reads) in the FASTQ format were first modified into clean data (clean reads) through Perl scripts. This was performed through the removal of reads containing adapter sequences, > 10% unknown nucleotides and quality values ≤ 20. The Q20, Q30, and GC content were then calculated using high-quality data.

Transcriptomes were assembled using Trinity (version trinityrnaseq_r20131110) with default settings, except for min_kmer_cov set to 2^48^. Unigene functions were annotated based on NCBI non-redundant protein sequences (NR, NCBI blast 2.2.28+, e-value = 1e−5), NCBI nucleotide sequences (NT, NCBI blast 2.2.28+, e-value = 1e−5), Protein family (Pfam, HMMER 3.0 package, hmmscan, e-value = 0.01), eukaryotic Ortholog Groups (KOG, NCBI blast 2.2.28+, e-value = 1e−3), SwissProt (NCBI blast 2.2.28+, e-value = 1e−5), the Kyoto Encyclopedia of Genes and Genomes (KEGG; KEGG Automatic Annotation Server [KASS], e-value = 1e−10) and Gene Ontology (GO, Blast2GO v2.5, e-value = 1e−6). Coding sequences (CDS) were predicted through aligning transcriptome sequences to the Nr and Swiss-Prot database or using estscan 3.0.3^49^. The read count for each gene was obtained by mapping clean reads to the assembled transcriptome using RSEM (bowtie2 parameters: mismatch 0). The final read count was calculated as Fragments Per Kilobase of transcript per Million mapped reads (FPKM)^50^.

### Sequence and phylogenetic analysis

Sequence similarities were assessed using the NCBI-Blast network server (http://blast.ncbi.nlm.nih.gov/). The signal peptides of OBPs were predicted using SignalP 4.1 (http://www.cbs.dtu.dk/services/SignalP/)^51^. Multiple sequence alignments were assessed using DNAMAN 6.0. Sequence alignments of the candidate OBPs were performed using ClustalX 2.1^52^ and used to construct phylogenetic trees with PhyML in Seaview v.4 based on the Jones–Taylor–Thormton (JTT) model with nearest-neighbor interchanges. Trees were viewed and edited using FigTree v.1.3.1. Amino acid sequences of OBPs in phylogenetice tree were listed in Supplementary File 1.

### Expression analysis through quantitative real-time polymerase chain reaction

Male antennae (100), female antennae (100), ovaries (80) and testes (150) tissue from adults at 3 post-eclosion were excised and frozen in liquid nitrogen. Total RNA was extracted using RNAiso Plus kits (TaKaRa, Dalian, China) and isolated RNA was transcribed to first-strand cDNA using PrimeScript RT reagent with gDNA Eraser (TaKaRa, Dalian, China) following the manufacturer’s protocols. Real-time quantitative PCR (RT-qPCR) was performed with SYBR^®^ Premix Ex Taq II (TaKaRa). The *A. dissimilis* GAPDH gene was used as an endogenous control to correct for sample-to-sample variations. A 200 ng/μL cDNA sample was used for per tissue. Primers were designed using Primer Premier 5.0 software and are listed in Supplementary File 2. RT-qPCR reactions contained: 10 μL of SYBR Premix Ex Taq II, 20 ng of cDNA template, 0.2 μM of each primer and nuclease-free water. The cycling conditions were 1 cycle of 95 °C for 5 min, followed by 40 cycles of 95 °C for 5 s and 55 °C for 30 s. Melt curve conditions were 95 °C for 10 s and 65 °C for 30 s. No-template controls (NTC) were included to detect possible contamination. Three biological replicates were analyzed and the relative expression of the OBP genes was measured using the 2^−∆∆*C*T^ method^53^. Expression was calculated relative to levels in the female antennae, which were arbitrarily set to 1. Differences in the expression of *Adis*OBP genes between the different tissues were compared using a one-way nested analysis of variance (ANOVA), followed by a Tukey’s honestly significance difference (HSD) test using SPSS (SPSS Institute 17.0, SPSS Inc, Chicago, IL, USA).

## Supplementary Information

Supplementary Legends.

Supplementary File 1.

Supplementary File 2.
