# Supplementary material for: Identification and comparative expression analysis of odorant-binding proteins in the reproductive system and antennae of Athetis dissimilis
Source: Sci Rep. 2021 Jul 6;11:13941. doi: 10.1038/s41598-021-93423-1 (PMC8260659; doi:10.1038/s41598-021-93423-1)
Supplement: Supplementary file 1 — Supplementary Legends. [file 41598_2021_93423_MOESM1_ESM.docx]

**Compared to odorant-binding proteins in the reproductive system and antennae of** ***Athetis dissimilis* using transcriptome analysis**

Yue-Qin Song, Zhi-Yu Song, Jun-Feng Dong, Qi-Hui Lv, Qing-Xiao Chen & Hui-Zhong Sun ^*^

College of Horticulture and Plant Protection, Henan University of Science and Technology, Luoyang 471000, China

**Table 1 Primers used for expression analysis by qRT-PCR in this study.**

| Primer name | Sequence（5’-3’） |
| --- | --- |
| AdisGAPDH-F | CTGCTCATTTAGAGGGTGGTGC |
| AdisGAPDH-R | TCTTCTGGGTAGCGGTGGTAG |
| AdisGOBP1-F | ATGACTTCAAGTTCGAGCACCGCGA |
| AdisGOBP1-R | CTTCTCGCACGTGTGGATCAGC |
| AdisGOBP2-F | GGAAGAGTCAGGGTTATCAGCG |
| AdisGOBP2-R | AGGACCTCACCGTTAGGGAAGC |
| AdisPBP1-F | CAACTTCTGGCGTGAGGAATACGAG |
| AdisPBP1-R | TGGACTATCGCCACCAGCTGCTTGG |
| AdisPBP2-F | GGAAGGAGGGCTACGAGTTCACAAG |
| AdisPBP2-R | GGTCGACTGCGAGCAGCTGTGGAT |
| AdisPBP3-F | GCTGGACCTGTTGGATGATAC |
| AdisPBP3-R | CTTCTTCTCGCATTCGTGGATTA |
| AdisOBP1-F | CGAAGGAAGCGGTAAAGGTAT |
| AdisOBP1-R | TTGATAAGAGGACTGTTGGAGAAC |
| AdisOBP2-F | TACTCCTCACGCTCCTACCT |
| AdisOBP2-R | AGCAACAGCCACTTCATCTTC |
| AdisOBP3-F | GCAGTTGTGTTTACGGTCTCA |
| AdisOBP3-R | CATTTGGTGGCAGTCTTTGTG |
| AdisOBP4-F | CTTACGCGATGACAAGACAACAAC |
| AdisOBP4-R | CCGCTGGAAACATGACATCTACTTGT |
| AdisOBP5-F | CGACCACACTTACAAACTGAATGA |
| AdisOBP5-R | TGAACATACCGCCTCCAATCT |
| AdisOBP6-F | AGACAGAAGACAATGCCTGACAGTG |
| AdisOBP6-R | CTGACCTCAACATCATTCACGCTT |
| AdisOBP7-F | GATGTGACTGAAGATCAAGTGGG |
| AdisOBP7-R | ATCCGGTGGATACATGAGGTC |
| AdisOBP8-F | TCTAACTGCTCATATCCGAAAGATG |
| AdisOBP8-R | CGAACGAATGTCTCCACGATT |
| AdisOBP9-F | GAACGGAACGGAAAGACCAT |
| AdisOBP9-R | ACATCAGGCATCTCAGGAATTG |
| AdisOBP10-F | TCCTGTTACGGATGATCAAGTTG |
| AdisOBP10-R | GGCTATGAATGCACAAAGATACAGC |
| AdisOBP11-F | GACGACGATGTTGTGGACTAC |
| AdisOBP11-R | ATCTGTGTACTTCAAATGCTCTCTG |
| AdisOBP12-F | TCAGCGAAGAATGGCTTAACAGAAG |
| AdisOBP12-R | TAACAGGAACCTTCATTTCTGTCG |
| AdisOBP13-F | ACGGAGACCAAGGTTGATGA |
| AdisOBP13-R | GCAGAGGGCATACTTCTTTAGC |
| AdisOBP14-F | CACCAGATGTGATCTACAGTTGTC |
| AdisOBP14-R | CAGCCTGTCGCACTCATTAG |
| AdisOBP15-F | GTTGCTGTGGACAAGGCTAT |
| AdisOBP15-R | CCGCTTCTGGACATTTCTTCA |
| AdisOBP16-F | CCAAGGAACTAGGACTTACTGTG |
| AdisOBP16-R | CTCGCACTCACTGGAATACC |
| AdisOBP17-F | ACGGTGCTCAATGCTTGTTAT |
| AdisOBP17-R | CCAATACTGCTATGCCCTTCTG |
| AdisOBP18-F | AAGGATTCCGAGGCACTGTT |
| AdisOBP18-R | CCGCAACTCTCCATAACTTCATC |
| AdisOBP19-F | GCAGAGGATGAAGGTCTAAAGAAAT |
| AdisOBP19-R | TTATCAACGCCAGGAGGTAGT |
| AdisOBP20-F | ATCGTGTCAACCAAAGAATAATGTC |
| AdisOBP20-R | GCCATGTAGCACTTCACTTCT |
| AdisOBP21-F | CAGCCATCAAGCCGATCATAG |
| AdisOBP21-R | TTGTAGACACAGCCGAGGAA |
| AdisOBP22-F | CATAGTGGCGTGCTCTCAAG |
| AdisOBP22-R | TGAAGACACAGGCAATGAAACA |
| AdisOBP23-F | GTCTTGCTTCTTAGGTTGTGTCAT |
| AdisOBP23-R | GTCGTCATCGCTGCTTACATAC |
| AdisOBP24-F | AGAAGACCGTTTCATTCGCTAAG |
| AdisOBP24-R | GACACATTCCTCTCCAACAGTT |
| AdisOBP25-F | TGATGCTAAGAACGCTACTAAGGT |
| AdisOBP25-R | GTTGGTTGGCATGTAGTTGAAGA |
| AdisOBP26-F | GTGGAGATGGACGAGGACAT |
| AdisOBP26-R | GCACTTGATGTAGCACTTGAGC |
| AdisOBP27-F | GAAGCAGATTGGCGTTATGGA |
| AdisOBP27-R | CCGTTAATATGTGAGCAGGAATGA |
| AdisOBP28-F | GAGAGATCGGGAGGGAGAAAT |
| AdisOBP28-R | GTCGCTTCCATATTCGTCACTT |
| AdisOBP29-F | TGTTGAATCATTCCTTACCACTTGT |
| AdisOBP29-R | CCGAGTTGTTCGTAGTTCTTGAC |
| AdisOBP30-F | AGTGGTGGCGGTTAATTTATGG |
| AdisOBP30-R | TAGCACAGTCCTCAGCGATAG |
| AdisOBP31-F | GCAGCAGCCTCAATAGCAAT |
| AdisOBP31-R | CATCCTTCACTGGTTCATCGTT |
| AdisOBP32-F | AATTGGTGGACTCCTGTGTTG |
| AdisOBP32-R | GTTCTATCTTCTGTGGTGCTGTG |
| AdisOBP33-F | GTATTGGCTGCTGTTATTCAGGTTA |
| AdisOBP33-R | TGCCGAATGGACAACGAATG |
| AdisOBP34-F | GGTGAAGATAGTGAGGTGATGG |
| AdisOBP34-R | GCATACAATCGTTGAACACAGAC |
| AdisOBP35-F | CCACAAAGACTGCCACCAAA |
| AdisOBP35-R | GTCATACATTCCTTGAGCGTTCA |
| AdisOBP36-F | AACTACGAGACTTAGATGGCAATAG |
| AdisOBP36-R | GCTTCAAGAAGTGAGGTCTACATT |
| AdisOBP37-F | CTCTGGACATCGCTGAGGAG |
| AdisOBP37-R | GGATTAGACCTGGCTTCTTGTG |
| AdisOBP38-F | TTAGCCGACGCACTGAAAGA |
| AdisOBP38-R | TTGTCAACGCCAGGAGGTAG |
| AdisOBP39-F | CAATTATATTGGTGGCATGGTGTT |
| AdisOBP39-R | GTCTTCGCTTCTCAGGTCATT |
| AdisOBP40-F | TAAGGAGGTTCAATCTAACGACAAG |
| AdisOBP40-R | AACGCACGCAATAAGACAATC |
| AdisOBP41-F | CAGTTGAAGAGTTACGGAAGAAGAC |
| AdisOBP41-R | GCACGCCTCCATGAACATTT |
| AdisOBP42-F | AAGTGGCAAGGCAATGTAATTC |
| AdisOBP42-R | GCTCATCTTCATTTCTTTCATCTGT |
| AdisOBP44-F | ATGTTAGACAGTTCACGAATTACGA |
| AdisOBP44-R | CGCTTACGGTTTCTTCCTTTACT |
| AdisOBP45-F | TTGTCTTGTGTGTTGTGGTTGT |
| AdisOBP45-R | GCTTGGTTCATACTCTGCTCAG |
| AdisOBP46-F | GCAGCAGCCTCAATAGCAAT |
| AdisOBP46-R | GCCATCCTTCACTGGTTCATC |
| AdisOBP47-F | CGGTCTTGCCAACATTAGGAA |
| AdisOBP47-R | CTTCTCGCATCCAGCATCAC |
| AdisOBP48-F | CTCTGGACATTGCTGAGGAAG |
| AdisOBP48-R | GGATTAGACCTGGCTTCTTGTG |
| AdisOBP49-F | GCTGACATTGCCCTGAAGAG |
| AdisOBP49-R | GTGGTTTGATTTATGCCGTTGAAA |
| AdisOBP50-F | TGGTGAAGTTTAGTGTTGTGTGTT |
| AdisOBP50-R | CGGCGATAGGTGTTATTGCTT |
| AdisOBP51-F | GGTCGGCAGGAACATTAGTC |
| AdisOBP51-R | GGCGAAGAGTTGGTGAAGTAT |
| AdisOBP52-F | CCACAGGTGAAAGATGAAGAACAA |
| AdisOBP52-R | CAAGAAGCAAGCCGACAGTT |
| AdisOBP53-F | GCGGTATAATGACGAATGATGGTTA |
| AdisOBP53-R | TCTCCGACATCCGAAATCAGTAT |
| AdisOBP54-F | GAAGCAGATTGGCGTTATGGA |
| AdisOBP54-R | CCGTTAATATGTGAGCAGGAATGA |
