# Supplementary material for: Identification and comparative expression analysis of odorant-binding proteins in the reproductive system and antennae of Athetis dissimilis
Source: Sci Rep. 2021 Jul 6;11:13941. doi: 10.1038/s41598-021-93423-1 (PMC8260659; doi:10.1038/s41598-021-93423-1)
Supplement: Supplementary file 2 — Supplementary File 1. [file 41598_2021_93423_MOESM2_ESM.docx]

**Compared to odorant-binding proteins in the reproductive system and antennae of** ***Athetis dissimilis* using transcriptome analysis**

Yue-Qin Song, Zhi-Yu Song, Jun-Feng Dong, Qi-Hui Lv, Qing-Xiao Chen & Hui-Zhong Sun ^*^

College of Horticulture and Plant Protection, Henan University of Science and Technology, Luoyang 471000, China

>BmorGOBP1

MWKLVVVLTVNLLQGALTDVYVMKDVTLGFGQALEQCREESQLTEEKMEEFFHFWNDDFKFEHRELGCAIQC

MSRHFNLLTDSSRMHHENTDKFIKSFPNGEILSQKMIDMIHTCEKKFDSEPDHCWRILRVAECFKDACNKSGLA

PSMELILAEFIMESEADK

>BmorGOBP2

MFSFLILVFVASVADSVIGTAEVMSHVTAHFGKTLEECREESGLSVDILDEFKHFWSDDFDVVHRELGCAIICMS

NKFSLMDDDVRMHHVNMDEYIKGFPNGQVLAEKMVKLIHNCEKQFDTETDDCTRVVKVAACFKKDSRKEGI

APEVAMIEAVIEKY

>BmorPBP1

MSIQGQIALALMVYMAVGSVDASQEVMKNLSLNFGKALDECKKEMTLTDAINEDFYNFWKEGYEIKNRETGC

AIMCLSTKLNMLDPEGNLHHGNAMEFAKKHGADETMAQQLIDIVHGCEKSTPANDDKCIWTLGVATCFKAEI

HKLNWAPSMDVAVGEILAEV

>BmorPBP2

MKLQVVLVVLTVEMVCGSRDVMTNLSIQFAKPLEACKKEMGLTETVLKDFYNFWIEDYEFTDRNTGCAILCMS

KKLELMDGDYNLHHGKAHEFARKHGADETMAKQLVDLIHGCSQSVATMPDECERTLKVAKCFIAEIHKLKWAP

DVELLMAEVLNEVSWKS

>BmorPBP3

MARYNIVVAVLVLGVVGARGSSEAMRHIATGFIRVLDECKQELGLTDHILTDMYHFWKLDYSMMTRETGCAIIC

MSKKLDLIDGDGKLHHGNAQAYALKHGAATEVAAKLVEVIHGCEKLHESIDDQCSRVLEVAKCFRTGVHELHW

APKLDVIVGEVMTEI

>BmorOBP5

MKQRLRVLLLRFCILQTVLSESGVDVVKNLSLSFARFFLECDEERHFQPEVRLKVMTFWYSESSTWDRDVGCAF

LCIFKKMEIDNPQDPSYRTHLELLSFANSEDNKIANQMVEIFYACGENTETDPCLWALEQVKCYKNRINQLGLTP

TF

>BmorOBP8

MLRVVVICVCFLVIAPYGINASSLDDLKMVYKNVIKECVGDYPITAADLKLIKARQIPNDDIKCVFACAYKKTGM

MTEEGMLSVEGIKDMSQKYLSDNPEQLRKSKEFAEACSSVNDQQVSDGTKGCERAALIFKCSTEKITNFGFEL

>BmorOBP9

MLRVVVICVCFLVVAPYGINAVSYEQKIKIRDQLDRAGFECFKDHKITEDDIKNLRANKPATGENVPCFIACVMKK

TGVMNDQGVIRKGPVLELAKKVLADDKDIKKLQDYIHSCSHVNSETVHDKGKGCEFAMQAYTCMSANASKFG

FNI

>BmorOBP10

MLRVVVICVCFLVIAPYGINAVSDEQKIKIREQIDKSGFECFKDHKITEDDIKNLRARKPATGENVPCFIACVMKKT

GVMNDQGVIHTEPVLQLAKKVLTDDKDIKKLQDYIHSCSHVNSKTVHDKGQGCEFAIQTYTCMSANASKFGFD

V

>BmorOBP11

MSANSFVVLAFCALAVGVNALTEEQKAEITKSSLPLIAECSKEFSVNQGDIDAAKKLGDPSGLNSCFVGCFMKKA

GIINASGLFDVAATIEKSKKYLTSEEDLKAFEKLTETCAPENDKPVSDSDKGCERAKLLLDCFVANKGSFSVFSL

>BmorOBP12

MTSFMVFFVLSVLTLKYSDALTDEQKNKIQSKFIEIGAECIVEHPISIDDINSFKNKKFPSGVNAGCFVACIFNKIGL

FDDKGNLSHNSALEKAKGIFNADEEVKNLEEFLNRCAKVNGEAVGDGVKGCERAKLAYNCLIENSLEFGFNIDF

>BmorOBP13

MLKIHVLLCFGMAILYFGSAKAVTPEESKAFEAFAKPVIEQCQKDFGMDKESFAQKNLDEIDECLIACVVEKFGIT

NDEKIDGDALKALVTKFVGNEEERNKINKIVEECTEDANKSGDGTCNTSTILFLCLLKNGKDLWGF

>BmorOBP14

MSRQQLKNSGKMLKKQCMGKNDVTEEEIGDIEKGKFIEQKNVMCYIACIYQMTQIIKNNKISYEASIKQIDLMY

PPELKESAKASAGRCKDVSKKYKDICEASYWTAKCMYEDNPKDFIFA

>BmorOBP15

MFLKNIFIECVLLYFVMLNTSFVNTMTKQQIKNSGKILKKACISKNDVTEDQISDIDKGKFIEDKNVMCYIACVYS

MSQVVKNNKFVHDAMVKQVDMMFPTEMRDAVKASIANCRGVAKNYKDICEASFWTAKCMYEFDPANFVF

A

>BmorOBP16

MRISFLFLISVTIITFDSVFAMTRAQVKKTMTIMKNQCMPKNGVTEDQVGKIEEGIFLENHNVMCYIACVYKTI

QVVKNDRLDKDLISKQIDVLYPQEIRESTKKAVGDCINLQEKYDDWCEGIFRSTKCLYEKDPANFIFP

>BmorOBP17

MTRQQLKNSGKIMKKTCMPKNDVTEEEIGQIEQGKFLEQRNVMCYIACIYTVTQVVKNNKLSYDAVIKQVDV

MFPAEMRPAVKAAAENCKDISKTFKDICEASYWTAKCMYDFDPKNFVFP

>BmorOBP18

MILIVIAKFLILISLCETMTMKQIKNTGKMMRKSCQPKNNVDDEKINPINDGVFIEENEVKCYIACIMKMANTM

KNGKLNFEAAMKQADLLLPDEMKEPTKEAIVACRKVADSYKDVCDASFHVTKCIYNHNPSVFFFP

>BmorOBP19

MTSAKTDVEIKAWFLGQAVECSKDHPVTTEELRMHKHELPDSKNAKCLMKCVFRKCNWLDSKGMYDINAAY

ASSTKDFSDDKTKQENANKLFDTCKSVNEENVGDGEEGCDRSLLLAKCLTKAAPQVSIYYS

>BmorOBP20

MAVHIFLILASYMALAAHGQLDDEIAELAAMVRENCADESSVDLNLVEKVNAGTDLATITDGKLKCYIKCTMET

AGMMSDGVVDVEAVLSLLPDSLKTKNEASLKKCDTQKGSDDCDTAYLTQICWQAANKADYFLI

>BmorOBP21

MITASLHVIFALLAFVYGGKDKPVLSEEIKEIIQTVHDECVGKTGVSEEDITNCESGIFKEDVKLKCYMFCLLEEAGL

VNDDGTVDYEMFTSLIPEEYFDRATKMIFSCKELDTPDKDKCERAFEVHKCSYEKDPDFYFLF

>BmorOBP22

MLKVFVVVVCTLGASQLCAALYTQKVAVSFPKDKTTIVVEAMKSCIAKTGANPNVIEVISSGKVSEDEKFKEFFYC

ACNDIGVVNPDGHIKVKECIELFPKETQPLVEPVIKNCDKEGVNKYDTLFKYLKCFQETSPVRVTLA

>BmorOBP23

MTSKVLLSCVVLAVLATTVLAEDSRKLVSFAPEVAKKLKVLIQECLNENGLGEDAIEVIRAGEYREDEPFQNLVYCA

YKKFGALDENNRIISQVAAASFPKDIDVVTVIESCGKEDGNTPVEQVFKYFKCFQKNSPVRMQLY

>BmorOBP25

MKSVVLICLAFAVFNCGADNVHLNEDEREKANWYTAECGVETGVSTEVINAAKIGKYSKDKAFKKFVLCFFKKS

AILNSDGTLNMVVALAKLPSGVNKSEAQSVLEQCKNKTGQDAADKAFAILQCFHKGTKTHILF

>BmorOBP26

MKSVVLICLAFAVFNCGADNVHLAETQKEKAKQYTSECVRESGVSTEAINAAKIGKYSKDKAFKNFVLCFFNKSA

IFNSDGTLNMDVALAKLPPGVNKSEAQSVLKQCKNKTGQGAADKAFEIFRCYYKGTKTHILF

>BmorOBP27

MKSVVLICLAFAVFNCGADNVHLTETQKEKAKQYTSECVKESGVSTEVINAAKTGQYSEDKAFKKFVLCFFNKSA

ILNSDGTLNMDVALAKLPPGVNKSEAQSVLEQCKDKTGQDAADKAFEIFQCYYKGTKTHILF

>BmorOBP28

MLKVFIVTFFAFQLSAIARLQANGCVAVPFPKDKTIIIVEAMKSCIAKTGANPNFIDVIRSGKVSEDEKFKEFYYCTCNDTGFVNPDGHIKVKECIELFPKETQPLVEPVIKNCDKEEGVNKYDTLFKFLKCFQETSPVRVALA

>BmorOBP29

MTGPAAAAVLLALLAAAGQATTGCKNCVILGKEERAMFRSHSDACLAQSRVEPRLLESMMNGELIDDAALRK

HVYCVLLSCKMIGKDGKLLKAAILGKLAARPAGRDVTKVLEACAEQPGASPEDVAWNIFRCGYNRKAVLFDYM

PAGGASSGNTENHP

>BmorOBP30

MRSFVILLNYGLLCCGQFMAEDYYYDIVTRDPDDLMREKENEVRALRAFQADCAEDVQVKPDLVVNLKSGDW

QTEDVSLKKWALCVLMKLGLMTAQGVFKMNEAMSKIPDMNDKIIAEKLIDDCLSLQATTPHDAAWNYIKCHH

QKDPEGNFSSLNIF

>BmorOBP31

MKTFIVFVVCVVLAQALTDEQKENLKKHRADCLSETKADEQLVNKLKTGDFKTENEPLKKYALCMLIKSQLMTK

DGKFKKDVALAKVPNAEDKLKVEKLIDACLANKGNSPHQTAWNYVKCYHEKDPKHALFL

>BmorOBP32

MYSHKYLNDFTNIPEILIILLSSVALMSYGYNTKLFSHSLGSEPSLSILYARDKKSDKVTNECLMEMYPKNLYKYPLR

IDRNDIPCIIHCVLKKFGIISNDGFINIKNYYRRVQAIHRYDPRILISDVGETCAQNINGMNLDHDVCKKAKVFND

CTQLYAISYREPEDW

>BmorOBP33

MYAHDKLSDMIADQCLNEMYPRSKRLEIEESDEPCIIFCVLKKFGIMSPTGVINLEAYRKRVQLPEQLAQRNSIN

DFGSACLESAEATQHKQDVCKKAKVFNECTHLYKILLK

>BmorOBP34

MEKMILLNVFAVVLPCVLASRTRGSSGTLVDFTDPKVQGHLDALVRMAQSCVIKVRATPKDVRAYFTNSSPVSR

SGQCFATCMLEQSDIINHGKVNRDLLVHLAGLVNGKNSRVVRKLNSVSRLCLDSISGMTDRCQLASTYNDCLNE

NMIEFAFPLDIAEEAVRKMPFHLIQPK

>BmorOBP36

MAVSEISRILTFLTIVSFIYIVYSFKPLTKDEHIERYNKMNEDIEPFRKNLTECARQVKASMADVEKFLKRIPQSNME

GKCFVACILKRNSLIKNNKLSQENLLEVNRAVYGDDSEVMSRLKTAILECSKIVEDIFEICEYASVFNDCMHMKM

EHILDKITMERRMEALGQMSSNPDEWSEEEDEMLKLVKDEL

>BmorOBP37

MFYPFRFTLLFYGLFVIYLVRAEPEKENHFTLALKKTLFSTARSCMSHVNANETDLEYLRKDPPFPDKAACIIKCLLE

KIGVVKNNKYSKMGFLTAVSPLVFTNKKKLDHYKSVSENCEKEINHDQTTECELGNEVVSCIFKYAPELHFKT

>BmorOBP38

MANLVLLLTFVLMTLSMARLKSTEAPKSKTALFNDQDNMGYEELDMEEIMSACNESFRIEYAYLESLNDSGSFP

DETDKTPKCYIRCVLEKTEILSENGVLNPATAALVFAGERNGKPMSDLEEMAVACADRHEKCKCEKAYNFVKCL

MYMEIDKYEKKN

>BmorOBP39

MVRKISALLCCFCVLGISMCDSAISTDNEQRCKNPPTAPQKIERVITLCQDEIKLSILREALDVIKEEHTMPAERKR

NKREVPFTHDEKRIAGCLLQCVYRKVKAVDGFGFPTLEGLVGLYSDGVNERGYFMAVLEASRECLMKNHDKFS

RTTPMDNGRNCDVSFDIFECISDRIGEYCGTSGL

>BmorOBP40

MSEFIQPSWRTQCNFRLNWDNRNRLSIDISHGAATTQTPVPTTKPKALRDFMVVPQSCDKTTCVFKKLNIVSD

KGVVDVKSFIKLLDKFTNSYPVWNSAKARVITTCLRKSLIAYDGGCELNNILACTFDVLSENCPLNGNNQTC

>BmorOBP41

MLTILFLLPIVVGVLSGNIPEQPRVYCGELPNTIYSCLGNPKIIQPEVSEKCNKPISECDKTRCIFKESGWAKNNVID

KKKVSDYFEQFAKDNPDWSAAVQNFKTTCLSDSLKPQGVDTNCPAYDIIHCALISFIKFASPSQWSTSEQCVYPRQYAGACPVCPERCFAPSVPNGSCNACLALLRTP

>BmorOBP42

MMGYACVFVILAVLQAISAEDPPGLPPFLKDAPEKCKSPPRVKNPNECCISEPFFKEADFIECGIEKPGSERGPPD

CSKQNCLLKKYNLLKNDETPDIEAIKSLLDKYIEKNPSFKSSVEKAKECLREDLPGPPQICLANRMTLCIGTVLLME

CPDEKWNTTDDCKAFKDHMTECQKYFPK

>BmorOBP43

MKVCVLFAIFTVAQAAKATLKPISACCNIPELGNPEPLAECSNPKLPGPCKDIQCVFEKSGFLTENKTLIKEAYKTH

LRQWAKEHEGWSVAVEKAISDCVDKDLRQYLEFPCSAYDVFTCTGIAMLKKCPNEHWTC

>BmorOBP44

MSRLVLFFTILVVLQEFIINLYFNFITEIDSCCVKKYPKLFDSEFITECYNTQRKANDKCERDMCVARKLNLLTEEDSI

NKDALLRFVEEGFKTEIDLVNAIKKKCFEEDISNIGKPEMCEVAKYKICITSRMAEDCPKWDSKGICSSAQQKVEN

FMKMLS

>HarmPBP1

MEFHRSTMMSVRLALVVAVCLFIRVDASQDVIKNLSMNFAKPLEDCKKEMDLPDSVTTDFYNFWKEGYEFTN

RQTGCAILCLSSKLELLDQELKLHHGKAQEFAKKHGADDAMAKQLVDLIHGCAQSTPDVADDPCMKTLNVAKC

FKAKIHELNWAPSMELVVGEVLAEV

>HarmPBP2

MAASRWLFARAFCLVLMMGSAMSSKELLTKMTGGFTKVVDACKTELSVGDHIMQDMYNFWREEYQLVNRD

LGCMIMCMTAKLDLIGDDQKMHHGKAEEFAKSHGADDALAKQLVGLIHGCETQHQAIEDHCSRALEIAKCFRT

KIHELKWAPSMEVIMEEIMTAA

>HarmPBP3

MGSRHVFFALVVLAVSVRKAEPSKDAMQYITSGFVKVLEECKHELNLNEQILADLFHFWKLEYSLLGRDTGCAII

CMSKKLDLLDANGRMHHGNAAEFAKKHGAGDEVASKIVTIIHECEKKHEQDGDECLRVLEVAKCFRTGIHELN

WQPKVEVIVSEVLTEI

>HarmGOBP1

MPGVLRALLVLAAAAPLLADINVMKDVTLGFGQALDKCREESQLTEEKMEEFFHFWRDDFKFEHRELGCAIQC

MSRHFNLLTDSSRMHHDNTEKFIQSFPNGEVLARQMVELIHSCEKQFDHEDDHCWRILHVAECFKGSCVQRG

IAPSMELMMTEFIMEAEAR

>HarmGOBP2

MTSKSCLLLVAMATLTASVMGTAEVMSHVTAHFGKALEECREESGLSAEVLEEFQHFWREDFEVVHRELGCAII

CMSNKFSLLQDDSRMHHVNMHDYVKSFPNGHVLSEKLVELIHNCEKKYDTMTDDCDRVVKVAACFKVDAKA

AGIAPEVAMIEAVMEKY

>HarmOBP1

MSKFTFFVLCVVAVSLSKVYASDEDKAKLHEALKPLVEECMKDHEVSLDDLKAAKEAKSADGVKPCFLACVYKK

AEVLNDKGEFDADHALEKLKEFVSDEDVLAKVAEVGNTCKAVNDKAVSDGDAGCERAALLTACFLEHKAEILV

>HarmOBP2

MMDRKRLCLLIIAMFLAQGSDAMSRQQLKNSGKMLKKNCMNKNQVTEDQIGSIDKGKFVEDKKVMCYIACI

FEMTNVVKNNKLNYDASIKQIDLMYPPDLKESAKAAVEKCKDVQKKYKDICEASYWTAKCMYDFKPEDFIFA

>HarmOBP3

MSKFTCFVLCVLAVSLGEVRSNALEKAAIRAAVYPLIVDCAKEHAVTLEQLKAAKASHSAEGINPCFQSCVYKKT

GIFNDNGEYDVANAKTKLQKFVTDEDEYARIAEVGKTCASVNDKSVSDGAAGCERAALLTACFLEHRAQIII

>HarmOBP4

MSKLTCVVFAAVAVVFSNVNADDETRASFRQVLGPLVMECRNEFGITEDDLKKAQQERSPDALKPCFIACVFKK

FGIITSAGKYDSDASISRIKDVVKNDDLLAKLKSVGEKCNSVNDASVSDGDAGCERAALLAKCFIENKSELSI

>HarmOBP5

MSKFTCLVLCVVAASLSQAYASEEEKAAFREAIKPIVEECSKEHGVSHDELKSAKDNQNADNIKPCFLGCVYKKAE

VFNSKGEYDVDKALEKLKKFVSNDEAYAKFAEVGKKCASVNDKAVSDGDAGCERGALLTACFLEHKAEVPL

>HarmOBP6

MSKFTCLLLCVVAVSLSKVHATEEEKEAIRAAVRPIMQECGKEHGVTLDDLKAAKAAHSADGIKPCFQSCVYKKA

GIFNDNGEYDIANAKTKLQKFVTNDEEYARIAEVGKMCASVNDKPVTDGAAGCDRAALLTACFLEHRAQIII

>HarmOBP7

MFRFGVLSFVVLLFCMESSYALSSEEELSIKEALHPFVVECAEEYGMTEEMFEEAKKKGSAEDIDPCFMSCFLKKT

GFFDDSGKFDAEKSISFAKEHITSESAIKFLEAGAGECVKINDEDVSDGENGCDRAKLLFDCLTELKKKMSE

>HarmOBP7.2

MSRFGVLSFVVLVFCMENIYALSSEEELSIKEALHPFVVECAEEYGMTEEMFEEAKKKGSAEDIDPCFMSCFLKKT

GFFDDAGKFDAEKSISFAKEHITSETAIKFLEAGAGECVKINDEDVSDGDKGCDRAKLLFDCLTDLKKKMSE

>HarmOBP8

MLLIEIVKFLTLVAMCEAMTMKQIRNTGKMMRKSCQPKNNVADEQIDPIAEGVFNEDKEVKCYMACIMKMA

NTIKNGKLNYEAAIKQADLLLPDDIKEPAKEAITACRKVADAYKDICDASFHITKCIYTQNPGIFYFP

>HarmOBP9

MCKFSVLFLYSAVMAVNIWSASCISEEDKAAIITAIAPLAQNCGSECGLDNDDFEKYKEDGSDMDPCFKACLMT

QMGVLDKEGKYDGKGLHKAMEEADYPGDKDDAQKFLDELDRCFDAKGDNSGSDEEAKMKRADVLFRCMQ

DMKEK

>HarmOBP9.2

MCKCSVVFLYLAVMAINIWRASCLSEEDKAAIITAIAPLAQNCGSECGLDNDDFEKYKEDGSDMDPCFKACLMT

QMGVLDKEGKYDGKGLHKAMEEADYPGDKDDAQKFLDELDRCFDAKGDNSGSDEEAKMKRADVLFQCMQ

DMKEN

>HarmOBP13

MFTGTLPLVVFLATFAYGGKEKPVFSDEIKEIIQTVHDECVAKTGVAEEDITNCENGIFKEDPKLKCYMFCLMEEA

SLVDDDDAVDYDMLVSLIPEEYVDRTTKMIFSCKHLDTPDKDKCQRAFEVHKCSYEKDPDLYFLF

>HarmOBP18

MKSFVVFCVLVAGAFAANVSLPPKQNEKANQIATECMKESGLKPEVLAEAKKGHISDDEHLKKFTFCFFKKAGIV

SEDGKLNTEVALAKLPPGVDKAEAEKLLETCKGKTGKDVTDTVFEIFKCYHHGTKTHILLGF

>HarmOBP15

MGSRHVFFALVVLAVSVKKEKPSKHPMPYITSRFVKVLEECQHELKLNEHILEHLFHFWKLEYSLLGKDPGCAIIC

MSTKLDLLDLYGRMHRGNAAEFAKKHAAGDEVPSKIVTIIHFCQKKHEQDGDECLQVLEVATCCRTGLHDLNW

QHQVEVIVPDVLTEI

>HarmOBP16

MFKLCVVLAFIVATCHGGTLERTSSTCGQIPRELTACLDLQPAVSPEIQEKCRRANECERLTCVFREYNLLDGAEV

NKERTAAFLDNFVKQYPSWEVAIDVAKTSCLRSSGLKPQGVFLDCPAYDIIQCVFANLVKNALPSQWSSMSQCN

HAREFAAACPICPDACFAPLVPIGTCNACSAARRSS

>HarmOBP17

MRAWSVTLVALLGALGAARAVAMDEDMAELARMVRENCAAETGADVALVERVNAGADLMPDDKLKCYIKC

TMETAGMMADGEVDIEAVLALLPPELAEHNAPSLRACGTVRGADHCDTAFRTQQCWQNANKADYFLI

>HarmOBP18a

MTRQQLKNSGKLMKKSCMPKNDVTEEEVGDIEKGKFIESRNVMCYVACIYTMTQVVKNNKLSYEAVIKQVDM

MFPAEMRDAVKAAATSCKDITKKSKDLCESAYWTAKCMYDYDAENFVFP

>HarmOBP22

MTREQIKNSGKLIKKTCMAKNDLSEDQVKDVDKGKFIEEKPFMCYIACVYKMGQTIKGNTVNHDMMIKQVE

MMFPNEMKAPMKAAIEHCRPVVKKYKDVCEVSYWTAKCIYEFDPPNFMFP

>AipsPBP1

MAPHPSVTMYVRLALVIIAGLFITVECSQEIIKNLSLQFAKPLEDCKKEMDLSDTVITDFYNFWKEGYEFTNRQFG

CAILCLSSKLELLDQDLKLHHGKAQEFAKKHGADEAMAKQLVDMIHSCTQSTPDVADDPCMKTLNVAKCFVAK

IHDLKWAPSMDLIMGEVLAEV

>AipsPBP2

MAASRWCIACLVCVLFAARSVMTSQEVVASFSKGFTNVVEHCKAEVNAGEHIMQDIYNFWREEYQLVNRDLG

CMVLCMANKLGLIGEDQKMHHAKAEEFAKSHGADEAVAKQLVAILYECETKHAAVEDECGMALEIAKCFRTK

MHELKWAPSMEVAMEEIMTAV

>AipsPBP3

MGTYNVFFAFVLMAAGVREIEPSKDAMKYITSGFVKVLEECKQELNMNDRIIADLFHYWKLDYTLLNRDTGCAI

ICMSKKLDLLDDTGRMHHGNAQEFALKHGAGEEVASKIVTIIHDCEKKFERDDDECLRVLEVAKCFRTGIHDLD

WQPKVEVIVSEVFTDM

>AipsGOBP1

MTQPGQVLVLVLLAAAALADVNVMKDVTLGFGQALDKCRQESDLTEEKMEEFFHFWRDDFKFEHRELGCAIQ

CMSRHFNLLTDSSRMHHVNTEEFIQSFPNGEVLARQMVALIHGCEKQFDHEDDHCWRILHVAECFKHACVAH

GVAPSMEMMMTEFIMEAEAR

>AipsGOBP2

MTLRCCLLLVVVAAVTRSVVGTAEVMSHVTAHFGKALEECRDESGLSAEVLEEFQHFWREDFEVVHRELGCAII

CMSNKFSLLQDDSRMHHVNMHDYVKGFPNGEVLSGKLVELIHNCEKQYDTLTDDCDRVVKVAACFKVDAKA

AGIAPEVAMIEAVMEKY

>AipsOBP1

MDISKRRSKNAFRRLLVNTWLRLVQIFTCLSAPPVVSADVTSKCQGSKYENECDKLTCVFRKAKWLDGNAVDKA

KLITYFEQFEKDHPEWAPAMQNVKTSCLGAELKTQGVFLNCPAYDVMHCVLGSFIKHATPTQWSTSASCSYPR

AYAAACPICPEDCFSAQVPFGSCNACYLPPRTP

>AipsOBP2

MSKFTCLVLCVVAASISRVHADDDANKAAFREAFKPILDECSKEHGVSNDDIDAAKKAGSADAIKPCFFGCIYKK

AEVFNAKGEYDVDSALSKLKKFVPDEAKFAKYAEIGKKCASVNEKPVTDGDAGCERGAMLTACFLENRAEMLI

>AipsOBP3

MIRSCRCLVFAAVFQVVLGQGLTGTDSGPPGFQRPQSYVPKHCFAPPPGVDLHTCCPIPQLFPDEDMESCGIQK

LTKEQYENPSPARIPCQESICLLRNANLLKQNNSIDYEKMGDFVDNWAKMDPDFTIPITNAKKVCLIEGGPPAPP

VCEPDRIFTCLTSYVLWNCKLRLDSGEGCKILKEHMDGCRPFLAGP

>AipsOBP4

MFGYQFLSFAAALICFGSSYALTSEEEANIKEAFHPFIMKCAEEYGITEEQFEEAKEKHSAEGIDPCFMSCFMKES

GFFDSAGKFDADKTKEFVDAHLTSERAITFMEAVGSECAKVNDEEVTDGDKGCDRAKLMWGCIQDLKEKMEG

SE

>AipsOBP5

MKYFVLFVALVAGIHANVTLPPEQSEKALKTASECIKETGVSKEVLAEAKKGHIADDEGLKKFTLCFFKKAGIVDN

DGKLNLETALAKLPPGVDKAEAKKVLEGCQAKSGKTPQDTAFEIYKCYHAGAKTHIALAGI

>AipsOBP7

MSKFTCVLCVVALSLSSVYVTRAHKPNLRDAWRSELDECAKEYPVTNDEIDTAVRSGDSSNLNPCFNFCVFNKT

GFFTENGEYDLKNGLIKLRKAIRDDEEYTKFEEVATECTEDKNTSCDEKAKCDSANRLSLCFLRFKDKVRI

>AipsOBP8

MYLRSTNGGVRSFPLGESAYTTKIVEICSKETGLKKQVPPEEKEIKFSQRKGLREFNDCYLAKTGVTTSDGKLNIDE

ALEKLPPGFAKPFVEHCQANIILGYIEENVNDFSTCFHQEVQNHLLSFYGFENYWVMLVLGTSFDKTRFTTLFFD

KHFDFWLAERAGFVNL

>AipsOBP12

MYSGTIFLFSFILLIVSNVTFVSSQMTREQVKNSGKLVKKTCSAKNDLTEDEVKDVDKGKFIEEKKFMCYVACVYK

MGQAVKGNSLNHDMMIRQVDMLFPADMKAPVKAAIEHCRPVAKKYKDICEASYWTAKCVYEFDPPNFMFP

>AipsOBP13

MVLIYIVKFLILVAMCEAMTMKQIRNTGKMMRKSCQPKNNVEDEKIDPIAEGIFIDEPEVKCYMACIMKMANT

LKNGKLNFDAALKQADLLLPDDIKEPAKEAIIACKKAAEGHKDICDVSFHVTKCIYNQNPGIFYFP

>AipsOBP15

MDHNRLCLLVIAMFLATGSDAMTRQQLKNSGKILKKNCMNKHQVTEDQIGTIEKGKFVEDKKVMCYIACIYEL

TSVIKNNKLNYESSLRQIDIMYPADLKESAKAAVENCKDVQKKYKDICEASFHTAKCMYDFKPEDFIFA

>AipsOBP16

MFPGSIPFISGCVHLGVSNYFRSTQSNLVVHYEDDQIVDAIYNCQDENGFDEVLSNSTNLEENFPEKEGLKKSN

DCFLKKTGFVTSDGKLNIDKTLEKLPPSFVKPIVEHCQANIALNYTTESVENFSSCYHDGILNHIFAATEVGIFPFIQ

TWKFFVPGTSFADTILILN

>AipsOBP17

MNQLLVFVLIVACVRISNGMTREQVKKTMTVIKKQCMPKNSVTEDQIGKIEQGVFNEDRNVMCYVACVYKSL

QVVKNERLDLGLISKQIDALYPPELKEPTKKAVSQCINIQDSYNDLCEAVFHSVKCLYEKDPATFIFP

>AipsOBP18

MKTLFVFAACILLAQALTDEQKEKLKKHRTECLTETKVEEALVNKLKGGDYKTESEPLKKYALCMMTKSELMTKD

GKFKKDVALAKVPNAADKPSVEKLIDACLANKGNTPHQTAWNYVKCYHEKDPKHAIFL

>AipsOBP19

MFTGTVPFVLCLVAVAFGGKDKPVFSEEIKEIIQTVHDECVANTGVAEEDITNCENGIFKEDPKLKCYMFCLMEE

ASLVDDDGTVDYDMLVSLIPDEYYERTTKMIFACKHLDTPDKDKCQRAFEVHRCSYEKDPDLYFLF

>AipsOBP21

MLKFSVVCLYFSVAAVNFWNVHCISEDEKKAFIEAMKPMVEECGSDCGLTEEDYKKHSKGEDMDPCFKKCMM

QKLGFLDEDGKYNRKQLHESISEYTGDKDEAKRVQEQLDSCFDANGDNDGDDEESQMKRVDVLFKCLKEIKE

>AipsOBP22

MSMWFRAMVVVGALAAARCGVVMDEDMAELARMVRESCVDETGADVKLVEAVNGGADLMEDDKLKCYI

KCTMETAGMMSDGEVDIEAVMALLPPEMAEHNGPALKSCGTQRGADDCDTAWKTQVCWQNANKAEYFLI

>AipsOBP23

MSKFTYLVLCFVAVSRVYANEDERAAFHEAAKPILVECSKENGVSFDKLKAAKEAGSADGIDPCFFSCVFKKTGVF

NSKGDFDLDNSLTKLKEFVSNDEDYAKVAEVGKKCESVNEKDVSDGEAGCERASLLTACFLEHRAEIPV

>AipsOBP24

MAKLLLAMILTVMTFALTMSATTKDAGTKEAIMTTTVANQDSSIDSNDVDVLAVMNVCNESFRIEMSYIQALN

ESGSFVDETDKTPKCFIRCVFENVGIVSEDGRMFNPARAAVIFAGERNGKPMDDIADMTALCAADRKETCPCD

RSYQFLRCLMSMEIERYEKS

>AipsOBP26

MSKFTCIVLFVVAASLTKVTQAVSEEEKAVAREAMAPILAECSKAEGVSDEDIEEAKKNPSVDAVNSCFIRCVMR

KTDALNEKGLFDSDAALAKIRPFVKSDEDFAKFEEIGKACMSVNDKEVSDGEAGCDRAKLLLACFLEHKAEMLY

>SlitPBP1

MANARWRFVFVVYALYLTSAVLGSQDLMVKMTKGFTRVVDDCKTELNVGDHIMQDMYNYWREDYQLINRD

MGCMLLCMAKKLDLMDDQTMHHGKTEDFAKSHGADDDVAKKLVSVIHECEQQHAGIADDCMRVLEVAKCFRTKIHELKWAPSIEVIMEEVMTAV

>SlitPBP2

MAFCPSVTMSLRVALVVAASLLVVVQASQDVMKNLAVNFAKPLDDCKKEMDLPDSVTTDFYNFWKEGYELTN

RQTGCAILCLSSKLEILDQELNLHHGRAQEFAMKHGADEAMAKQIVDMIHTCAQSTPDEAADPCMKALNVAK

CFKLKVHELNWAPSVELIVGEVLAEV

>SlitPBP3

MGSRNVFVALVVLTVGMREIEPSKDPMKYIASGFVKVLEECKHELNMNDHLIADLFHYWKLEYTLLNRDTGCAI

ICMGKKLDLLDASGRMHHGNAQEFAKKHGAGDEVASQIVQIIHDCEKKHERDDDECLRVLEVAKCFRTGIHEL

NWQPNVEVIVSEVLTEI

>SlitGOBP1

MLLLLRALPLLAAVLPLRADVNVMKDVTLGFGQALDKCRQESQLTEEKMEEFFHFWREDFKFEHRELGCAIQC

MSRHFNLLTDTSRMHHENTEQFIQSFPNGEVLARQMVELIHACEKQHDHEEDHCWRILHVAECFKQACVQR

GIAPSMEIMITEFIMEAEAR

>SlitGOBP2

MTSKCCLLLVLMAAATSSVMGTAEVMSHVTAHFGKALEECREESGLSAEVLEEFQHFWREDFEVVHRELGCAII

CMSNKFSLLQDDSRMHHVNMHDYVKSFPNGHVLSEKLVGLIHNCEKQFDSMTDDCERVVKVAACFKVDAKA

AGIAPEVAMIEAVMEKY

>SlitOBP3

MWMQALVLTLATLATLAAAAVEMDEDMAELARMVRDNCAGETGVDVALVEKVNAGAELMPDDKLKCYIKC

TMETAGMMADGEVDIEAVLALLPPSLAEHNAPALRACGTQRGADHCDTAFRTQQCWQNANKADYFLI

>SlitOBP4

MTKVLFAIVLTMITFAVVLSASTKEAMTTTMSDQVNSIDVDVLAVMDMCNDSYRIDPTYLQALNESGSFIDETD

KTPKCFIRCVFENVGIVSEDGKQFNPARAAVIFAGERNGKPMEDIADMTALCATDRQETCPCDRSYKFLRCLMS

MEIERYEKS

>SlitOBP5

MSVVRCSSLLVAIFCFVSVNAISGDEEAGIKDALRPFVQECADEFGITEEQFEEAKKKASAADIDPCFMSCFLKKA

EFFDSQGKFDVDSTMAFAKEHLTSEPAMKFVEAVGDECVKINDEDVSDGDKGCDRAKLLFECIAETKKKME

>SlitOBP6

MSKFTCLVLCVVAVSLSGVHATAEEKAAFIEAVKPYVQECSKEHGVTPEDIKSAKAAGNADGINSCFLSCVYKKA

EVITEKGEYDADKALEKLKKFVSNEDDYAKFANIGKKCASVNEKSVSDGEAGCERAALLTSCFLEHKSEISA

>SlitOBP7

MDQKRICLFVIAMFLASGSDAMSRQQLKNSGKMLKKNCMNKIGVTEDQIGSIDKGKFIEDRKVMCYIACIYELT

NVIKNNKLNYEASIKQIDLMYPPDVKESAKAAVEKCKDVQKKYKDICEASFYAAKCMYEFKPEDFIFA

>SlitOBP8

MLLTKIVKFFILVATCEAMTMKQIKNTGKMMRKTCQPKNNAEDEKIDPISDGVFIDEKEVKCYMACIMKMANT

IKNGKLNYDAAMKQADLLFPDDIKEPAKEAITACRKVADAHKDICDASFHVTKCIYNHNPGIFYFP

>SlitOBP10

MVRKISGLLCCLCVFGISFSDSAISADSESRCRNPPTAPQKIERVITLCQDEIKLSILREALDVIKEEHTMPAQRRRD

KREVPFTHDEKRIAGCLLQCVYRKVKAVDGYGFPTLEGLVGLYSDGVNERGYFMAVLEASRECLMKNHDKFSRT

VPMDNGRNCDISFDIFECISDRIGEYCGTSGL

>SlitOBP12

MKTLFVFAACILLAQALTDEQKEKLKKHRTECLTETKVDEELVNKLKGGDYKMDNEALKKYALCMMMKSELMT

KDGKFKKDVALAKVPNPADKPTVEKLIDACLANKGNTPHQTAWNYVKCYHEKDPKHAIFL

>SlitOBP13

MITSCLLVLSAVVQVLLAKQPVFESGPPEPWGPPERTSHPGQFQPRVPKRCWVPPQRINVYNCCPIPTLYPDED

MQSCGFEKLSENKPQKPVYRPEGTCKEGYCVMGKFDLLLANNSVDYVKFREYLDNWAESYPEFANAIHIAKEE

CAQDGGPEVPPICEPDKLFLCLTSTIFWNCKLRDGEGCAALQEHMNECKQYYTRVMAPTIKDFEVR

>SlitOBP15

MYSINCFIFSVILIVMFDNCFVYSMTREQIKNSGKLIKKTCSAKNDLTEDEVKDVDKGKFIEKKDFMCYIACVYKM

GQSVKGSTINHDMMLRQVDMMFPNDMKAPVKSAIEHCRPVAKNYKDLCEASYWTAKCIYDFDPANFMFP

>SlitOBP16

MYRFVILSIVLVSALADDIDIRECGRIFHPPPHGCCKANNAVKNKDMLAEELKDCFDGSGPKDPMKCEIDLCIAK

KKGFATDDGKLDIKKFEEVITKEVGSDKDLLDEIKTNCINGDLNNYGPPEFCDFMKIKHCVTLHMMNHCSEWS

DDGNCKVVKELVGKCAKVI

>SlitOBP17

MKTFRLLCCILSIFLFFDQSYGMTRQQLKNSGKLMKKSCMPKNDVTEDEVGDIEKGKFIETRNVMCYIACVYTM

SQVVKNNKLSYEAVIKQVDVMFPAEMRDAVKAAATHCKETTKKYKDLCESSYWTAKCMYDYDAQNFVFP

>SlitOBP18

MFKLCVFLALGFVACHGAPNSSPGTPNANPGTYCGVTPDNIYRCLNNPRVVTPEVSTKCGSQFTECEKMTCIFR

ELKWSKRGAIDKAKVRAYFDQYETEHPEWAQAVQHVKAFCLASELRAQGVFLNCPAYDIMQCVLASFIKHASP

SVWSTATDCAYPKAYAADCPVCPSDCYSPQIPFGSCNACYTQPRTV

>SlitOBP20

MEKILIFTFITLSGFAHARISVMYAHDKLSDLVAQQCLSEMYPKNKRIEIQESDEPCIIFCVLKKFGIISASGVINLDIY

RKRVQIAHQLDQKTSIMDYGGSCMENAEATQHKQDVCKKAKVFNDCTHLYRILLM

>SlitOBP21

MARRQRGAMFTEALPLFVILVAVTHGGKNKPVFSDEIKEIIQTVHDECVAKTGVAEEDITNCENGIFKEDAKLKC

YMFCLLEEASLVDDDDTVDYDMLVSLIPDEYYERTTKMIFACKHLDTPDKDRCQRAFEVHKCSYEKDPDLYFLF

>SlitOBP22

MSKFTCIILCVVAASLTKVSHAAVTEEEKEAFREAMAPIIAECSEEHGVSEADIKAAKESASADNIKPCFLGCVMK

KIEVLDAKGLYDAETGLGKLRKFVKDDDEFAKFEDIAKKCLKVNDESVSDGEAGCDRAKLVLGCFIEHKVEMPF

>SlitOBP23

MAKFSCLVLCVVAASLGSIHVASGESLRESLRPVIVACSQEHGVTDAEIQAAKDAGSPASIKPCFIACVFKKAGFIN

EQGQLDLETGLKNLRQFVKDDEQYKKLEEVAKKCSQVKDKAVSDGAAGCERGVLLAGCFLEHKTSIII

>SlitOBP25

MAKVTCIVLFVVGVSLSSIQADDGKNESEVEIDVNQIIDDCIEEYHIPRRLFLAAAETGSTHALTPCFWSCCFKGV

GVLNSEGQYDIDATLDLSKKIFTDHEYEKVEIIVKKCESVNGAPVSNGNIECEKSVLLADCLFDNAKKHFPNMFG

VDY

>SlitOBP27

MYKFVILCSIFVAASNADVAQTLTKRETKASLKPLSVCCDIPELADEFQLAKCSPRPPGPCEDVQCIFEVSGFLTDR

NTLNKAAYRSHLQKWEKNHPGWTDSIYKAITDCVDNDPRQHLEVPCKAYDVFTCTGIAMLKKCPDTAWKC

>SlitOBP28

MIVRFLLCLYIVEFYGAHARTDQEIKAWFFREGMDCNIEHPISPKEMLELKENKIPDTNNAKCFVACVFKKTGML

DSKGMFDAENSIAMTQKDFANDPNRLESSKKLLEACKKVNDEAVSDGEKGCERSVLLHKCFVETAPQLGIKLP

>SlitOBP29

MWNLLVVFLAICSCVYARRRSSGAEINGLTEEELKMEFTKLIMKCNKDGEVDMTELVQLQNYVVPTKQSTKCVL

ACAYKAAEVMNAKGEYDIDHAYKVAEMMKNGDEKRLVNAKKMADLCVKVNELSVSDGEKGCDRAAMIFKCT

VENAPKFGFKL

>SlitOBP33

MTCSQALALLALVAISQQATTGCKNCIMLGKEEKAMFRAHSDACVAASRVEPRLVDAMLAGELLDEPALRKHV

YCVLLKCKLISKDGKLQKAAVLGKMAARPDAKNATKVLESCADQTGDTPEDLAWNLFRCGYDKKALLFDYMPT

NVASETDNNS

>SlittoPBP1

MANARWRFVFVVYALYLTSAVLGSQDLMAKMTKGFTRVVDDCKTELNVGDHIMQDMYNYWREDYQLINRD

MGCMLLCMAKKLDLMDDQTMHHGKTEDFAKSHGADDDVAKKLVSVIHECEQQHTGIADDCMRVLEVAKCF

RTKIHELKWAPSMEVIMEEVMTAV

>SlittoPBP2

MSLRVALVVAASLLVVVQASQDVMKNLAINFAKPLDDCKKEMDLPDSVTTDFYNFWKEGYELTNRQTGCAILC

LSSKLEILDQELNLHHGRAQEFAMKHGADEAMAKQIVDMIHTCAQSTPDVAADPCMKTLNVAKCFKLKVHEL

NWAPSVELIVGEVLAEV

>SlittoPBP3

MGSRNVFVALVVLTVAMRETEPSKDPMKYIASGFVKVLEECKHELNMNDHLIADLFHYWKLEYTLLNRDTGCA

IICMGKKLDLLDANGRMHHGNAQEFAKKHGAGDEVASQIVQIIHECEKKHERDDDECLRVLEVAKCFRTGIHEL

NWQPNVEVIVSEVLTEI

>SlittoGOBP1

MLLLLALPLLAAVLPLRADVNVMKDVTLGFGQALDKCRQESQLTEEKMEEFFHFWRDDFKFEHRELGCAIQC

MSRHFNLLTDSSRMHHENTEQFIQSFPNGEVLARQMVELIHACEKQHDHEDDHCWRILHVAECFKQACVQR

GIAPSMEMMITEFIMEAEAR

>SlittoGOBP2

MATVTSSVMGTAEVMSHVTAHFGKALEECREESGLSAEVLEEFQHFWRDDFEVVHRELGCAIICMSNKFSLLQ

DDSRMHHVNMHDYVKSFPNGHVLSEKLVGLIHNCEKQFDSMTDDCERVVKVAACFKVDAKAAGIAPEVAMI

EAVMEKY

>SlittoOBP1

MFKLCVFLALGFVACHGASNSNPGTPNANPGTYCGVTPDNIYRCLNNPRVVTPEVSTKCGSQFTECEKMTCIF

RELKWSKRGAIDKAKVRAYFDQYETEHPEWAQAVQHVKAFCLASELRAQGVFLNCPAYDIMQCVLASFIKHAS

PSVWSTATDCAYPKAYAADCPVCPSDCYSPQIPYGSCNACYTQPRTV

>SlittoOBP2

MVRKISGLLCCLCVFGISFSDSAISADSESRCRNPPTAPQKIERVITLCQDEIKLSILREALDVIKEEHTMPAQRRRD

KREVPFTHDEKRIAGCLLQCVYRKVKAVDGYGFPTLEGLVGLYSDGVNERGYFMAVLEASRECLMKNHDKFSRT

VPMDNGRNCDISFDIFECISDRIGEYCGTSGL

>SlittoOBP3

MKSFVVICIVFVVGVCATEKGNKIASECIKESGVKSDVLAEAKKGNLGDDPAFKEFTYCFFKKVGIVGEDGKLNRD

VAIAKLPSGVDKAEAEKLLDSCKSKTGKDAVETVYEIFKCYQHGTKSHIMFAS

>SlittoOBP4

MKTLLVFAACILVAQALTDEQKEKLKKHRTECLTETKVDEQLVNKLKGGDYKMDNEALKKYALCMMMKSELMT

KDGKFKKDVALAKVPNPADKPTVEKLIDACLANKGNTPHQTAWNYVKCYHEKDPKHAIFL

>SlittoOBP5

MTKVLFAIVLTMVTFAVVLSASTKEAMTTTMSDQVNSIDVDVLAVMDMCNDSYRIDPTYLQALNESGSFIDET

DKTPKCFIRCVFENVGIVSEDGKQFNPARAAVIFAGERNGKPMEDIADMTALCATDRQETCPCDRSYKFLRCLM

SMEIERYEKS

>SlittoOBP7

MFTEALPLFVILVAVTHGGKNKPVFSDEIKEIIQTVHDECVAKTGVAEEDITNCENGIFKEDAKLKCYMFCLLEEAS

LVDDDDTVDYDMLVSLIPDEYYERTTKMIFACKHLDTPDKDRCQRAFEVHKCSYEKDPDLYFLF

>SlittoOBP10

MKEGNRYSHERRITNDSGDQLMVINATDDDYSGYGSGNMGEKLLTSVPRPATPSNNINKNNINRTKRNEPLLN

RPDSDQCLSQCVFANLQVVDSKGIPREAELWNKVQSSVTSQQSRSALHDQIQACFQELQSEAEDNGCSYSNKL

ERCLMLRFSDRKVDGKGNAKKSSTEQTG

>SlittoOBP11

MSKFTCLVLCVVAVSLNGVHATAEEKAAFIEAVKPYVQECSKEHGVTPEDIKSAKAAGNADGINSCFLSCVYKKA

EVINEKGEYDVDKALEKLKKFVSNEDDYAKFANIGKKCASVNEKSVSDGEAGCERAALLTSCFLEHKSEISA

>SlittoOBP12

MSVVRCSSFLVALFCFVSVNAMSGDEEAGIKEALRPFVQECADEFGITEEQFEEAKKKASAADIDPCFMSCFLKK

AEFFDSQGKFDVDSTMAFAKEHLTSEPAMKFVEAVGDECVKINDEDVSDGDKGCDRAKLLFECIAETKKKME

>SlittoOBP13

MITSSSLLVLTAVVQVLFAQQPVFESGPPEPWGPPQRPAHRRQFLPRIPKRCWVPPQRINVYNCCPIPTLYPDED

MQSCGFEKTSGNTDQPQKPVFRPEGTCKEGYCVMGKFDLLFANNSVDFVKFREYLDNWAESYPEFANAIRIAK

QECAQDGGPEVPPICEPDKLFLCLTSTIFWNCKLRDGDGCAALQEHMNECKQYYTRQMEPTMKDIEVR

>SlittoOBP14

MDQKRICLFVIAMFLASGSDAMSRQQLKNSGKMLKKNCMNKIGVTDDQVGSIDKGKFIEDRKVMCYIACIYEL

TNVIKNNKLNYEASIKQIDLMYPPDVKESAKAAVEKCKDVQKKYKDICEASFYAAKCMYEYKPEDFIFA

>SlittoOBP15

MFNNCFVYSMTREQIKNSGKLIKKTCSAKNDLTEDEVKDVDKGKFIEKKDFMCYIACVYKMGQTVKGSTINHD

MMLRQVDMMFPNDMKAPVKAAIEHCRPVAKNYKDLCEASYWTAKCIYDFDPANFMFP

>SlittoOBP16

MLAEELKDCFDGSGPKDPMKCEIDLCIAKKKGFATDDGKLDIKKFEEVITKDVGSDKDLLDEIKTNCINGDLNNY

GPPEFCDFIKIKHCVTLHMMNHCSEWSDDGNCKVVKELVGKCAKVI

>SlittoOBP17

MRTFRLLCCILSIFFIFDQSYGMTRQQLKNSGKLMKKSCMPKNDVTEDEVGDIEKGKFIETRNVMCYIACVYTM

SQVVKNNKLSYEAVIKQVDVMFPAEMRDAVKAAATHCKETTKKYKDLCESSYWTAKCMYDYDAQNFVFP

>SlittoOBP18

MILXYTQKLTNMLLTKIVKFFILVATCEAMTMKQIKNTGKMMRKTCQPKNNVEDEKIDPLSDGVFIDEKEVKCY

MACIMKMANTIKNGKLNYDAAMKQADLLFPDDIKEPAKEAITACRKVADAHKDICDASFHVTKCIYNHNPSIFY

FP

>SlittoOBP20

MWVQALVLTLATLVTLVAAAVEMDEDMAELARMVRDNCAGETGVDVALVEQVNAGAELMPDDKLKCYIKCT

METAGMMADGEVDIEAVLALLPPSLAEHNAPALRACGTQRGADHCDTAFRTQQCWQNANKADYFLI

>SlittoOBP22

MNRLLLVYLVVLYAGCSYGMTRAQVKKTMGIIKNQCMPKNSVTEEQVGRIEQGVFIEDRNVMCYVACIYKSLQ

VVKNDKLDMALITKQIDILYPPELKEPVKKSVAACFHSQDNYSDFCEGVFYASKCLYEKDPASFIFP

>SlittoOBP26

MFNSSVFMYCLYFCALTPYLVSAMTAEQKALIHEHFETIGKSCNKDSTMITAEDIANLRAKKIPTGPNAPCFLAC

MMKQIGVMDDNGMVQKETALEMAKAVFDDPEEIKAIEDYLHSCSHINTESVSDGAAGCERAMLAYKCMTEN

ASKFGFDI

>SlittoOBP25

MSKLSCIVFCAVAMRLCVFVASEDANSIFHAPIKPIVIDCAKEYGLSEDDIKKNRGLDGLKNLPPCFIRCVLNKLDII

NDKGQYDADSGIATIKGLMSNNEYLEKISGVLKECESVNEKSVSDGDAGCERALLGAMCYLDHKTIVLA

>SlittoOBP24

MYKIISLVFCIAVCLNRVHGNAEDKIAIMTAVKPFVEECAKKHGVTFEALLTAKASGKIDGVEPCFYSCVYKKTEFL

NSKGEYDVDTALAKLKKYISNDDDYAKLSQVGKRCASVNSKPVGDGEAGCERGVLLTQCFLDHKGSVPM?

>SlittoOBP28

MIVRFLLCLYIVEFYGVHVQARTDQEIKAWFFREGMDCNIEHPISXKEMLELKENKIPDTNNAKCFVACVFKKTG

MLDSXGMFDAENSIAMTQKDFANDPNRLESSKKLLETCKKVNDETVSDGEKGCERSVLLHKCFVETAPQLGIKL

P

>SlittoOBP23

MAKLSCLVLCVVAASLGSIHVAKGESLRESLRPVIVACSKEHGVTDAEIQAAKEAGSPASIKPCFIACVFKKAGFIN

EQGQLDLETGLKNLRQFVKDDEQYKQLEGVANKCSPVKDKAVSDGAAGCDRGVHLAGCFLDHKTSIII

>SexiPBP1

MAGAKWQFVCVVFALYLTSAALGSQELMMKMTKGFTKVVDDCKAELNAGEHIMQDMYNYWREDYQLINR

DLGCMILCMAKKLDLMEDQKMHHGKTEEFAKSHGADDEVAKKLVSIIHECEQQHAGIADDCMRVLEISKCFRT

KIHELKWAPNMEVIMEEVMTAV

>SexiPBP2

MAFCRSATMSVRVALVVAASMLVVVQASQDVMKNLAINFAKPLDDCKKEMDLPDSVTTDFYNFWKEGYELT

NRQTGCAILCLSSKLEILDQELNLHHGRAQEFAMKHGADETMAKQIVDMIHTCAQSTPDVAADPCMKTLNVA

KCFKLKIHELNWAPSMELIVGEVLAEV

>SexiPBP3

MGSHNVFVALVLLAVGMRVAEPSKDAMKYITSGFVKVLEECKQELNMNDHIIADLFHFWKLEYALLSRDTGCVI

ICMSKKLDLLDANGRMHHGNAQEFAKRHGAGDDVASKIVQIIHDCEKKHERDDDECLRVLEVAKCFRTGIHDL

DWQPKVEVIVSEVLTEI

>SexiGOBP1

MLFLLRALPLLAAVLPLRADVNVMKDVTLGFGQALDKCRQESQLTEEKMEEFFHFWRDDFKFEHRELGCAIQC

MSRHYNQLTDSSRMHHDNTEQFIKSFPNGEVLARQMVELIHSCEKQYDHEDDHCWRILHVADCFKQGCVQR

GIAPSMEMMMTEFIMEAEAR

>SexiGOBP2

MTAEVMSHVTAHFGKALEECREESGLSAEVLEEFQHFWREDFEVVHRELGCAIICMSNKFSLLQDDTRMHHV

NMHDYVKGFPNGHVLSEKLVELIHNCEKRFDSMTDDCERVVKVAACFKVDAKAAGIAPEVAMIEAVMEKY

>SexiOBP1

MSKFTCLVLCVVAGCLSGVHATAEEKAALIEAVKPYIQECSKEHGVTPEDIKSAKEAGNADGINACFLRCVYNKA

GVINDKGEYDADKALEKLKKFVSNEDDYAKFAEIGKKCASVTETSVSDGEAGCERAALLTSCFLEHKSEVHA

>SexiOBP2

MKSFVVFCIVLVVGVCANEKGNKLDRPFASECIKETGVKNELLEEAKKGIISEDPAFKAFTYCFFKKIGIVGEDGLL

NRDVAIAKLPSGVDKSEAEKLLDSCKSKTGKDAVDTVFEIFKCYQQGTKSHIMFAS

>SexiOBP3

MVKLTCVVFCAVAMALSVFVAGEDANSVFQGAIKPLIAECAKEYKLSDEELLKNRGLAGLSNLPPCFIGCVLKKF

DIINDKGLYDAEAGIAKIEKLLPNNEFLDKISGVLKSCESANEKSVGDGDAGCERAVLVATCYLEHKTAVIA

>SexiOBP4

MWNFLVVFLAICSCVYGLTEEELKMEFTKLIMKCNKDGKVDMTELVQLQNYVVPTKQTTKCVLACAYKAAEVM

NAKGEYDIDHAYKVAEMMKNGDEKRLVNAKKMADLCVKVNEQSVSDGEKGCDRAAMIFKCTVENAPKFGFK

L

>SexiOBP5

MTMKQIRNTGKMMRKTCQPKNNVEDEKIDPIAEGVFIDEKEVKCYMACIMKMANTIKNGKLNYDAAIKQAD

LLLPDDIKEPAKEAITACKKVADAHKDICDASFHITKCIYNHNPGIFYSP

>SexiOBP6

MLGSLLFVFAFSVFSLGAEALLIDDLKQKYADSILQCSQQYPLDRADAELLQNKVMPDKESTKCLFACVYKVTGV

MSDQGELSVEGVNALSQKYLADDPEKLKKSEEFTEACRTVNDAPVSDGARGCDRAALIFKCTIEKSPDFSFV

>SexiABP1

MSVVRYSSFVVALFCLVSVNAMSGDEEAGVRDALRPYVQECADEYGITEEQFEEAKKKASADDIDPCFMSCFLK

KAEFFDAQGKFDVDSTMAFAKEHLSSEPAMKFVEAVGDECVKINDEDVSDGDKGCDRAKLLFDCIAETKKKM

D

>SexiOBP10

MDRKRICLFVIAMFLASGSDAMSRQQLKNSGKMLKKNCMNKIGVTEDQVGSIDKGKFIEDRKVMCYIACIYEL

TNVIKNNKLNYEASIKQIDLMYPPDIKESAKAAVEKCKDVQKKYKDICEVSFYAAKCMYEFKPEDFIFA

>SexiOBP8

MARRQQGAMFTETLPLFVILVAVTHGGKDKPVFSDEIKEIIQTVHDECVAKTGVAEEDITNCENGIFKEDAKLKC

YMFCLLEEASLVDDDDTVDYDMLVSLIPDEYYERTTKMIFACKHLDTPDKDRCQRAFEVHKCSYEKDPDLYFLF

>SexiOBP9

MKTLFVFAACILLAQALTDEQKEKLKKHRTECLSETKVDEQLVNKLKGGDYKTESEPLKKYALCMMMKSELMTK

EGKFKKDVALAKVPNPADKPTVEKLIDACLANKGNTPHQTAWNYVKCYHEKDPKHAIFL

>BmanPBP1

MSIKGQIALALMVYMAVGSVDASQEVMKNLSLNFGKALDECKKEMTLTDAINEDFYNFWKEGYEIKNRETGC

AIMCLSTKLNMLDPEGNLHHGNAMEFAKKHGADETMAQQLIDIVHGCEKSTPANDDKCIWTLGVATCFKAEI

HKLNWAPSMDVAVGEILAEV

>BmanPBP3

MARYNIVVAVLVLGVVGARGSSEAMRHIATGFIRVLDECKQELGLTDHILTDMYHFWKLDYSMMTRETGCAIIC

MSKKLDLIDGDGKLHHGNAQAYALKHGAATEVAAKLVEVIHGCEKLHESIDDQCSRVLEVAKCFRTGVHELHW

APKLDVIVGEVMTEI

>HassPBP1

MNFAKPLEDCKKEMDLPDSVTTDFYNFWKEGYEFTNRQTGCAILCLSSKLELLDQEMKLHHGKAQEFAKKHG

ADDAMAKQLVDLIHGCSRSTPDVTDDPCMKALNVAKCFKAKIHELNWAPSMDLVVGEVLAEV

>HassPBP2

MMGSAMSSKELLTKMSEGFTKVVDACKTQLNVGDHITQDMYNFWREEYQLVNRDLGCMIMCMVAKLDLIG

DDQKMHHGKAEEFAKSHGADDVLAKQLVSLIHSCETQHQAIEDHCSRVLEIAKCFRTKIHELKWAPSMEVVME

EIMTAA

>HassPBP3

MGSRHVFFAFAVLAVSVRKAEPSKDAMQYITSGFVKVLEECKHELNLNEQILADLFHFWKLEYSLLGRDTGCAII

CMSKKLDLLDANGRMHHGNAAEFAKKHGAGDEVASKIVTIIHECEKKHEQDGDECLRVLEVAKCFRTGIHELD

WQPKVEVIVSEVLTEI

>HassGOBP1

MDINVMKDVTLGFGQALDKCREESQLTEEKMEEFFHFWSDDFKFEYRELGCAIQCMSRHFNLLTDSSRMHHD

NTEKFIQSFPNGEVLARQMVELIHSCEKQFDHEDDHCWRILHVAECFKGSCVQRGIAPSMELMMAEFIMEAE

SL

>HassGOBP2

MTSKSCLLLVAMATLTASVMGTAEVMSHVTAHFGKALEECREESGLSAEVLEEFQHFWREDFEVVHRELGCAII

CMSNKFSLLQDDSRMHHVNMHDYIKSFPNGHVLSEKLVELIHNCEKKYDTMTDDCDRVVKVAACFKVDAKA

AGIAPEVAMIEAVMEKY

>HassOBP3a

MSKFTCFVLCVLAVSLAEVRSNALEKAAIRAALYPLIVDCAKEHSVTLEQLKAAKAAHSAQGINPCFQSCVYKKT

GIFNDNGEYDIANAKTKLQKFVTDEDEYARIAEVGKTCASVNDKSVSDGAAGCERAALLTACFLEHRAQIII

>HassOBP17

MKTFVILAACVMLVQASGLTDEQKEKLKKHRSECLTETKVDEQLVNKLKGGDYKTESEPLRKYALCMMMKSEL

MTKDGKFKKDVALAKVPNAADKPTVEKLIDACLANKGNTPHQTAWNYVKCYHEKDPKHAIFL

>HassOBP7

MSRFCVLSFVVLLFCMENIYALSSEEELSIKEALHPFVVECAEEYGMTEEMFEEAKKKGSAEDIDPCFMSCFLKKT

GFFDDAGKFDAEKSISFAKEHITSESAIKFLVAGAGECVQINDEDVSDGDKGCDRAKLLFDCLTDLKKKLSE

>HassOBP13

MFTGTLPLVVFLATFAYGGKEKPVFSDEIKEIIQTVHDECVAKTGVAEEDITNCENGIFKEDPKLKCYMFCLMEEA

SLVDDDDAVDYDMLVSLIPEEYVDRTTKMIFSCKHLDTPEKDRCQRAFEVHKCSYEKDPDLYFLF

>HassOBP1

MCKLTCLVLAAVAVVFSNVNADDEPRASFRQMLGPFVMECKKEFDITEDDLKKAQQEHSPDALKPCFIACVFK

KFGIITSAGKYDSDASISRIKDVVKNDDLFAKLKSVTEKCNSVNDASVSDDDAGCERAALLAKCFIENKSEISFN

>HassOBP2

MLNLFIVVLALCSSVAVYALTEEELKLKFTKLIMKCNKDSEVDMQELVQLQSYVVPTKTATKCVLACAYKAASVM

NAQGLYDIDHAYKVAEMMKNGDEKRLTNAKKMTDICVKVNDIKVSDGEKGCDRAALIFKCTVDNAPKFGFKL

>HassOBP3

MSRQQLKNSGKMLKKNCMSKNQVTEDQIGSIEKGKFVEDKKVMCYIACIFEMTNVIKNNKLNYDASIKQIDL

MYPPDLKESAKAAAEKCKDVQKKYKDICEASYWTAKCLYDFKPEDFIFA

>HassOBP4

MSKFTCLLLCVVAVSLSKVHATEEEKEAIRAAVRPIMEACGKEHGVTLDDLKAAKAAHSADGIKPCFQSCVYKKA

GIFNDNGEYDIANAKTKLQKFVTNDEEYARIAAVGKTCASVNDKPVTDGAAGCDRAALLTACFLEHRAEIII

>HassOBP5

MSKFSCLVLCVVAASLSQAYASEEEKAAFREAIKPIVEECSKEHGVSHDELKSAKDNQNADNIKPCFLGCVYKKAE

VFNSKGEYDVDKALEKLKKFVSNDEAYAKFAEVGKKCASVNDKAVSDGDAGCERGALLTACFLEHKAEVPL

>HassOBP9

MCKFSVLFLYSAVMAVNIWSASCISEEDKAAIITAIAPLAQNCGSECGLDNDDFEKYKEDGSDMDPCFKACLMT

QMGVLDKEGKYDGKGLHKAMEEADYPGDKDDAQKFLDELDRCFDAKGDNSGSDEEAKMKRADVLFQCMQ

DMKEK

>HassOBP16

MFKSIVFCALVIVASHADVLKKRDSKGASLKPLSVCCDIPELGDPKNLEKCSNPKMPGPCDDIQCISEASGFLIDR

NTLNVDAYKAHLTKWEEEHKSWKVAVDRAIEECANNQTRQYLDFPCKAYDVFTCTGIAMLKKCPEAAWKC

>MsexPBP1

MKVAVVAIVVYLAVGNVDSSPEVMKNLCLNFGKALDECKAEMNLSDSIKDDFANFWVEGYEVSNRDTGCAIL

CLSKKLDMIDPDGKLHHGNAMEFAKKHGADEAMAKQLLDIIHNCENSTPPNDDACLKTLDIAKCFKKEIHKLN

WAPNMDLVVGEVLAEV

>MsexPBP2

MVSTKWRLLVVTIAVLTMEMVSASQEVLKQMSVGFSKVLQTCKTELSVGDHIIQDFYNYWREDYDLLNRDFGC

MVICMAVKHDLINDQLTMHHGNAHAFAKTHGADDDTAQQLVTILRECEAKHQSVEDVCNRALEMAKCFRTK

IHELKWAPAMEVVLEEIMTSV

>MsexPBP3

MAIIPIFTVLLMMTAVKEIAPSSDAMRHIANGFLKVLDQCKHELGLTDQIVVDLYQFWKLQYALLNRDTGCAIIC

MSKKLDLLDSTGRMHHGNTQEFAVSHGATDEVASKVVVIIRDCEKQQEGEEDDCVRVLEVAKCFRTAIHELNWAPNMEVVVDELLTER

>MsexGOBP1

MGQDTRSLVLVVLVGLVGAVSADVQVMKDVTLGFGQALEQCREESQLTEEKMEEFFHFWREDFKFEHRELGC

ALQCMSRHFNLLTDSSRMHHENTDKFIKSFPNGAVLSKTMVELIHNCELQHDAEEDHCWRILRVAECFKISCTK

AGIAPSMEVMMAEFIMELKQ

>MsexGOBP2

MVNRLILMVVVVFITDSVMGTAEVMSHVTAHFGKALEECREESGLPVEVMDEFKHFWREDFEVVHRELGCAII

CMSNKFELLQDDTRIHHVKMHDYIKSFPNGQVLSEKMVQLIHNCEKQYDDIADDCDRVVKVAACFKKDAKKE

GIAPEVAMIEAVIEKY

>MsexABP1

MSVISFFVLCFGVLAVSVGAVSENERNQISQSILPHIVKCSQEYGVSEGQIKDAKESVNPLGLNPCFLGCVLKSAGI

IDKNGLFDVEATKEKSKKYISSEKDVTNFDKIIKDCTEVNQKNVSDGNKGCDRAKELVTCFLAKRGDFSVFTF

>MsexABP2

MDRKDLCLLIIAFILADGVDSMSKQQLKNSGKMFKKQCMGKNKVTEDEIGEIDKGRFVEQQNVMCYIACIYQ

MSQVVKNNKLNYEASLKQIDIMYPPELKDTAKGALEACKDIAKKNKDLCEASFKTAKCMYEYSPKDFLFP

>MsexABP3

MITATLHVVFALLGFVYGAKNKPVFSEEIKEIIQTVHDECVGKTGVSEEDIANCENGIFKEDVKLKCYMFCLLEVA

GLADEDGTVDYDMLVSLIPEEYSERASKMIFACNHLDTPEKDKCQRSFDVHKCTYEKDPEFYFLF

>MsexABP4

MFRYIFCAICLILILFDASYAMSRQQLKNSGKMMKKSCIPKNDVTEDEVGQIEQGKFIEDRRVMCYIACIYTMTQ

VVKNNKLSYDAIVKQVDMMFPPEMRTAVKTAAENCKDIAKKYKDICEASYWTAKCMYDFDSKNFVFP

>MsexABP6

MKVTNGLFLVFLVSVLACGIAASPKKIYRIPPQASEKIVEEVLKCVQKMGLDSTVVNLLKEGKYTEDDRVIETLMC

SNQNVGNVNGDGKVNIDKVMNDIFSNKPEIRSALVACEKDGGKSPLETFKNFILCFKEKVPVKVML

>MsexABP7

MQACLFLTLVLAVVGLNAHNVHLTDGQKEKANEPIAACIKETGIKPEVIAEAKKGHYSEDEAMKKFILCFFHKAGI

VNADGKLNLDVAIAKLPPGVDKTEATKTLEGCKDNGGKDAADPAFAIFKCYKDATKTHVLF

>MsexABP8

MKALLVLAACLVLAQALTDEQKEKLKKHKSECLSETKVEEQLVNKLKAGDYKAENDNLKKYALCMMMKSELMT

KEGKFKKDVALSKVPNPADKPMVEKLIDTCLANKGNTPHQTAWNYVKCYHEKDPKHAIFL

>MsexABPX

MISSLVHVLTLLAAGALALDEEQAELARMVRENCVDEIGVDEGLLAKVDDGADLMPDPKLKCYLKCTMEMAG

MISDGVVDVEAVLGLLPDDVKLRTTDIVRACDTQKGADDCDTAFLTQTCWQQANRADYIFI

>MsexOBP10

MIRAVVFCCCMVALMPFSANAMTDEQKEKIHEHFEKLGLGCLKEYTITEDDIKDLRAKKVPSGENAGCFLACM

MKEIGVLNDEGMLEKGRAMELAKEVFDDAEELKKIEESMHSCSSVNSESVGDGEKGCERAMLAYKCMVENAS

KFGFDI

>HvirPBP1

MMSVRLMLVVAVWLCLRVDASQDVMKNLSMNFAKPLEDCKKEMDLPDSVTTDFYNFWKEGYEFTNRHTGC

AILCLSSKLELLDQEMKLHHGKAQEFAKKHGADDAMAKQLVDMIHGCSQSTPDATDDPCMKALNVAKCFKAK

IHELNWAPSMELVVGEVLAEV

>HvirPBP2

MMGSAMSSKELLTKMTGGFTKVVDHCKTELNVGDHIMQDMYNFWREEYQLVNRDLGCMIMCMTAKLDLV

GDDQKMHHGKAEEFAKSHGADDALAKQLVGLIHGCETQHQAIEDHCSRTLEVAKCFRTKIHELKWAPSMEVIMEEIMTAA

>HvirGOBP1

MPGVLRALLLLAAAAPLLADVNVMKDVTLGFGQALDKCREESQLTEEKMEEFFHFWRDDFKFEHRELGCAIQC

MSRHFNLLTDSSRMHHDNTEKFIQSFPNGEVLARQMVELIHSCEKQFDHEEDHCWRISHLADCFKSSCVQRGI

APSMELMMTEFIMEAEAR

>HvirGOBP2

MTSKSCLLLVAMVTLTTSVMGTAEVMSHVTAHFGKALEECREESGLSAEVLEEFQHFWREDFEVVHRELGCAII

CMSNKFSLLQDDSRMHHVNMHDYVKSFPNGHVLSEKLVELIHNCEKKYDTMTDDCDRVVKVAACFKVDAKA

AGIAPEVTMIEAVMEKY

>HvirABP1

MSKFSCLVLCVVAASFSQAFASEEEKTAFREAIRPIVEECSKEHGVSHDELKSAQENQNADNIKPCFLGCVYKKSE

VFNSKGEYDVDKALEKLKGFVSNEAAYAKFAEVGKKCVTVNDKPVSDGAAGCERGAMLTACFLEHKAEVPL

>HvirABP2

MSRFCLLSFVVMIIYLGSIHALSSDEESSIKEALHPFVVECAEEYGITEEMFEEAKKKGSAEDIDPCFMSCFLKKAEF

FDGAGKFDVEKTMSFAKSHITSEPAIKFLEAAGGACVKINDEDVSDGDQGCDRAKLLFDCLMELKKKISE

>HvirABPX

MCARSLTLVTLLAALGAARAVAMDEDMAELARMVRENCAAETGADVALVERVNAGADLMPDDKLKCYIKCT

METAGMMADGEVDIEAVLALLPPELAEHNAPSLRACGTVRGADHCDTAFRTQQCWQNANKADYFLI

>HvirOBP2

MMGSAMSSKELLTKMTGGFTKVVDHCKTELNVGDHIMQDMYNFWREEYQLVNRDLGCMIMCMTAKLDLV

GDDQKMHHGKAEEFAKSHGADDALAKQLVGLIHGCETQHQAIEDHCSRTLEVAKCFRTKIHELKWAPSMEVI

MEEIMTAA

>HvirOBP21

MDRKKMCLLIIAMFLAIGCDAMSRQQLKNSGKMLKKNCMNKNQVTEDQIGTIDKGKFVEDKKVMCYIACIYE

MTNVIKNNKLNYDASMKQIDLMYPPDVKDSAKAAVEKCKDVQKKYKDICEASFWTAKCMYDFKPEDFIFA

>MbraPBP1

MTYSKWRLSCLVCVIFVASSVMASKELITKMSSGFTKVVDQCKNELNVGEHIMQDMYNFWREEYELLNRDLG

CMVMCMANKLDLIGEDQKMHHGKAADFAKSHGADDDQAKQLVGIVHDCENTHQGVEDACSRALEVAKCF

RSKMHELKWAPSMEVIMEEIMTAV

>MbraPBP2

MALHRSTTMSVRLALVAIASLFITVECSQEIMKNLSMNFAKPLEDCKKEMDLPDSVTTDFYNFWKEGYEFTNR

QTGCAILCLSSKLELLDQELKLHHGKAQEFAQKHGADEAMAKQLVDLIHGCTQSTPDVAADPCMKALNVAMC

SKTKVHELNWAPSVELIVGEVLAEV

>MbraGOBP2

MTSKSSLFLVVLATVTSSVMGTAEVMSHVTAHFGKALEECREESGLSAEVLEEFQHFWREDFEVVHRELGCAIIC

MSNKFSLLQDDSRMHHVNMHDYVKSFPNGEVLSGTLVELIHNCEKKYDGMTDDCDRVVKVAACFKVDAKAA

GIAPEVAMIEAVMEKY

>AperPBP1

MLGKISLLLLPVFVAINLVHSSPEIIKNLSQNFCKAMDQCKQELNIPDSVIADLYNFWKDDYVMTDRLAGCAINC

MATKLDVVDPDGNLHHGNAKEFAMKHGADASMAQQLVDIIHGCEKSAPPNDDKCMKTIDVAMCFKKEIHKL

NWVPDMDVVLGEVLAEV

>AperPBP2

MIRKVLLSVLLAVLMTINLGQASPEVMKNLCMNYGKAMDQCKQELNLPDSVIADLYNFWKDDYVMTDRLAG

CAINCLSTKLDIVDPDGNLHHGNAKEFAMKHGADDGMAHELVDIIHGCEKSSPPNDDKCIKTMDIAMCFKKEIHKLNWVPNMDLVVGEVLAEV

>AperPBP3

MIAKTFNLLVIVYLSTNTAVDSSQDVMKSMTLTFTKGLDACKKEMDLPDTIDVDFNNFWKEDYVVTNRNAGCA

IMCLASKVDLVDSMGILIHGSSHEFAKQHGADDNMAKQLSDTLHSCEKTIGTLNDECLRALNVANCFKVEIHKL

DWAPSMDLIIGEILAEI

>AperGOBP1

MAHTLQTVVLLLGTSILHPILADVNVMKDVTLGFGQALEKCREESQLTEEKMEEFFHFWSEDFKFEHRELGCAIL

CMSRHFNLLTDSSRMHHENTDKFIKSFPNNEVLSKHMVNLIHSCEQQHDADLDHCWRILRVAECFKRSCQEA

GVAPSMELLMAEFIMESEIN

>AperGOBP2

MGYKLLLMYIAIVIDSVIGTAEVMSHVTAHFGKALEECRDESGLSPEILNEFKHFWSEDFDVVHRELGCAIICMS

NKFSLLKDDTRIHHVNMHDYVKSFPNGEVLSAKMVNLIHNCEKQYDDITDECDRVVKVAACFKVDAKKEGIAP

EVAMIEAVIEKY

>ApolPBP1

MLRKISLLLLPVFVAINLVHSSPEIMKNLSNNFGKAMDQCKDELSLPDSVVADLYNFWKDDYVMTDRLAGCAI

NCLATKLDVVDPDGNLHHGNAKDFAMKHGADETMAQQLVDIIHGCEKSAPPNDDKCMKTIDVAMCFKKEIH

KLNWVPNMDLVIGEVLAEV

>ApolPBP2

MSLSLFLAMLVTINLVQASPEIMKNLCMNYGKTMDQCKQELGLPDSVINDLYNFWKDDYVMTDRLAGCAINC

LSTKLDIVDPDGNLHHGNAKEFAMKHGADDGMAQQLVDIIHRCEKSTPPNDDKCTKTMDIAMCFKKEIHKLN

WVPNMDLVVCEVLAVV

>ApolPBP3

MIAKTFNLLVIVYLSTNTGVDASQEIMKTMTLTFTKGLDACKKEMDLPDTVDVDFNNFWKEDYVVTNRDAGC

AIVCLASKINLVDSMGILIHGSAHEFAKQHGADDNMAKQLSDTLHTCETIIGTGNDECTRALHVANCFKVEMHK

LDWAPSMDLIIGELLAEI

>AsegGOBP1

MTHPGQALVLVLLAAAALADVSVMKDVTLGFGQALDKCRQESDLTEEKMEEFFHFWRDDFKFEHRELGCAIQ

CMSRHFNLLTDSSRMHHDNTEQFIQSFPNGEVLARQMVSLIHGCEKQFDHEEDHCWRILHVAECFKHACVAT

GVAPSMEMMMTEFIMEAEAR

>AsegGOBP2

MTLKCCLLLVVVAAVTRSAVGTAEVMSHVTAHFGKALEECRDESGLSAEVLEEFQHFWREDFEVVHRELGCAII

CMSNKFSLLQDDSRMHHVNMHDYVKGFPNGEVLSGKLVELIHNCEKQYDTLTDDCDRVVKVAACFKVDAKA

AGIAPEVAMIEAVMEKY

>AsegPBP1

MSVRLALFMIAGVFITVECSQEIMKNLSLQFAKPLEDCKKEMELSETVITDFYNFWKEGYEFTNRQFGCAILCLS

SKLELLDQDLKLHHGKAQEFAKKHGADEAMAKQLVDMIHGCSQSTPDVADDPCMKTLNVAKCFVAKIHDLK

WAPSMDLIMGEVLAEV

>AsegPBP2

SQKVVASFSKGFKDVVDHCKAELNMGEHIMQDVYNFWREEYQLVNRDFGCMVLCTANKLGLLKEDQKMHH

DKAEEYAKKHGADDATAKQVVAIIFECENNNSGMDDECNRALEIAKCFRTKMHELKWAPSVEDAIEEIMTAVM

>AsegPBP3

MGTYNVFFAFVLMAAGVREIEPSKDAMKYITSGFVKVLEECKQELNMNDRIIADLFHYWKLDYTLLNRDTGCAI

ICMSKKLDLLDDTGRMHHGNAQEFALKHGAGEEVASKIVTIIHDCEKKFERDDDECLRVLEVAKCFRTGIHDLD

WQPRVEVIVSEVLTDM

>AsegOBP2

MSKFTCLVLCLVAASISRVYADEDANKAAFREAIKPIIDECSKEHGVSNDDIDAAKKAGSADAIKPCFFGCIYKKAE

VFNAKGEYDVDNALSKLKKFVPDEAKFAKYAEIGKKCASVNEKPVTDGEAGCERGAMLTACFLENRAEILI

>AsegOBP3

MIRSCRCLVFAAVFQVVLGQGLTGTDSGPPGFQRPQSYVPKHCFAPPPGVDLHSCCPIPQLFPDEDMESCGIQK

LTKEQYDNPSPARVPCKESFCLLRNANLLKANNNSIDYAKMEDFVDSWAKMDPDFTMPITNAKKVCLIEGGPP

SPPVCEPDRVFTCLTSYVLWNCKLKLEKGEGCKILKEHMDGCRPFLAGP

>AsegOBP4

MCSCYSPCFMFGFQVCGILFFHHSPPSIFSLRSCIQPHINFALSHPLSPSLTSSSFTLAHSEPTASMNAIARSLVKW

ASTNSLVLSTSNLPALSKNPDSFIKQLMKQGSIPSALCFSFASSNCSSVIPYSSAHFTTNG

>AsegOBP5

MKFLVICVALVAGIHANVTLPPEQSEKALKTASECIKETEVSSEVLAEAKKGNIPEDDGLKKFALCFFKKAGIVDND

GKLILETAVAKLPPGVDKVEAKKVLEGCQSKSGKTPQETAFEIYKCYHAGAKTHIALAGI

>AsegOBP6

MFKYSVFVLFVVASTQADVLSQRENKGASLKPLSVCCDIPELGDPKHLAKCSNPKLPGPCNDVQCVFEESGFLTD

KNTLNKEAYRHHLKQWEENNKGWTIAVDKAIKECVDSDPRQHLDFPCKAYDVFTCTGIAMLKKCPDSAWKC

>AsegOBP8

MYLRSASKGVHSFPYGESTYTTKIVEFCSKETGITREVPPEEKGVKFSERKGLREFNDCYLEKTGFVTCDGTLNIDE

SLAKLPPGFAKPFVEHCQANIILKYIQDNVNDFSTCFHQEVQNHFLSFYGFTNYWVMLVLGTSFEKTRFTTLFYD

KNFDFWLAERAGFVEL

>AsegOBP9

MFHLYFYVFICGVLSFNVKAASLDELKMKYVEMIIECSDTYPITAADTLQLKTKTMPDNESIRCLFACVYKKAGM

MNEQGELSVEGVNEMTRRYLSDDPDKIKKSEQFTEACKSVNDVPVSDGTRGCDRAALIFKCTVEKSPDFDLL

>AsegOBP13

MVLIDIVKFLILVAMCEAMTMKQIRNTGKMMRKSCQPKNNVEDEKIDPIAEGIFIDETEVKCYMACIMKMANT

LKNGKLNFDAALKQADLLLPDEVKEPAKEAIIACKKAAEGHKDICDVSFHVTKCIYNQNPGIFYFP

>HzeaPBP

MMSVKLALVVAAWLFIRVDASQDVIKNLSMNFAKPLEDCKKEMDLPDSVTTDFYNFWKEGYEFTNRQTGCAI

LCLSSKLELLDQELKLHHGKAQEFAKKHGADDAMAKQLVDLIHGCAQSTPEVVDDPCMKTLNVAKCFKAKIHEL

NWAPSMDLVVGEVLAEV

>HzeaGOBP2

MTSKSCLLLVAMATLTASVMGTAEVMSHVTAHFGKALEECREESGLSAEVLEEFQHFWREDFEVVHRELGCAII

CMSNKFSLLQDDSRMHHVNMHDYVKSFPNGHVLSEKLVELIHNCEKKYDTMTDDCDRVVKVAACFKVDAKA

AGIAPEVAMIEAVMEKY

>OnubPBP1

MGLSLRLLVVVAAAIFGAESSQDVMKQMTINFGKALDTCRKELDLPDSINADFYNFWKEGYELSNRQTGCAIM

CLSSKLDLVDPEGKLHHGNTHEFAKKHGADDSMAKQLVELIHKCEGSVADDPDACMKVLDIAKCFKAEIHKLN

WAPSMDLIVAEVLAEV

>OnubPBP2

MWLSKTPVVIAVMCSMSVVVHSSQAVMKDMTKNFIKAYEVCAKEYNLPEAAGAELMNFWKEGYVVTSREA

GCAILCLSSKLNLLDPEGTLHRGNTVEFAKQHGSDDAMAHQLVDIVHACEKSVPPNEDNCLMALGISMCFKTEI

HKLNWAPNHELMLEEMMAEMKQ

>OnubPBP3

MWLPKTLVVMAVMSSMSVVVHSSQTVMREMTRNFIKAYEVCAKEYNLPEATGSELINFWKEGHELTTREAGCAILCMSTKLNLLDVQGSVHRGNTVEFAKHHGSDDAMAHQVVDILHACEKATPNEDKCMLALSIAMCFKAEIHKLDWAPNHELMFEELVSDMWNS

>OnubPBP4

MADATKWRVAAILVICFAVKLNTVMSSEEVMTKMGVTFFNVLEECKKELKVTTNINEGLVRFWSQGAAPEREL

GCVFLCMAHKKDLLEDQKRLHHENAHQFARGHGADDDKATEIVSLLRECEQQFITITDDCSRALEVARCFQAH

MQRLQWAPSMEVMVEEILAGMA

>OnubPBP5

MKGFAGIPVTLMLVLIGVSEIEMVPEAMKQLTGGFLKVLDQCKKELNLSDGVISDLYHLWKEEYDQISRDAGCVI

HCMSQKLELVGGDGKMHHVNIKDFALKHGAGDEIATQLVTLAHECEKQKASIEDDCERTLEMSKCFRSDVKQV

DWTPKMEVIITEVIEV

>OfurPBP1

MGLSLRLLVVVAAAIFGAESSQDVMKQMTINFGKALDTCRKELDLPDSINADFYNFWKEGYELSNRHTGCAIM

CLSSKLDLVDPEGKLHHGNTHEFAKKHGADDSMAKQLVELIHKCEGSVADDPDACMKVLNIAKCFKAEIHKLN

WAPSMDLIVAEVLAEV

>OfurPBP2

MWLSKTLVVIAVMCSMSVVVHSSQAVMKDMTKNFIKAYEVCAKEYNLPEAAGAEVMNFWKEGYVLTSREAG

CAILCLSSKLNLLDPEGTLHRGNTVEFAKQHGSDDAMAHQLVDIVHACEKSVPPNEDNCLMALGISMCFKTEIH

KLNWAPDHELLLEEMMAEMKQ

>OfurPBP3

MWLPKTLVVMSVMSSMSVVVHSSQTVMGEMTKNFIKAYEVCAKELNLSEATGLQLINFWKEGHELTTRETGC

AILCMSTELNLLDVQGSVHRGNTVEFAKHHGSDDAMAHQVVDILHACEKATPNEDKCMLALSIAMCFKAEIH

KLDWAPNNELMFEELVLDMWNS

>OfurPBP4

MADATKWRVAAILVICFTVNLNTVMSSEELMTKMGVTFFNVLEECKKELKVTTNINEGLVRFWSQGAAPEREL

GCVFLCMAHKKDLLEDQKRIHHENAHQFARGHGAEDDKATEIVSLLRECEQQFITITDDCLRALEVARCFQAH

MQRLQWAPSMEVMVEEILAGMA

>OfurPBP5

MKGFAGVPVTLMLVLIGVSEIEMVPEAMKQLTGGFLKVLDQCKKELNLSDGVISDLYHLWKEEYDQISRDAGCV

IHCMSQKLELLGGDGRMHHVNIKDFALKHGAGDEIATQLVTLAHECEKQKAAIEDDCERTLEMSKCFRSDVKQ

VDWTPKMEVIITEVIEV

>OfurGOBP2

MVCSGFYLGLVVMAAVTSVKGTAEVMSHVTAHFGKALEECRTESGLSPEILEEFQHFWSEDFEVVHRELGCALI

CMSNKLSLLQDDTRIHHVNMHDYVKGFPNGEVLSEKMVNLLHNCEKQFDDITDDCQRTVKVAACFKVDAKKE

GIAPEVAMIEAVMERY

>PxylABP

MKFLVVFAICLVAAQALTDEQKEKLKKHKTECLAETKPEVEHVDKLKNGDYTTENEALKKYAHCMMIKSELMTK

DGKFRKDVALAKVPNPADKPMVEKLIDTCLANKGDTPQQTAWNYVKCYHEKDPKHAIFL

>PxylPBP1

MWFPVRLSAVLLITLAVMDTQGSKETMKDITSGFFKVLNECKHELNLPDHLVGDFYHYWRQEYALLDRDLGCA

ILCMSRKLELIDASGKLHHGNTQEFAEKHGADNSMASKLVEVLHSCEKQHEAVSDECQRALEVAKCFRSSVHEL

GWAPTIDVIIEEVLTDM

>PxylGOBP1

MERRWCLLVLAAAAAGLPGVVRGTVEVMKDVTLGFGEALEQCREQSQLTEEMMEEFYHFWREDFKFEARAV

GCAIHCMSRYFNLLGEQQRMHHDNTHKFIQSFPNGEVLSHQMVGIIHTCEQQHDAETDHCWRILRVAECFKRESQAQGLAPSMEMLMAEFIMEADV

>PxylGOBP2

MASVWSLVVCGLMMAALPAARGTAEVMSHVTAHFGKTLEECREESGLSGEIMEEFHHFWREDFEVVHRELG

CAIICMSNKFQLMQDDARMHHENMHDYIKSFPKGDLLSETMVRLIHNCEKKYDDIDDECSRVVKTAACFKKD

AQAEGIAPELTMIEAVLEKY

>DindPBP1

MWVKMILITVAVVMMSVKVDLSQTLLKDMTKNFLKAYEQCQQELSLPESTAKELMFFWKDGYEVSSREAGCT

ILCLSKKMDLIDPEGKLHKGKSADFVKQHGTDDETANKVVDILHTCESNAAPNDDHCMVALAVALCFKKEIHNL

NWAPDPEVILEELMAEMS

>MsepPBP

MVLHRSATMSAHLALVVIASFLIAVECSQEIMKNLSINFAKPLEDCRKEMDLPDSVLTDFNNFWKEGYEFTNRQ

TGCAILCLSSKLELLDPEMKLHHGRAQEFAQKHGADEAMAKQLVDMLHSCMQTTPDDANDPCLKTLKVVTCF

KTKIHELKWAPSMDLIVGEVLAEV

>EposPBP1

MMNHKELVLFAVVCLSLYQAVEPSQDVVKDMSLNFRKGLDACKKELNLPDTINADFNRFWNDDHVVTNRDT

GCAIMCLSSKLELVTDTGLHHGNTLEYAKQHGADETVAQQIVDLLHTCAQAVPDLQDTCMKVLEWAKCFKAEI

HKLNWAPSAEVMAAEMLAEV

>EposPBP2

MATVSKWRMLVLTLCLTGVWQVESSADVMKKLTTGFATALEKCRDELNLPDAVMQDFFNFWREDYELVNRD

MGCAIMCMATKFDLVTEEQKLHHGNAHEFAKSHGADDSMAKQLVTMLHECETQTASISDDCGRTLEIAKCFR

TKIHGLKWAPSMETILEEVMTEV

>EposGOBP1

MATVSKWRMLVLTLCLTGVWQVESSADVMKKLTTGFATALEKCRDELNLPDAVMQDFFNFWREDYELVNRD

MGCAIMCMATKFDLVTEEQKLHHGNAHEFAKSHGADDSMAKQLVTMLHECETQTASISDDCGRTLEIAKCFR

TKIHGLKWAPSMETILEEVMTEV

>EposGOBP2

MASYWAVCVVLVAGSHLVAGTAEVMSHVTAHFGKALEQCREESGLSTAVLEEFQHFWRDDFEVVHRELGCAIL

CMSNKFSLMQDDARMHHENMHDYVKSFPQGEVLSAKMVELIHNCEKPYDDIKDDCERVVKVAACFKVDAK

KAGIAPEVAMIEAVMEKY

>CfumPBP

MLKQKELLLFAXVCLSLTQMVEPSQDVIKGMCLNFGKGLEECKKEMNLPDTVDADFYNFWKDXYVLTNRDTG

CAIMCLSNKLELVSDGKLHHGNTLDFAKQHGADETVAQQLVDLIHTCEKALPDLEDPCLKVLEWAKCFKIEIHKL

NWAPTMDVLAGEMLAEI

>LdisPBP1

MSAKLLLVLAVCLVMRVDCSKEVMKQMTINFAKPMEACKQELNVPDAVMQDFFNFWKEGYQITNREAGCVI

LCLAKKLELLDQDMNLHHGKAMEFAMKHGADEAMAKQLLDIKHSCEKVITIVADDPCQTMLNLAMCFKAEIH

KLDWAPTLDVAVGELLADT

>LdisPBP2

MMWLRLILAVAVCLVIPVEPSKDVMHQMALKFGKPIKLCQQELGADDSVVKEFLDFWKDGYVMKDRQTGC

MLICMAMKLELLDSAMEIHHGSTFAFAKAHGADEAMAQQIIDIVHGCTTTYPAAETNDPCQRAVNVAMCFK

AHVHKLNWAPDVELLVADFLAESQ

>CsupGOBP1

MEAAKVIMAGLLVVGVVPSMRADMVVMKDITLGFGAALEHCREESGLTQENMEEFFDFWREDFKFEHRELG

CALRCMSRYFNLITDTNRMHHENTENFIKSFPNGEKLSKVLVQVIHECEKKFDHEEDHCWRILHIGECFRDMCRSQNIAPAMEMLLAEFIMQAESDTNPVAL

>CsupGOBP2

MVCSGFYLGLVVMAAVTSVKGTAEVMSHVTAHFGKALDECREESGLSTEVLEEFKHFWSEDFEVVHRELGCALI

CMSNKLSLLHDDTRVHQVNMHDYVKSFPNGEALSEMMVKLIKNCERQYDNIKDDCDRTVKVAACFKADAKK

EGVAPEITMIEAVMEKY

>CsupPBP

MSLYMRIVVLALVYLFNGVESSQEIMKQLSLNFGKAYDSCKKELELPNEVDTDFFNFWKDDYAVTNRLTGCAIM

CMSNKLDLLDPDGKMHHGNAREFAKKHGADDSMAQQLIDILHNCEKGASPGPDGDACVQVLEISKCFKVEIH

KLNWAPSMDLIMAEVLADV

>CsupPBP3

MKCCLFGILVCFNVMVSDVESSQELIKKMSISFLKVLQECKLELSVPEEVLQSLMTFWNQDTDLSHRELGCVILC

VVSKLDLIELETYKLHPDNANEYVKKHGADDETASQIMNILRGCEIKNEAITDHCDRVREIAKCFHGHMHELKW

APNMEVIINELVATKAI

>CsupPBP1

MVRDTMMLKLVVVMCLTMTVVVDSSQTVMKSMTKNFLKAYEVCAKEYSLKEGTAGILIGFWKDDFSTTSRDV

GCAILCLSTKLDLIDPEGKLHHGKATEFAMQHGSGEEMAKKLVEILHNCEQTVTPNEDKCMRALDIAMCFKKEL

HTLGWAPDPELLFEELIAEMR

>CsupOBP

MRCCAVLFVLAFIGCIYAEQEIVHLPPEKVAQILPVAMQCVGESSVPPEVIFQYASGKSLGNDKKYQKFIHCVFTK

TGYADETGHINIDKAMEVFPKGTDKEAVKKIMEECSKERGEDPPETSFKFAKCFRKKAPVRIAL

>CsupOBP1

MASFHFKVNCFLYFVLLSSYFVYSMTRQQLKNSGKLLKKACIPKTNVSEEQIRDIDKGKFIGEKKIMCYIACIYTMS

QAIKNNKIQHDVMIKQVETMFPNDIKESAKFAIQQCRGIAKQHKDICEAAFWTTKCLYDADPATFIFP

>CsupOBP2

MGTVSTEHYPAHHVINMKAFIVLAVCIVAAQALTDEQKEKLKKHKSECLAETKVDEQLVNKLKAGDYKSDNEAL

KKYALCMLIKSELMTKEGKFKKDVALAKVANPADKPQVEKLIDTCSANKGNTPHQTAWNYVKCYHEKDPKHAIF

L

>CsupOBP3a

MFLKLLMLTVLFCAIHAMTRQQLKNSGKMLKKNCMGKNQVTEDQIGSIEKGKFIEDKNVMCYIACIYQMTQVI

KNNKLNYEASLKQVDIMYPAELKESAKKSIENCKHISSKYKDICEASYWTAKCMYEDNPKDFVFA

>CsupOBP11

MGFSRAVLLAAFVAGAWAMDEEMAELAKMLHDNCGEETGADLSLVDKVNAGADLMPDPKLKCYLKCIMET

AGMMTEGVVDVEAVLALLPDDMRAKNEQNLRGCGTQKGADHCDTAFLTQLCWQKANKADYFLI

>CsupOBP5a

MLLIIIAKFLVLVAICEAMTMKQIKNTGKMLRKTCQPKNNAADEKIDPLNEGVFIDEKEVKCYIACIMKMANTM

KNGRPNIEVAMKQADLLLPEELKEPAKEALTACRKVPDAHKDVCDAAFHLTQCVYNQNPDIFYFP

>CsupOBP6

MAKFVVLCLGLLAAALSVKALTKEELDHIKEATLMHFNECNKDFNVSEDDIKAAETQKNMDKIDACLIGCMMK

RSHLLDGEGKFDTEKAIELSKSFMKSEDDQKKFAEVVAECAKVNDEPVSDGANGCERSKMVLVCLAKHKAEFV

PARR

>CsupOBP5

MKLTLFPAFLLFTSILGLKDEHEENLKRWHMECFQETKVNPDLVLKLKMGNWQIKNKLLKEWILCVFNKYDLM

SKEGVFKLDTAMSLVPSADRDMIEDYIDACLPKHIAEPLDIAWKYAKCYHVGAKDPINKNKRLHYMTLFY

>CsupOBP7

MTSGSEQDIIKTPEETDTNPDLMAIMGECNETFRIETSYLESLNESGSFPDESDKTPKCYIRCVLMKTGVTTEDG

KFIPDVTSQVFASQNIKEQMDGIQNMATACAVDRNESCKCDRSYMFMKCLMESEIKSFMKIM

>CsupOBP8

MGFSRAVLLAAFVAGAWAMDEEMAELAKMLHDNCGEETGADLSLVDKVNAGADLMPDPKLKCYLKCIMET

AGMMTEGVVDVEAVLALLPDDMRADHCDTAFLTQLCWQKANKADYFLI

>CsupOBP9

MNCSVIAIIFAFLSLTSAELELSDEIKEIIQHVHNECVAKTGVAEEDIKNCENGIFKEDEKLKCYMFCLMEEANLAD

DDGVVDYEMMVSIIPEQYTDRVTKMIFACRHLDTPEKSKCQRAFDVHKCSYSKDPEFYFLF

>CsupOBP4

MFRLFLCLVVFVTTSYGDLLTQERSRGATLKPISACCGIPELGDSKPLTECSKPKLLGPCNDIQCVFEKSGFLVDRNT

LNKDVYKKHLRKWAEAHDSWTEAVERAIADCVDKELRQYLDYPCRAYDVFTCTGIAMLKKCPQEAWKC

>LstiPBP

MGFSVRLLVVLVAVTIYGVNSSQDIIKQMTINFGKALDSCRKELDLPDSINADFYNFWKEGYELSNRQTGCAIMC

LSSKLDLVDPEGKLHHGNTHEFAKKHGADDAMAKQLVDLIHKCESDVPDDPDPCLKVLNIAKCFKAEIHKLNW

APSMDLIMAEVLAEV

>LstiGOBP2

MSSAWILLGLVMAAVSSVRGTAEVMSHVTAHFGKALEECRTESGLSPEILEEFQHFWSEDFEVVHRELGCAIIC

MSNKFSLLQDDTRIHHVNMHDYVKSFPNGEVLSEKMVSLLHNCETQYNDMTDDCDRTVKVAACFKADAKKE

GIAPEVAMIEAVMEKY

>AtraPBP1

MKMHLFVQVIAASVMLMAGVDSSPEIMKDLSINFGKALDTCKKELDLPDSINEDFYKFWKEDYEITNRLTGCAI

KCLSEKLEMVDADGKLHHGNAREFAMKHGADDAMAKQLVDLIHGCEKSIPPNDDRCMEVLSIAMCFKKEIHN

LKWAPNMEVVVGEVLAEV

>DpleGOBP1

MKDVTLGFGEALKLCREESQLTEEKMEEFFHFWRDDFKFDDRAVGCAIKCMSSHFDLLTDSHRMHHRNMDN

FIKSFPNGEVLSQQMVTLIHECEQQHDSEEDHCWRILRVAECFKSSCKKHGIAPTMELLMAEFVMESEAN

>DpleOBP1

MTRQQIKNSSKMLKKNCMGKNDVTEDMVGEIDKGKFIEDKNVMCYIACIYQMSQLVKNNKLNYEASIKQVDL

MFPPDMKEAMKASIENCKDISKKYKDICEASYWTAKCIYDDNPKNFIFA

>DpleOBP

MYILAVVKFLAALTICEAMTMKQIKSTGKMMRKTCQPKNNVADDKIDDIGTGVFIEEREVMCYVACIMKMAN

AIKNNKLNYEAAIKQADLLLPDEIKEPTKEAITACRKVADSYKDICEASFHVTKCIYNHNPSIFFFP

>DpleGOBP1a

MKKLTTGFVKAMEECKAELNLGDHIIQDFMNYWREEYELLNRDTGCAIMCMASKHDLITEDMKIHHENAHEF

AKSHGADDDLAKQLVQMIHDCEKQFTDITDDCSKTLEISKCFRTKIHELKWAPSMETILEELMTET

>DpleOBP4

MKKWFLELTVECSKEHPVTKEEIQMLKDHKIPDNKNVKCLMGCVFRKIGWLDDNGMFSFNNAYKTSEEEYPD

DKTKLEKAKNLYSLCEKVNTAEVSDGKEGCERSSLLAKCLIENSSKMGFVVQ

>DpleOBP2

MAVRGLLLLSAVLAATSVTVDGTAEVMSHVTAHYGKSLEECRKETGLSKEILEEFKHFWSDDFEIVHRSPFTVRL

HPHLHDDARMHHINMNDYVKGFPEGDVLADKLVQMIHKCEKEYDDIKDDCDRVVKVAACFRADAKKEGIAP

EVAMIEAVMEQY

>DpleOBP1a

MTAEQKQMVHQHFEQVGMECIKTHEITAEDVTNLRTRKIPTGENASCFLSCIFKHVGIMDDNGLLQKESAIELAKKVFDDEEELKLIEDYLHSCSSVNTATVSDGEKGCERSLLAYKCMIENASQFGIDL

>DpleOBP4a

MRIMCVVFVLSFGYVYGLTDEEVKKEFIKEVMTCTKDITVDMFDLMELEQLKVPTKTNVKCVLACAYKRVGTM

NKEGKYDIKEAYKISETMMKGDDKRIENGKKLADLCSKVNEADVRDGNKGCERAALLFKCVIENAPKLGFKV

>DpleOBP5

MECSKDYPLTDEDIAQLKDKQFPDKDDVRCLFACAYKKTGMMDDQGKLSVDGVNNLAKKYFSDDQDKLQKS

QKFTEACAGVNDEAVTDGEKGCERAALIYKCSIEQASQITIGYQTCKILAKRPRIGVLAQAQEQDRLNRDLFLHL

GNTMQQ

>DpleOBP6

MLNLLRIALFIVAVNADLLNHERTKGATLKPISVCCDIPELGDPKPLAECSNMKLQGPCSDVQCVFEKSGFLLDKQ

TLNKEGYRNHLMKWLEGHKEWKDGIEKAISDCVDVDLRQYLDYPCKAYDVFTCTGIAMLKKCPKDSWKC

>SinfPBP1

MADSRWWLASFICVMIMTSSVMSSKELVSKMSSGFSKGLDQCKAELNVGEHIMQDMYNFWREEYELVNRD

LGCMVMCMASKLDLVGEDQKMHHGKAEEFAKSHGADDELAKQLVGIVHACETQHQAIDDPCSRTLEVAKCF

RSKMHELKWAPTMEVAIEEIMTAV

>SinfPBP2

MALHRSPTMSARSVLVLIASLFVIVKCSQEVMKNLTHNFSKPLEDCKKEMDLPDSVFTDFLNFWKEGYEFTSRH

TGCAIVCLSSKLELLDPDLKLHHGKAQEFAQKHGADEAMAKQLVGLIHGCMEAIREPADDPCMRALNVAKCFK

AKIHELNWAPSLDLIVGEVLAEV

>SinfPBP3

MRSNNIIFSVLLMSVGVREIEPSKDAMKYITSGFVKVLEECKKELNMNDHIIADLFHYWKLDYTLLNRDTGCAIIC

MSKKLDLLDVNGRMHHGNAREFALKHGAGDEVAGKIVDIIHDCEKKFERDDDECLRVLEVAKCFRTGIHELDW

QPKVEVIMSEVFTEI

>DtabPBP1

MTKTYTFLAVAIVLLAIDSRVDSSQDVMKDLSVKFGESMNQCIKEMDLPDVSADFYNYWKEDFVITRRETGCLF

SCLAKKVSMQHSDGLLHKDNTHNFATKHGADDEMAAKLVETIHACENSISESDDCVRVLSIANCFKKEMHKLN

WAPSAELVTQELMTIL

>DtabGOBP2

MLVYLVPLVIGLVMEPVVGTAEVMSHVTAHFGKALQECRDESGLSPEILEEFQHFWSEDFEVVHRELGCAIICM

SNKFSLLQEDTRMHHVNMHDYVKSFPNGQVLSEKLVQLIHNCEKQYDSITDDCERVVKVAACFKVDAKKEGIA

PEVAMIEAVMEKY

>DtabGOBP1

MRWTQLALLALCCLMQARGDQTVMKDVTLGFGQALEKCREESGLTDEKMEEFFHFWHDDFKFVHRELGCAI

LCMSRHFNLLTETSRMHHENTDNFIKSFPNGEILAAKMVEIIHTCELRFENEADHCARILRIAECFRDTCKSVDLA

PTMEILIAEFILQAESGKR

>GmolPBP3

MARFSILFSLVIIAVTVKEIELSSDAMKAITSGFLKVLEQCKQELNLQGHVISDLYHYWKEDYSLLNRDTGCAIICM

SKKLDLIDASGKLHHGNTAEFAAKHGAASEVASKLVEILHACEKTHDAIEDDCMRALEIAKCFRTDINQLNWTPK

MDVIITEVLTEM

>GmolPBP2

MAASGRWRMLLAVFVLSVCVNRVTPSADIMKKLTTGFATALDKCKNELNVQENVMQDCYNFWREDYELLNR

DTGCVILCMALKFDLIDEDAKLHHKNAHEFAKTHGADDDLAKQLVAMIHDCEKQNSDADDCIRTLGIAKCFRTK

IHGLKWAPSMETVLEEVMTEVKPS

>GmolGOBP1

MSKNLVRLLLALTAVAVAQATQEVLKDVTLGFGEALEHCRESTGLTTEKMEEFFHFWSDDFKFELREVGCAIQC

MSKYFNLLTDGERMHHENTDKFIKSFPNGEVLAKQMVTLIHTCEQQFDDMEDHCWRILRIAGCFKTGCQERG

IAPSMELIMAEFIMEADA

>GmolGOBP2

MALYWMVTVLLVVGGRMVDGTAEVMSLVTAHFGKALEQCREESQLSPEVLDEFHNFWREDFEVVHRELGCA

IICMSNKFSLLQDDARMHHDNMHDYILSFPKGDVLSAKMVELIHNCEKQYDDISDDCSRVVKVAACFKVGATQ

AGIAPEVAMIEAVLEKY

>CpomPBP2

MAAAAKWRTLVAAFILTLCVNRVTPSAEIMKKLTTGFATALDKCKTELNVQENVMQDFYNFWREDYELLNRDT

GCVIICMALKFDLIDEDAKLHHKNAHEFAKTHGADDDLAKQLVGMIHECEKATTEADDCIRTLDIAKCFRTKIHG

LKWAPSMETVLEEVMTEVKPS

>CpomGOBP2

MALYWLVGLVLVGGKMVDGTAEVMSHVTAHFGKALEQCREESQLSPEVLDEFHNFWREDFEVVHRELGCAI

MCMSNKFSLLQDDARMHHENMHDYVKSFPQGDVLSAKMVELIHNCEKQYDDIPDDCSRVVKVAACFKVDA

KKAGIAPEVAMIEAVLEKY

>CpomGOBP1

MSHIARLVLSLAAVALAHATVEVMKDVTLGFGEALEHCRESSGLTEEKMEEFFHFWHDDFKFEHRELGCAIQC

MSRHFNLLTDGQRMHHENTDKFIKSFPNGEVLSKTMVTLIHSCEQKFDDMEDHCWRILRIAECFKSGCQERGL

APSMEMMMAEFIMESEV

>HevirPBP

MVFHRSTMMSVRLVLVVAVGLLIRVDASQDVIKNLSMNFAKPLEDCKKEMDLPDSVTTDFYNFWKEGYEFTN

RQTGCAILCLSSKLELLDQELKLHHGRAQEFAKKHGADDAMAKQLVDLIHGCAQSTPDVADDPCMKTLNVAKC

FKAKIHELNWAPSMELVVGEVLAEV

>HevirGOBP1

MRGELRALLLLAAAAPLLADVNVMKDVTLGFGQALDKCREESQLTEEKMEEFFHFWRDDFKFEHRELGCAIQC

MSRHFNLLTDSSRMHHDNTEKFIQSFPNGEVLARQMVELIHSCEKQFDHEDDHCWRILHVAACFKGACVQRG

IAPSMELMMTEFIMEAEAR

>HevirGOBP2

MTSKSCLLLVAMATLTTSVMGTAEVMSHVTAHFGKALEECREESGLSAEVLEEFQHFWREDFEVVHRELGCAII

CMSNKFSLLQDDSRMHHVNMHDYVKSFPNGHVLSEKLVELIHNCEKKYDTMTDDCDRVVKVAACFKVDAKA

AGIAPEVAMIEAVMEKY

>CmedOBP1

MDGKNLPSGVFLIILVILLSSDFSFGMTRQQLKNSGKIMKKSCMPKNDVTEDQIGEIEQGKFIEERNVMCYVAC

VYTMTQVVKNNKLSYEAVVKQVDMMFPPEMRDAVKAAAAHCKDVAKKHKDLCEASYWTAKCMYDFDPKN

FVFP

>CmedOBP2

MLLVILAKFLMVLAMCNAMTMKQIKNTGKMMRKSCQPKNNVADEKIDPLGDGVFIDEKEVKCYMACIMKM

ANTMKNGKPNYEAAIKQVDLLLPEEIKQPAKEALAACKKVPDAHKDPCDAAFHVTKCIYNHNPSIFFFP

>CmedPBP1

MGFLVKLVLLAMVVGVQSSQDVMKKVTVHFSKALETCKKELDLPDAINTDFFNFWKEDYELQNRLTGCALMC

MSSKLDLVDPEGKLHHGNAHEYAKSHGADDSVAKQLVDLLHGCESSTAQSDDDCSRVLGIAKCFKAEIHKLKW

APDME

>CmedPBP2

MWAKTLMVVVTVVMMSVNVESSQTLLKDMTKNFLKAYGQCQKELGLPDSTATELMNFWKEGYEIKSREAGCAIMCLSKKLEVIDPEGKLHKGKTTEFIVAHGTDEATAHKLIDILHACMQSVTPSEDHCLMSLQVAMCFKAEIHKL

GWAPDTELLFEEMVAEMQ

>CmedPBP4

MEVEMLPEGMKQLTGGFIKVFEACKTELGLKDGMLTDMYHLWREEYDQVSPDAGCMFGCMSKKLDLLDAS

GKIHHGNTKEYVMQNGGGEDLAAQLLSISQECEKQHEGVEAECARMLEMAKCFRSGIKRVQWSPKMEVVIT

EIIADV

>CmedGOBP2

MLSSWVLLGLMMAAVASVKGTAEVMSHVTAHFGKALEECREESGLSPEILEEFKHFWNEDFEVVHRELGCALI

CMSNKFSLLQEDTRIHHVNMHDYVKSFPNGEVLSDKMVELLHNCEKQYDAITDDCDRTVKVAACFKKDCQKE

GIAPEITMIEAVIERY

>CmedGOBP1

MRLEAALLCALAAAALVEGSHKIMSDVTLGFGQALEHCREESGLTEDKMEEFFHIWSKDFKFQDRELGCALRC

MSKHFNLITDANELHHENTENFIKSFPNGEQLAKELVVLIHNCEKQFEGVEEHCMRTLRVGECIRDACLSRDLAP

TMELLLAEFIMQTEA

>DpunPBP1

MTKTYTFLAVAIVLLAIDSRVDSSQDVMKDLSVKFGESMNQCIKEMDLPDVSADFYNYWKEDFVITRRETGCLF

SCLAKKVSMQHSDGLLHKDNTHNFATKHGADDEMAAKLVETIHACENSISESDDCVRVLSIANCFKKEMHKLN

WAPSAELVTQELMAIL

>DpunGOBP2

MLVYLVPLVIGLVMEPVVGTAEVMSHVTAHFGKALQECRDESGLSPEILEEFQHFWSEDFEVVHRELGCAIICM

SNKFSLLQEDTRMHHVNMHDYVKSFPNGQVLSEKLVQLIHNCEKQYDSITDDCERVVKVAACFKVDAKKEGIA

PEVAMIEAVMEKY

>DpunGOBP1

MRWTQLTLLALCCLMQARGDQTVMKDVTLGFGQALEKCREESGLTDEKMEEFFHFWHEDFKFVHRELGCAIL

CMSRHFNLLTETSRMHHENTDNFIKSFPNGEILAAKMVEIIHTCELRFENEADHCARILRIAECFRDTCKSVDLAP

TMEILMAEFILQAESGKR

>DsupPBP1

MTKTYTFLAVAIVLLAIDSRVDSSQDVMKDLSVKFGQSMNECIKEMDLPDVSTDFYNYWKEDFVITRRETGCLF

SCLAKKQSMHHSDGELHKDNTHKFATIHGADDEMAAKLVETIHTCENSISESDDCVRVLGIANCFKKEMHKLN

WAPSAELVTQELMAIL

>DsupGOBP2

MLVFLVPLVIGLVMEPVVGTAEVMSHVTAHFGKALQECRDESGLSPEILEEFQHFWSEDFEVVHRELGCAIICM

SNKFSLLQEDTRMHHVNMHDYVKSFPNGQVLSEKLVQLIHNCEKQYDSITDDCERVVKVAACFKVDAKKEGIA

PEVTMIEAVMEKY

>DsupGOBP1

MRWTHLTLLALCCLMQARGDQTVMKDVTLGFGQALEKCREESGLTDEKMEEFFHFWHDDFKFVHRELGCAIL

CMSRHFNLLTDTSRMHHENTDNFIRSFPNGEILAKKMVEIIHTCELRFEDEPDHCARILRIAECFRDTCKSADLAP

TMEILMAEFILQAESGKR

>DhouPBP2

MAVLTKWRMFVVFIYLNLNNVQSSQDIIKSMTSSFGKLVTECQTELELGDEIIQEFVNYWSEDYQLVNRDIGCM

ILCMASRLDLVNFTDMTLHHGNAHEFAKSHGADDATAGQIVIIIHDCEKANIDEGDLCTRIMKVAKCFKVNMH

DLKWAPSIEVILEEVMISV

>DkikPBP2

MAVLTKWRMFVVLVFLNLNNVQSSQDIIKSMASSFGKLVTECQTELELGNEIIQEFVSYWREDYQLVNRDMGCMILCMASRMDLIEFGDMTLHHGNAHEFAKSHGADDATAGQIVTIIHDCEKANMDEGDLCTRVMKVAKCFKVKMHDLKWAPSIEVILEEVMISV

>DhouOBP17

MKTFILIAVCLVAVQALTDEQKEKLKKHKTECLAETKPDEQLVAKLKNGDYKTESEPLKKYALCMMVKSELMTKD

GKFKKDVALAKVPDAADKPMVEKHIDACLANKGSTPQETAWRYVKCYYEKDPKHAIFL

>DhouOBP10

MFRPLFIYFTFIYFLFCDVQAQSNLDELQKNYLNFILECAVENPVTSDDLEELKNQKMPDKENVKCLFACAYKKA

GMMNDKGDLFIEGVQEINKKYFGDNPEKMNKSEQFIQACKSVNDSPVSDGNKGCDRAALIFQCSVEKAPDFE

LVV

>DhouOBP13

MGAYLFMCLMLTAVGLEAHTIHLTHTQKDKAHQLTMECMKESNVKPEVITEAKKGHYADDEKLKKFTLCFFQK

AGILTPDAKLNVDTALEKLPPGVDKAEATKVLEECKNKTGKDKADTAFEIFKCYHHGTKTHILF

>DhouOBP6

MSRTSVVIFWTLYLMGLFCPNNAAVTAEQKSKIHEKLEGIGLLCVKDNAITEDDITSLRARKVPGGPNAPCFLAC

MMKEIGVMDSDGKIQKESALEIAKTIFEDSEDLKNIEDYLHSCAHINTEEVSDGDKGCDRAMLAYKCMTENSSK

FGFDNV

>DhouOBP11

MVRSASLIVVALAVLSKYSLALSDEKKTQVYGLLFAKAVECGKEFPISDDEVLALKNKKIETDNAKCFAACTLKKAG

ALDEAGKLSSAGAVKAIQDTLTDPADIKNLEDFFAHCSSVNDEAVNDDKGCDRAKLIFDCFRDNADAYGFNLDF

>DhouOBP4

MLGIRVGIIALFVLQAYCFDDEVLELAKMVRESCAEETNVDISLVEKINTGAGLTPDPVLKCYIKCTMETAGMMS

DGVMDLEVVMTLMPDDLKNKHGAGLEACGTQKGADDCDTAYLTQVCWQKTCKSDYFLI

>DhouOBP9

MFSITLSTAFLYLIAIIQAGKEKPIFSDEIKEIIQIVHDECVEKTGVTEEDITNCENGIFKEDVKLKCYMFCLLEEASLV

DDEGVVDYDMLISLIPDAYHDRASHMLLSCKHLDTPDKDKCERAFDVHKCSYEKDPDLYFLF

>DhouOBP3

MYFQVILCFCMVGACYARTDTEIRAWFFRQGVECSKDHPVTVEEMKLMHQHQFPESRNSKCIGACVFRRAN

WLDSKGMFDLEQGIAFSEKEYADNPTKLEAGKKLFQFCTKVNEEPVTDGEDGCDRALLLSKCLIGNAKEYGFEVI

PTDGPEE

>DhouOBP1

MLFKVIIHLFVYWIFCEAMTMKQMKNSGKMMRKTCQPKNNVEDEKLDGIMKGEFLDDTNLKCYMACIMKM

ANAVKNGKINYEQSLKQADLLLPEEIKEEAKAAITTCKNAANGHKDICDASFHMTKCVYEANPSIFYFP

>DhouOBP16

MKIQIVVLFSILVVVSRAERTPYHLPEKETENLLQVGSQCLTETNVPTETWDAIKKDKYEHDDIYKKFLFCMMTKL

EFVDDQGHPIKNKLYGTFKEDDLKKVIDKCGADPKGDTPVDVTYSFYKCCIDNTNIRIAI

>DhouOBP7

MNLDSLLVTVLMFSNMLCVWSVTRQQLKNSGKLLKKACMPKHDVTEEQVGNIDKGTFIEDRNVMCYIACIYS

VGQAVKNDKIIHDAMIKQVNQMFPAKMKDAVKAAIENCRGVGKSTIRNKLTVFVWRLCHI

>DhouOBP2

MSIKYVCVALLAFTAYAIALTQEDKQAFLTSIAPLIGECSKEYGVNPEEIEAAKAAHSGESLKPCFVACFFKKLGVIN

DNGDFDPEASKAKGKQFFKDVEDQNKVSEIADICTSINAETVSDKTQGCDRSKLLMECYSKNKGGFSPFV

>DhouOBP14

MQIHSSVSVFVVCAILFNGQTNCARTVAKAPPELAGPILETISACGEETKNNPERIMAAMKDNKVFEDDAFLKFI

DCTFQKSGYAKPDGTLDIEKSVKIFPKNVDMKTVFENCNKDKGSTRVETTFKIYKCYQQTSPVQLVF

>DkikOBP14

MCLGTLVANTFGITQEDIRKFREVMRPIVSSCANQYGITLEQVIEARKAGTMEQLDPCLSECVFKKTGVINDKGL

YDPDTAIEKSKKYVTDPADVEKFNNIHKACIPVNDAPVGDSKDCERAKLLLSCLVKHKGEFSTLAL

>DkikOBP7

MFRPVFIYFTFIYFLFCDVQAQMNLDELQKAYLNYVLECAVENSVTPDDLEELKNLKMPDKENVRCLFACAYKK

AGMMNDKGELFIEGVQEITKKYFSDNPEKMKYSEQFIQACESVNDAPVSDSKKGCDRAALIFKCGVENVPDFE

FIV

>DkikOBP20

MKAFILIAVCLVAVQALTDEQKEKLKKHKTECLAETKPDEQLVAKLKNGDYKTESEPLKKYALCMMVKSELMTKD

GKFKKDVALAKVPDAADKPMVEKHIDACLANKGSTPQETAWRYVKCYYEKDPKHAIFL

>DkikOBP6

MCSAELCVLRDLFADKLEYDNDFNNVLVPAIRECTSEMGYTEDDLDTARKNQQLDDCMFICVFHKMGLMKDD

KMDLDETYALTEKFFKPDTDKEDIRKVSVDCYKVSDAKSENDKKCCDRARLIFDCLKDNIM

>DkikOBP13

MFSITLSTAFLYLIAIIQAGNEKPIFSDEIKEIIQIVHDECVEKTGVTEEDITNCENGIFKEDVKLKCYMFCLLEEASLV

DDEGVVDYDMLISLVPDAYHDRASHMLLSCKHLDTPDKDKCQRAFDVHKCSYEKDPDLYFLF

>DkikOBP13a

MKNLIFIITFALITFSLTNPIRAEDDKNKQTDQASKAIKEIDLNEVMAECNKSFTIQMSYVDALNKTGSFPDESDKT

PKCYVRCMLEKTGIMSSDGVFDLKRIPVVFANQHDGDFLVKDEVIASLCAERKEKCQCEKAYNFMKCFRTTEIN

YYEKETQ

>DkikOBP11

MNLDSLLVTVLMFSNMLCVWSVTRQQLKNSGKLLKKACMPKHDVTEEQVGNIDKGTFIEDRNVMCYIACIYS

VGQAVIKNDKIIHDAMIKQVNQMFPANMKDPVKAAIENCRGVAKNYKDICEASYWTAKCMYDYDPSNFVFA

>DkikOBP15

MTRAQVKNTMTMMKKQCLPKTGATEDKVARIEEGVFIEEHSVMCYIACVYKAIQVVNNDRLDMGLVSKQIDL

LYPQNMKEPAKKAAGQCVSIQDKYSDMCEAIFYATKCYYEADPSSFIFP

>DkikOBP19

MKLQIVVLFSVLVVISSAERTPYHLPPKETENLLQVGAQCLTETNVPTETWDAIKKDKYEHNEIYKKFLFCMMSK

LEFVDNQGHPIKNKLYETFKEDDLKKVIDECGADPKGDTPVDVTYGFYKCCIDHTNIRIAI

>DkikOBP3

MLSYKYPIFIVFSHFDVVAPNMYDKRIQICIQALSLILLLVGDCYAMTKQQLKKSGKMMKKSCMPKNDVTEDEV

GEIENGKFIEEKNVMCYIACIYTMGGVVKNNKIALDLMLKQIDTMFPAEVKDELKAAANSCKDTLKKYKDICESS

YFMAKCLYDTAPDAFLFP

>DkikOBP18

MLHCKTTVLVFVFVSTVYADMKRPFQFPKERIDNFMNTASTCNKELNTNIDVSELFKGKYDQSESFKDSFYCIAV

NSGFYDANGWPKLEKLYEIYKDEDVRTVLKDCTADLDGERPKDLASNYLICFLVKAPITVIF

>DkikOBP16

MQIHSSVSVFVVCAILFTGQTNCARTVVKAPPELAGPILETISACSRETKNNPERITSAMKENKVFEDDAFLKFID

CTFQKSGYAKPDGTLDIEKSVKVFPKNVDMKTVFENCNKDKGSTRVETTFKIYKCYQQTSPVQLVF

>DkikOBP5

MCRTSAVIFCTLYLIGLFGSNHAAVTAEQKSKIHEKLEGIGLLCVKDNAITEDDITSLRARKVPSGPNAPCFLACM

MKEIGVMDSDGKIQKESALEIAKTIFEDSEDLKNVEDYLHSCAHINTEEVSDGDKGCDRAMLAYKCMTENSSKF

GFDSV

>DkikOBP22?MYGVVAIFTFLAVAWGDTPSDPSRVYCGFTPDSIYACLHNPKVIKQDVSVKCDKSTSECDRMNCIFRESGWLDG

SNVDKQKVSAYFDQFAKDQPEWSTAVQHLKTDCLNKDLPAQGVILNCPAYDIVHCALTAFIKNASPSQWSTAEQ

CAYSRSYASACPVCPSSCFAPQVPIGSCNACYLPPRTPQS

>DkikOBP23

MVGQSICLLVAVILQVISAETPLNVLNPLHRECYRPPHNVERPHECCKLTSFYKEEDFKECGLDKIEEGEKSGYRH

GPPDCTKSNCLLAKNDMLKDDKPDLEKIKAYITNWADKNPAFKDAVDDAITKCIKEDLPGPPHACLASKLAGCLT

FRLFLKCPAENWESSANCDSVKEHIEKCK

SLFENPPQ

>DhouGOBP2

MLVFLVPLVIGLVMEPVVGTAEVMSHVTAHFGKALQECREESGLSPEILEEFQHFWSEDFEVVHRELGCAIICMS

NKFSLLQEDTRMHHVNMHDYVKSFPNGQVLSEKLVQLIHNCEKQYDSITDDCERVVKVAACFKVDAKKEGIAP

EVAMIEAVMEKY

>DhouGOBP1

MRWTHLTLLALCCLMHARGDQTVMKDVTLGFGQALEKCREESGLTEEKMEEFFHFWHDDFKFVHRELGCAIL

CMSNHFNLLTETSRMHHENTDKFIKSFPNGEILAKQMVEMIHTCELRFESEPDHCARILLVAECFRDTCKSTNLA

PTMEILMAEFILQAEAGKR

>DhouPBP1

MAKAYTFLAVAIVFLAIDSRVDSSQDVMKELSVRFGESMNLCIKEMDLPDVSADFFNYWKEDYVITRRETGCLF

SCLAKKVHMQHPDGLVHKDNTHNFATKHGADDEMAAQLVEVIHSCENSISESDDCIRVLGIANCFKKEMHKL

NWAPSAELVTQELMAIL

>SfruOBP

MSKFACLVLCVVAVSLSGVHATAEEKAAFIEAVKPHIQECSKEHGVTPEEIKSAKAAGNADGINSCFLSCVYKKAE

VINDKGEYDADKALEKLKKFVSNEDDYAKFAEIGKKCASVNEKSVSDGDAGCERAALLTTCFLEHKSEIPA

>PaegOBP

MVGFVALISLSLLAVCNADIGVDPPVKCGSMPPSVYSCLGTPRVVNPKLAAQCDKTLPECEKLTCIFRKSGWMD

GDKVDKVKLTAYFDQFATDNPDWQPAVDNLKTTCLQGDLPAQGVLLNCPAYDAMQCTFASFLKHAQPSQWS

TSESCNAARQYAASCPVCPTDCFASQIPVGSCNACLVLPRSP

>SnonGOBP1

MQEVVRALVLLSVAGALADVNVMKDVTLGFGQALDKCRQESDLTEEKMEEFFHFWREDFKFEHRELGCAIQC

MSRHFNLLTDSSRMHHTNTEQFIQSFPNGEVLAQQMVLMIHSCEKQFDHEEDHCWRILHVAECFKQECVSKG

VAPSMEMMMTEFIMEAEAR

>SnonGOBP2

MTTKCSLLLVVIAAVTSSVMGTAEVMSHVTAHFGKALEECREESGLSAEVLEEFQHFWREDFEVVHRELGCAIIC

MSNKFSLLQDDSRMHHVNMHDYVKGFPNGELLSQKLVDLIHNCEKQYDSIPDDCDRVVKVAACFKVDAKAA

GIAPEVAMIEAVMEKY

>SnonOBP1

MMKNSVVCLYFAVMAVNFWNVKCMSDEEKKAVLETLTPIVEGCASDYGLTNEDFKKHENIDDDNFLPFKKCLL

QKLEIMDETGKYNSDAISKSIVDYTGDKEQAKKFKEQFDSCFRKNGNNDGDDEASQMKRVFVMFKCFKEMEE

>SnonOBP2

MLLIKFVKFLILVAMCEAMTMKQIKNTGKMMRKTCQPKNNVEDDKIDPIAKGIFIDEKEVKCYMACIMKMAN

TIKNGNFNFEAALKQADLLLPDEVKEPAKEAIIACKKAADGHKDICDVSFHITKCIYNQNPGIFYFP

>SnonOBP3

MKSFTVFCVVLVAGIHAANVTLPPNQKDKARQIGAECIKETGVTTEILAEAKKGPIPEDESLKKFTYCFFKKAGIIDNDGKLNIDVALAKLPPGVDKDDAKKVLEDCKSKTGKEPQDTAFEIFKCYHAGTKTHILLTDA

>SnonOBP4

MKCLLVFAACILLAQALTDEQKEKLKKHRTECLTETKVEEQLVNKLKGGDYKTDNEALKKYALCMMVKSELMTK

DGKFKKDVALAKVPNAADKPTVEKLIDACLANKGNTPQQTAWNYVKCYHEKDPKHAIFL

>SnonOBP5

MLKFTCLALCVVAASISRVYAGEEEKAAFREAIKPIIEECSKEHGVSTDEIHSAKTAGNAEGIKPCFLGCVYNKAAVI

NDKGEYDVDTALLKLKKFVPDEEKYAKLAEVAKKCASVNDKPVTDGEAGCERAALLTACFLENKAEAFI

>SnonOBP6

MSKFTCIVLCVVAASLTKVSHAVTEEEKAAFHEVMEPIVDECAKEHGVSEAEIKAAHEAGSADSIKPCXLAMYHE

KTEMLDSKGLFDAEKSLSKLKKYVKNDEEFAKYEEIGKSCMSVNEKAVSDGEAGCERAKLIIDCFLVXHKADVPF

>SnonOBP7

MFTGTLPVVLCLCAVVYGGKDKPVFSDEIKEIIQTVHDECVGKTGVAEEDITNCENGIFKEDPKLKCYMFCLLEEA

SLVDDDGTVDYDMLVSLIPDEYYERTTKMIFSCKHLDTPDKDKCQRAFEVHKCSYGKDPELYFLF

>SnonPBP1

MADSRWWFASFICVIIMTSSVMSSKELVSKMSSGFSKVLDQCKAELNVGEHIMQDMYNFWREEYELVNRDL

GCMVMCMASKLDLVGDDQKMHHGKAEEFAKSHGADDELAKQLVGIIHACETQHQAIEDPCSRTLEVAKCFRS

KMHELKWAPTMEVAIEEIMTAV

>SnonPBP2

MALHRSPIMSARLALVLIASLFIVVKCSQEVMKNLTHHFSKPLEDCKKEMDLPDSVITDLHNFWKEDFEFTSRHT

GCAILCLSSKLELLDPDLKLHHGKAQEFAQKHGADEAMAKQLAGLIHGCMETIREPADDPCVRAQNVVMCFK

AKIHELNWAPSLDLIVGEVLAEV

>SnonPBP3

MRSNNIIFSVLLMAVGVREIEPSKDAMKYITSGFVKVLEECKKELNMNDHIIADLFHYWKLDYTLLNRDTGCAIIC

MSKKLDLLDANGRMHHGNAKEFALKHGAGDEVAGKIVDIIHDCEKKFERDDDECLRVLEVAKCFRTGIHELDW

QPNVEVIINEVFTEI

>HmelGOBP1

MISLLTLSLACGVLTGLDATMEVMKDVTLGFGEALQSCREESQLTEDKMEEFFHFWRDDFKFEDREVGCAMKC

MSSHFNLLTDSHRMHHENTDKFIKSFPNGEVLSKRMISIIHTCEQQFDALEDHCWRILRIAECFKVACKKEGIAP

TMELLMAEFIMEADPS

>HmelGOBP2

MAVRCYVVVFLALLALATLPRVRSTAEVMSHVTAHFGKSLEECREESGLSSDILDEFQHFWSEEFQVVHRELGC

AIICMSNKFLLMHDDARMHHVNMHDYIKSFPQGELLSEKMVNLIHNCEKQFDDIEDECSRVVKVAACFKESAK

EEGIAPEVAMIEAVLEKY

>HmelPBP_C

MFKRLTVVVLVFVGLNKVTSNETLRGITASFLKVLEECRQELNIGDNVLADMYYFWKLDRTLIHRDTGCAIVCMS

KKLNLLDTSGKLHHGNAEEFALQHGAADDMAKKLVTTVHECEQKHELEEDQCLRALEIAKCFRGAMHEINWA

PKVDVAISEILTEV

>HmelPBP_D

MAVQKWQLILVMCVLMQACKVSPSQEIMHKLTKGFATAFEQCKQELNLGDNIMQDFLNYWREEYELLNRDT

GCAIMCMAQKHDLLTEDGIHHEKVHGFTKSHGADDELAKQLVTMIHECEKSNAGVSDECMKTLEVAKCFRTKI

HELKWAPDMETILEEIMTDI

>HmelOBP3

MVGLKALHDIQINKDTIITRNMNLKTDNKISHNHDPDWSYSSFPKEVKSHVEQFKRNMSECLKEVQLNDKRQ

VRRLSPKKESPVHGECLIACVLKRNGVIENGKIYKDNLLSLVRKFYGKDEKLMKKLEKNVDRCIEASVKNKDDCTVASYLNECTNDLMANNKHKIIVNY

>HmelOBP6

MENISYKGKLFLLLFIAFAHVTLVDSYSHKFFSQNLDTEPSLSIQYARDKKSDMITNECLTEMYPKNIYRYPLHIDR

NDVPCIIHCVLKKFGIMSNDGVINTRNYYRRVRAIHRYDPRVLISDVGETCAQNINGMNLDHDVCKKAKVFND

CTQLYAISYRESDD

>HmelOBP8

MLIHAVLALIGYTLAAQHRSFSGTMVDLSDPKVQGHLDALVRMAQSCVIKVRASPKDVRAYFTNSPPITRSGQC

FAACMLEQSDVINHGKVNRELLVHLAGLVNGKNSRVVRKLYGISRLCLDSIEGMSDRCQLASTYNDCLNENMIE

FAFPLDIAEEAVRKMPFHLIQPK

>HmelOBP12

MKIIIIITLALLTFSVAQCGSSVGNRMSTDDITTTTMATTMEDDDARSNFDVMSVMVDCNDTFRVEMSYLESLN

KSGSFPDETDKTPKCFVRCVLEKSDIVSGDSQFNVTRTAEVFSQIRDTSQNDIIKMATACSDRPEKCKCERSYQYL

KCLLETTIEINEMKSSK

>HmelOBP13

MQGFIVHCSIVAILVVGASALEGEMAELAKMLRDSCIEETGADIALIDKVNDGADLMPDPKLKCYIKCVMETAG

MMSQGAVDVEAVVAVLPPELQRHADKLRACGTKAGANDCDTAFLTQACWQGGCKEDYILI

>HmelOBP14

MKTLKLLILLFVFFFGDSQGMTDDEMREEFMRITMICSKDYKVDMKDLLSLQQLNIPTKKDVKCLLACAYKKTG

SMNKEGLYDIEASYRIAEMTKNGDPKRLENAKKLVDICAKVNDETVSDGEAGCDRAGLIFKCVVENAPKVKNN

>Hmel‐OBP16

MFLIVLIFQGYSDEERTKIYATMLPHILQCSTEYGITEDELKASKENEKFGSINPCFMGCIFKKIHVINKEGIFNVEK

AEKLSENFLVHDEDKKKASAVIKACATINDEDVSDGEKGCDRAKLLFECLLPFRQQVILKLIMCIEKATKT

>Hmel‐OBP17

MTSLQLVFLVFGVVAVSLGSVSAFSDEERNKIYAGMLPLVLECSKDYGLTEDDLKAAKESGSIGSINPCLMACVFK

KINVINDKGLFDVDKAGELSQKFLTETDDQQKATEIIKTCASVNEKDVSDAEKGCDRSKLLFDCLLPFKGQVKYNI

VIILIIYSSR

>Hmel‐OBP18

MTRQQLKNSSKLLKKNCMAKNDVTEDLVGDIEKGKFIEDQKVMCYIACIYQMSQLVKNNKLNVEASIKQVDM

MFPPEMKDATKASIENCKDVSKKYKDLCEASFWTAKCLYEDNPKNFFFA

>Hmel‐OBP19

MTRPQLKKTLTIMKNQCMPKHRVTNEKVGQIEQGVFVEDHDVMCYIACVYKTAQVVKNKRLDKDLVSKQIDIL

YPAEIREAVKLSTVKCIPVQYNYEDECEGIFYSVKCLYEDNPANFIFP

>Hmel‐OBP20

MTRQQLKNSGKLMKKSCMPKNDVTEDQIGQIEQGKFLEERNVMCYIACVYTMTQVVKNNKLNYEATIKQVD

LMFPPDMKDAVKAAVEHCKDISKKYKDICEVSYWTAKCMYDFDPNNFVFP

>Hmel‐OBP21

MTMKQIKNTGKMMRKTCQPKNNVADEKIDPLNKGEFIEEKEVMCYVACIMKMANTIKNNKLNYEAAIKQAD

MLFPDEIKEPAKEAITACRKVVDDYKDLCESSFYTTKCIYNYNPSIFFFP

>HmelOBP22

MGNSIKKDEEQHAMFRIKFYYIVIFLIIFTAFILVMAFTPLTKDEQIDKFNKMNEGVEPFRRNLTECARQVKASMV

DVENFLKRIPQTSLQGKCFVACILKRNSIIKNNKILKENLLEANKAVYGEDSEVLSRLKLAINECSDVVANIFEICEFS

SVFNDCMHMKMEHILDKVIMERRMEALGQMTGDPDVWTDEEDELLKLVKDEL

>HmelOBP23

MQSAALLAAVFLALITFGFGQKEKPEFSEEIKEIIQHVHNECVAKTEVTEEDIANCENGIFKEDIKLKCYMFCLLEEGSLVDENDNVDYDMMISLIPEQYTDRVSKMITACKHLDTPDKNKCQRAFDVHKCSYDSDPKVNF

>Hmel‐OBP2

MSILLCFLFLSIALIDGKTVNIPVDPDITSQLVQISADCIASNGLTEEVLKQVMEWKLENNEPTKKLLFCFGTKLNT

TDKNGHVILNEALKLAVSKKRPVFGDAIKRCNDQEGSDKYDTLFKIIICMRDQENIFLRF

>HmelOBP9

MLVFILFSAHNMENKCDEDVSPTCVPSVDPIEECIEKLHVNRALVEKLKSGNSKLFDKNLKRWLLCFFEKTCVMT

PDGVLRQDVVLKDIPDQDKSKIEKITSICLYQKLHFAVDTAWNYLNCFREKDPKYSVIANKI

>HmelOBP10

MKTFIVLAICFVAAQALTDEQKDKLKKHKSECLAETKVDEKLVDKLKTGDFEIENEPLKNYTLCMLVKSGLMSMD

GEFKKDVALAKVPNAADKQLVGQLIDLCLANKGAAPEETAWNYSKCYHQKDAKHSIFQ

>Hmel‐OBP11

MKTFIVLAICFVAAQALSDEQKDKLKKHRSECLAETKADEQLVNKLKTGDFKTENEPLKKYSLCMLIKSELMTKD

GKFKKDVALAKVPNAADKPAVEKLIDLCLANKGNTPHQTAWNYSKCYHEKDPKHSIFQ

>Hmel‐OBP24

MRAVWCLLAVSLAVVSGKVLDTLVIVPPERVPGIVQASSKCIEEMNLDKDTMQKFFSWQLGDSESTRKYMYCL

GVKSGYIADDGSMVKKEVLGLAGSHGGNIDGVIDECNNLKYSDKYEAVFKIVMCFHEKSKLEFKV

>HmelOBP27

MDTGKMRAVWCLFLVTLATVYAGNVKVEYVDIPKDYIPAVEKASFECIKKLKLEEEQISLQGFLNWELSESDNTK

KYIFCLANGSGFFADDGSILKDKVLAIMGKYRDRVDKVIDECSKVKYDNKYEEVYRMEVCFRDLSGLYFRM

>Hmel‐OBP28

MNTLWFFLFLSIALVKGKALFHVPPEYAGEILKAAADCIDSTGAGVDAVQKVISANLENTEPFKKFLYCFSSKSGYV

DSDGHFIVDQMTKLIGNHKDKAKFIDNLNLCNKSEGGNTIDTIYQIAVCFKDNSPIYFTV

>Hmel‐OBP29

MKTFWCFLFLSIALVSGRAIVNVSQEFTGDILKTAVDCTDSSGAGVDALQKMASANFEDTEPFKKFLYCFASNSG

YVDSDGHFIMDKMTKLIGNHKDKAKYVDAINLCNKRKGGRTIDTIYELANCFKDHSPIYFTL

>Hmel‐OBP30

MFDIPKEFVPDIIKASAECADKLGLDTLNILSKFFSGELKDSESVRQYMYCLGTTSGYVNDDGRLNKDRLGKVVG

EHKSKVESVVDECNKAKASDKYETVYKAVVCFRENSGLQFKI

>HmelOBP31

MLMRKNPICIYISIIYLGVQYVLVEYIKVPKEFIDDAINGSGQCAEELGLPGDTLNKLLSNNFEDSQAMRKYIYCLG

IALDVGDGTGSLKHSLSKYASNDRRKAEITKTIDECNKEKASDKYEKAYKVSTCYLNTSSVQFKV

>HmelOBP35

MKVLIICVGLFALIEAHNIHLSHGQKEKVKEYAAECMKESGAKPEVLADAKKGHLVDDEGLKKFILCFFQKTGVLS

SDAKLNTDVALSKLPAGIDKVTAAKVLNDCKNKKGATHADTAFEIFKCYYTHTKQHILFEK

>Hmel‐OBP1

MSVRVLFIFIIVTACQANIQVSPPVTCGYLPRAIHECIGSPHIVKPEISAQCSKSISECERMTCVFQKSGWMSGNA

VDKDKVKSYFDQFSTDNPQWALAVNHVKAACLNMDLPSQGVYLNCPAYDILTCVFSGFIKNAQPDQWSSSES

CSYPRQFASACPYCPSDCFAAQVPIGSCNACLALPRSP

>AdisPBP1

MADSRWRFTRFVCVMLMTSSVMASKELMTKMSTGFTKVLDQCKSEVNVGDHIMQDMYNFWREEYELVNRDLGCLVICMANKLELIGDNQKLHHGKAEEFAKSHGADDDQAKQLVAIVHDCENQHQAIEDACARMLEISKCFRTKIHELKWAPSMEVVMEEIMAAAN

>AdisPBP2

MTVRLALVVIASFVIAVESSQEIMKTVSLNFAKPLEQCKKEMDLPDTVTTDFYNFWKEGYEFTSRHTGCAIMCLSSKLELLDQEMMLHHGKAQEFAMKHGADEATAKQLVDMIHSCSQSTPDAAEDPCMKALHVASCFKAKIHDLKWAPSIELIMGEVLAEV

>AdisPBP3

MGTYRVLLVIVVMAVALREVEPSKDAMKYITSGFVKVLEECKKELNMDDNILADLFHFWK

LDYALLDRNTGCVIICMSKKLDLLDDTGRMHHGNAQEFALKHGAGEEVAAKIVSIIHECE

KKFERDDDECLRVLEVAKCFRSGIHELDWQPKVQTIVSEVLVEI

>AdisGOBP1

MQAVRALVLLTVAGALRADVVVMKDVTLGFGQALDKCRQESDLTEEKMEEFFHFWRDDFK

FEHRELGCAIQCMSHFFNLLTDSSRMHHGNTEEFILSFPNGEVLARQMVELIHTCEKQFD

HEQDHCWRILHVAECFKSACVAHGIAPSMEMMMTEFIMESEAR

>AdisGOBP2

MTSKYCILVVVAAVASSVMATQEVMSHVTAHFGKALEECREESGLSAEILEEFQHFWRED

FEVVHRELGCAIICMSNKFSLLKDDSRMHHVNMHDYVKGFPNGEVLSARLVELIHNCEKQ

YDSLPDDCDRVVKVAACFKVDSKAAGIAPEVAMIEAVMEQY

>AdisOBP1

MFFKKSLLFLVAAVFVICESGFVENLGKCLASENDCDKDELQATIRDIASTNARQLVDTL

DKCKISDGNCQRDLIQSVIRDISKTGVKELGIPTIDPIELKNISFSVLNALDITLIEGSG

KGIKDCIVDRFFTDVEAERAFMELTCDITVKGRYKVFSNSPLIKTFTGGDTLSGDGNGKV

KIDKLHLRFDFDFFIQRRDGEIYIKCKDDKIKYTYNIGKMLFFADNLYIGKQESSALVTR

LLNDNWRMLLSTFGKPFMDKAMDYVYIFLHGFFETVPAKHFISDDLSKYARDA

>AdisOBP2

MVGAVVLLTLLPAWVACSGEGNIKLLEDEVAVALKSCTYPDDTVVSKEPVSKERQRRGSD

DTYDGSPRIDNNMREGNRYSHERRNTSNSGDQIQVFNATDYDYEGYGTGSNGEKLLTSVP

RPASPSNNVNYNNTSRTRRSEQLLNKQDLDQCLSQCVFANLQVVDTRGIPREAELWNKVQ

SSVTSQQSRSALHDQIRACFQELQSEAEDNGCSYSNKLEKCLMLRFSDRKVDGKASAQKP

ASAEQS

>AdisOBP3

MWNFVILFVTLCSCVYGLTEQELKVEFTKLVMKCNKNSEVEMTDLVQLQSYVVPTKTATK

CVLACAYKAASVMNAQGMYDIDHAYKVAELIKNGDEKRLINGKKMADICVKVNDIKVSDG

EKGCERAALIFKCTVENAPKFGFKL

>AdisOBP4

MTTYNFQRVFCILCIFCLFFGNTYAMTRQQLKNSGKLMKKSCMPKNDVTEEEVGDIEKGKFIESRNVMCYIACIYTMTQVVKNNKLSYEAVVKQVDVMFPAEMRDAVKAAAANCKDITKKYKDLCEASYWTAKCMYDYDADNFVFP

>AdisOBP5

MVARLLIIASVACFVNLANAIEPFPGFKKCKSSDSDCILENAIKGVSSFGDGIPELGVRR

LDPDFFDKVDASSPNLKFILSDITLTGLKSCKPTKVTRDAAKSQIFLILQCALKLDGNYE

LDGNVLILKIKGKGKIHVYLRNIVNTVTLDYVIKEGKDGQKHLRIKKFDHTYKLNEKSDV

VFEGLFQDNDVLSQAADDLIKNNGNEIVEEIGGGMFTAAVTRIVENFNKFFGKLPYEDIS

ID

>AdisOBP6

MFHLYISVFIYCVFSMSVKASSLDELKTKYVELILECSNSYPITGDDMSELRQKTMPDSEPVKCLFACVYKKAGMMNEHGQLSVQGVNEMTRKYLADDPEKIKKSEEFTQACESVNDVEVSDGERGCDRAALIFKCTVEKSPDFDLL

>AdisOBP7

MDRRRLFWLLIAMFLATGSDAMSRQQLKNSGKMLKKNCMKKNDVTEDQVGTIDKGKFVEDKKVMCYIACIYEMTNVIKNNKLSYESSIKQIDLMYPPDLKDSAKAAVEHCKDIQKKYKDICEASFYAAKCMYEFKPEDFIFA

>AdisOBP8

MNNKVFILLFLTYISLVSSSKAPFITKCKWDDAKCLKESSQAAIPLFAEGLPDLNVAKQE

PLIIKHVDASTSNLKLIITDVEVNGLKNCEARKITRDIENSKIIVKIRCSVDFKGKYDMK

GQLFILPIEGNGDLTAHIRKMLITAEVDIMDKTGKDGKTHWNVKSWRHSFDLQDKSDVRF

ENLFPDNELLRKSTEELIASNGNDVINEIGHPVIKSVVAAVINNIKKFFDTVPLEDLVLD

>AdisOBP9

MAGLLLSMVLTLTTVALTRSATTQMKDAGTKEAMSTTMADMDSKIDALDVNVLDVMSACN

ETFRIEEAYIQSMNETGSFLDENDKTPKCFIRCVFENVGIVSEDGKQFNPARAAVIFAGE

RNGKTMDDIGDMTAMCAADRQETCPCERSYQFLRCLMSMEIEKYEKA

>AdisOBP10

MNRLLLIFIIATCLEFSYGMTREQVKKTMTIIKKQCLPKHPVTDDQVGRIEQGVFIEDRNVMCYITCIYKSLQVVKGDKLDMGLITKQIDALYPPDLKDPVKHAVSLCIHSQDNYNDLCEKVFHAVKCLYEKDPPNFIFP

>AdisOBP11

MFRWVLPVVLCLFAATFGGKEKPVFSDEIKEIIQTIHDECVAKTGVAEEDITNCEKGIFK

EDAKLKCYMFCLMEEASLVDDDDVVDYDMLVSLIPDEYYDRTTAMIFSCKHLDTPDKDKC

QRAFEVHRCSYEKDPDLYFLF

>AdisOBP12

MLYSSTFFLFTVLLILMLDSPYVSSMTREQIKNSGKLIKKTCSAKNGLTEDQVKDVDKGKFIEEKNFMCYIACVYKMGQTIKGNTINHDMMLRQVDMMFPTEMKVPVKAAIEHCRGVAKNYKDVCEASYWTAKCTYEFDPANFMFP

>AdisOBP13

MKTLLVFATCILLAQALTDEQKEKLKKHRTECLTETKVDEQLVNKLKGGDYKTESEPLKK

YALCMMMKSELMTKDGKFKKDVALAKVPNAADKPMVEKLIDACLANKGNTPHQTAWNYVK

CYHEKDPKHAIFL

>AdisOBP14

MFKFCVFLVFCVASCYAAPGGTYCGETPDVIYSCLSAPKLVSSEISSKCTGSQYSNECDR

LTCVFREAKWLNGASVDKAKLTAYFDQFEKDHAEWAPAIQHVKTVCLANELKAQGVYLNC

PAYDIMHCALSSFIKHATPSQWSTAASCTYPRAYATDCPVCPSDCFSAQVPIGSCNACYL

QPPTA

>AdisOBP15

MFKYFIYCVFIVSTCHADLLSQKENKGATLKPLSVCCDIPELGDPKFLAKCSNPKLPGPC

NDVQCVFEESGFLTDRNTLNKEAYKAHLRKWEEDNKGWTVAVDKAIADCVDNEPRQHLDV

PCKAYDVFTCTGIAMLKKCPEAAWKC

>AdisOBP16

FKFCVFLVICVASCYAVQGTRTYCGETPSVLHRCLNLPTLVNVEASNKCSPSRYSSECER

LNCIADELKWRTDSSVDKAKVTAFFDQYETDHAEWAPAVQHVKTVCLASDLKAQGVYL

>AdisOBP17

MCGFKTVFYLLTVLNACYGAVDIRKYLKVCDRNAIDANDCMVDAVQKGIAVLVDGIEELA

VPPIDPYLQKEFRVEYKNNQIVVKLVINNIYVEGLRLAKVHDARLRADDDKFHLEVDLTG

PLVAVRAQYYGEGQFNNLKILAYGEFNTTMTDLVYTWKLNGVPEKNGNETYVRIKDFYMR

PDIGSIVTSFRNDNPETREFTDLGTRFANENWRTLYKEFLPYAQANWNKIGTKIANKLFL

KVPYDQLFPASP

>AdisOBP18

MIRSCSLVFAALVQVLVGQAQEPATFPQFQERIPRHCLAPPPGINLHTCCPIPSLYPDEV

MESCGIEKVRQDSPNAPPKPRGPPKVPCKEGICLMQHADLLLANQSVDYEKLRGFIDHWA

ESNPDFSEAILAAKEICAKDGGPAGPPVCEQDKIFFCLTSNILWNCKLRDLDGNSGCSIL

KAHMDECRPHFLKRKELEEQNGQ

>AdisOBP19

MKSFVVLCVVVVAGIHAADVPLPAAQQERAKIIAAECTKESGVSKAVLADALKGNLAEDE

GLKKFTFCFFQKAGVVDGQGVLNVDVALAKLPPGVDKANAKSVLEGCKSKTGKDTAEKVF

EILKCYHKGVANHVLFAGL

>AdisOBP20

MLLIEIVKFLILVATCEAMTMKQIRNTGKMMRKSCQPKNNVEDEKIDPIAEGVFIDEKEV

KCYMACIMKMANTIKNGKLNFDAAIKQADLLLPDEVKEPAKEAILACKKAADPHKDICDA

SFHVTKCIYNQNPGIFYFP

>AdisOBP21

MSKFTCLVLCLVAASISRVYAGEEEKAAFRAAIKPIIEECSKDHGVSPDDIESAKAAGSA

DGIKPCFLGCVYKKAEIINDKGEYDDDTALTKLKTFVNDEDKYAKLAEIGKKCASVNDKA

VGDGDAGCERAAQLTACFLENKGEALI

>AdisOBP22

MSKFTCFLLCIVAMSLSSVHVARGEKSLRDALRPVIVACSQEHGVTDAEIQAAKDAGSPE

TIKPCFIACVFKKAGFINDKGELDLETGLKNLRQFVKNEEQYNKYENVAKECVSVKDKPV

TDGKAGCERGALLAACFLDHRASIII

>AdisOBP23

MSKFTCIVLCVVAASLVKVSHAVTEEEKAAFREAMAPVIAECSEEHGVSESDIAAAKEAM

SADAIKSCFLGCVMKKTEAVDAKGLFDADAGLSKIRKYVSSDDDFAKFEKIGKLCMSVNE

KEVSDGEAGCERAKLVLACFLEHKADIPF

>AdisOBP24

MFVYGRLSFAAVLLCLGCTYAISKEDETSLKQVLRPHMLECAEEFGITEEQIEEAKKKGN

KHNMDPCFLACMMKKADFMNSDGKFDVEKTVSFAKDHSLSEKAVKFFETVGEECVKVNDE

EVGDGEKGCERAKLLFHCVHEIKKKMSE

>AdisOBP25

DGKQQKAAVLGKVATRADAKNATKVLESCAEQTGDTPEDLAWNLFRCGYDKKALLFNYMP

TNVASEPDNNS

>AdisOBP26

MWLRAMVLVAALAAARAVEMDEDMAELARMVRENCIAETGADVALVE

AVNGGADLMPDDKLKCYIKCTMETAGMMGDGEVDVEAVLALLPPALAAHNGPALHACGTV

RGADHCDTAWKTQVCWQTANKADYFLI

>AdisOBP27

EQKAQIHAHFETIGKSCNKDSTVITSEDIADLRAKKIPSGPNAPCFLSCMMKQIGVMDDN

GMLQKETLLDMAKKIFDDADELKIIEDYLHSCSHINGESVSDGAAGCERAMLAYKCMTEN

ASQF

>AdisOBP28

MRDRDDMMGRSDDRMDRNDDRNNRNDDRMSSNNERSGGRNRSGSKNNRNDKNGGRDDKYG

RDDYFNGREDFPLSDEYGSDMGQGQYNNYYSTTPSSRRYKRERRPENSGQRSQYNPNTHK

ILGYEDNFRSDEKNATENNSSKESDNKACALHCFLENLEMTAEDGMPDRYLMTHAFTKDV

KNEDLRDFLQESIEECFQILDNENTEDKCEFSKNLLICLSEKGRANCDDWRDDLKF

>AdisOBP29

EQKQQIQTKVVAVGAECIKEYPLSIEDLASFKSRVFPEGENAGCFSACIFNKLGLFDDKG

TWSHVTALEHAKKVFDDEEVLKNVESFLTTCAKVNEEEVKDGEKGCDRAKLAFDCFVKNY

EQLGFNFDF

>AdisOBP30

MKLLNRPYNDSPLHELKHIKQGASSFGANCLLSFKMVKFSVVCLYFAVVAVNLWNVNCVS

DEEKEAIIKAITPIAEDCAKDCGLSDKDNKKKKGDEDDMDPCFKKCLLQSLGLLDEDGKY

DRELLRDSIKEYTGDKEAATKVQDQLDACFDANGDNSGDDEESQMKRVDVMFKCLKEIKE

>AdisOBP31

MECNNEHPISPKEMLILKANKIPDSESAKCFVACIFKRTGMLDSKGMFDAAASIAMTEKD

FADDPKKLENSKKLLESCKNVNDEPVKDGEKGCERSVLLHKCIVDTAAQLGIKLPN

>AdisOBP32

MVRKIGGLLCCLCVFGISLSDSAISADSESRCRNPPTAPQKIERVITLCQDEIKLSILRE

ALDVIKEEHTMPAQRRRDKREVPFTHDEKRIAGCLLQCVYRKVKAVDGYGFPTLEGLVGL

YSDGVNERGYFMAVLEASRECLMKNHDKFSRTVPMDNGRNCDISFDIFECISDRIGEYCG

TAGL

>AdisOBP33

MTRSSSCFVLAAVIQVIFGAHFTMHRKPKHSLSIRHQNVPQNMPRYCWRPPDVDLDKCCP

TPNLYSDEVMASCGFDKPPVNRAGDPVRPKGAKLVLPLCRDGLCLMTSANLTLKNTDVDY

KKLDVYIDQWAAANPEFTKAILEVKPICAAKADEFWRKDQLRICDQDRIYICLASYLLWN

CKIKPFKDCRKLKHYIKTCRKYYDYSK

>AdisOBP34

MENHIFSIVLFVLFSFAFLAIISFEPMSKEEHMKKYNKMTEDIEPFKKNITECAHQVKAS

MADVENFLQRIPQANFQGKCFVACILKRNAIIKNNKISKEQLLETNRAVYGEDSEVMARL

KTAIRDCTKAVDGIFEICEYASVFNDCMHIKMEHILDQVMMERRMEAIGKITTNSEQWGE

TEDEIMKLVKDEL

>AdisOBP35

MFSRFFPKGLTEQELKVEFTKLVMKCNKNSEVEMTDLVQLQSYVVPTKTATKCVLACAYK

AASVMNAQGMYDIDHAYKVAELIKNGDEKRLINGKKMADICVKVNDIKVSDGEKGCERAA

LIFKCTVENAPKFGFKL

>AdisOBP36

MESCGIEKVRQDSPNAPPKPRGPPKVPCKEGICLMQHADLLLANQSVDYEKLRGFIDHWA

ESNPDFSEAILAAKEICAKDGGPAGPPVCEQDKIFFCLTSNILWNCKLRDLDGNSGCSIL

KAHMDECRPHFLKRKELEEQNGQ

>AdisOBP37

SDIINHGKINRELLVHLASLVNGKNSRVVRKLNTVSRLCLDSINGMSDRCHLASTYNDCL

NENMIEFAFPLDIAEEAVRKMPFHLIQPNLPQEARSNPFYKH

>AdisOBP38

MKSFVVLCVMAVAGIQAVDVPLSPVRQEKAKIIVAKCMKESGVSKAVLADALKENLAEDE

GLKKFTFCFFREAGVVDGQGVLKVDAALAKLPPGVDKANAKSVLEGCKSKTGKDAAEKVF

EMYKCYHKGVANHVLFVGVDI

>AdisOBP39

MKRLMFCSLILMSVAENERLMMQTISNYIGGMVLKCQKKMEFKNDVIQDLLEFWNKDNDL

RSEDLGCALVCVFEENDFLDSEFKEVQRDNVEGFFRASGAGDKMSQTLLQLFERCKTSTT

NIDNLCDSALQVAKCFRRGIFEQQWAPARQDTTTGA

>AdisOBP40

MFRQILLLFSIIYLATSEINKKIDENIPDQNRMMGIDAVHDNSIKIDKNTIITRNLKLEK

RNRGQKAISNKIEEDQEPYWSYESFSTEVAAHVEQFKKNMSECLKEVQSNDKRPLKRLSP

KMEAPVHEDCLIACVLKRNEIIANGKVNKGKGFEISTSSDCKLLMA

>AdisOBP41

MESCGYEKPPVNKAGEPIKPKGPKSILPYCRDGLCLMTNANLTLPNSAVDYKKLNAYIDQ

WAAANPEFTKIIMDIKPICAVEELRKKTKPRLCDHDRIYICLNSHLLWNCKIKPIKECDQ

LKMFMEACRSFYQKPRKKILD

>AdisOBP42

MADLEVAVTSIDVSKIPSCFWGCGFRKAGFLNDEGQYDVETGVSNLKRFMGDPTALEMLE

KVARQCNSVKDKPVSDGKAGCEMGKLAAACFLEQMKEMKMSK

>AdisOBP43

MLKKVDIVTSDGQLNVDVAVSKMPPGLDRNDSRKLLESCKTKTGKDGLNTVFEIFKSYFA

GTKYHVKLDFTNILVY

>AdisOBP44

MRTGFMNDKGQYVLYPGLTYVRQFTNYEAYTNLEAIAKRCESVKEETVSDGVAGCQMGSL

IAACFLREFLSQGIRF

>AdisOBP45

MSDFTSLVLCVVVVNLSIVYADDTSGANREDVQTILRECSIEYGVSEQSMNQAAISQDVT

TVDSCFWACALKKTGFLTDKGEYDMKTGMMYVKQVVPSETAYKNLEDIAKLCEIVKGKPV

NDGEAGCEKGAQVVDCFLKQMATQRKMAGH

>AdisOBP46

MYDAAASIAMTEKDFADDPKKLENSKKLLESCKNVNDEPVKDGEKGCERSVLLHKCIVDT

AAQLGIKLPN

>AdisOBP47

SCRDDFEVVEQECLKKHGLTTADMSAALNAKDASKMDPCFWGCYFKTLGLLNDKGQYDLS

TGLANIRKYMTDDARIAKVEETSKQCEKVTDVPVSDGDAGCEKGAHVAACFFGRALSDKA

>AdisOBP48

MLEQSDIINHGKINRELLVHLASLVNGKNSRVGRKLNTVSRLCLDSINGMSDRCHLASTY

NDCLNENMIEFAFPLDIAEEAVRKMPFHLIQPNLPQEARSNPFYKH

>AdisOBP49

MIKYIILVACLAASLNSLDKVGDGRRVKTCRASTKFRENQAKVIRYQMECVEQTRVDPDS

IVKIKKNRWSLPQNQDSLVKEWALCVLMKSGIMTKEGVYKADIALKRVPAMERHIVEKQI

DKCLTPKPVPAPEIAYRFIKCFQRHKSNHSATVSAFQ

>AdisOBP50

MVKFSVVCLYFAVVAVNLWDVNCVSDEEKEAIIKAITPIAEDCAKDCGLSDKDKKKKGDE

DDMDPCFKKCLLQSLGLLDEDGKYDRELLRDSIKEYTGDKEAATKVQDQLDACFDANGDN

SGDDEESQMKRVDVMFKCLKEIKE

>AdisOBP51

RTARSAGTLVDFTDSKVQGHLDALVRMAQSCVIKVRATPKDVRAYFTNSSPIARSGQCFA

ACMLEQSDIINHGKVIMAVLFVCD

>AdisOBP52

MQDFHVTPDDINRAAETGDPNIIPPCFNGCVFKKSGFINEKGEYDLDSGMAYLRPQVKDE

EQYNALKEVATECTRKDEVSDGDAGCERGGKLSACFLQNKSTVKV

>AdisOBP53

MYPRNLYKYPLRIDRNDIPCIIHCVLKKCGIMTNDGYINIRNYYKRVQSIHRNDPRILIS

DVGETCAQNINGMNLDHDVCKKAKVFNDCTQLYAVSYSDPDDLMM

>AdisOBP54

MTAEQKAQIHAHFETIGKSCNKDSTVITSEDIADLRAKKIPSGPNAPCFLSCMMKQIGVM

DDNGMLQKETLLDMAKKIFDDADELKIIEDYLHSCSHINGESVSDGAAGCERAMLAYKCM

TENASQFGIEV
