# Supplementary material for: Identification and comparative expression analysis of odorant-binding proteins in the reproductive system and antennae of Athetis dissimilis
Source: Sci Rep. 2021 Jul 6;11:13941. doi: 10.1038/s41598-021-93423-1 (PMC8260659; doi:10.1038/s41598-021-93423-1)
Supplement: Supplementary file 3 — Supplementary File 2. [file 41598_2021_93423_MOESM3_ESM.docx]

**Supporting information: S1 File.** Primers used for expression analysis by qPCR in this study. (DOCX)**. S2 File.** Amino acid sequences of OBPs used in phylogenetic analyses. (TXT)
